# Supplementary material for: Analysis of Antimicrobial Resistance in Bacterial Pathogens Recovered from Food and Human Sources: Insights from 639,087 Bacterial Whole-Genome Sequences in the NCBI Pathogen Detection Database
Source: Microorganisms. 2024 Mar 30;12(4):709. doi: 10.3390/microorganisms12040709 (PMC11051753; doi:10.3390/microorganisms12040709)
Supplement: Supplementary file 1 [file microorganisms-12-00709-s001.zip › Supplementary File S1.pdf]

Supplementary File S1

The following pages contain figures for antimicrobial resistance classes observed in bacterial species of select genera (title at top left of each page). For each species listed on the y-axis, percentage of isolates from each source (differentiated by colour) with each respective resistance class (top panel headings) is displayed along the x-axis. Sizing of points corresponds to the total number of isolates of that species for which isolation source could be identified (No. of isolates). Metadata files for whole-genome sequence data were downloaded from the NCBI Pathogen Detection database. Datasets were analysed and source categories were assigned based on reported sequence submission information. Data was excluded from analysis where source information was missing or incomplete (see methods). Quinolone class includes mobile genes (e.g. qnrS) and chromosomal poihnt mutations (gyrA, parE, parC) reported for some genera.

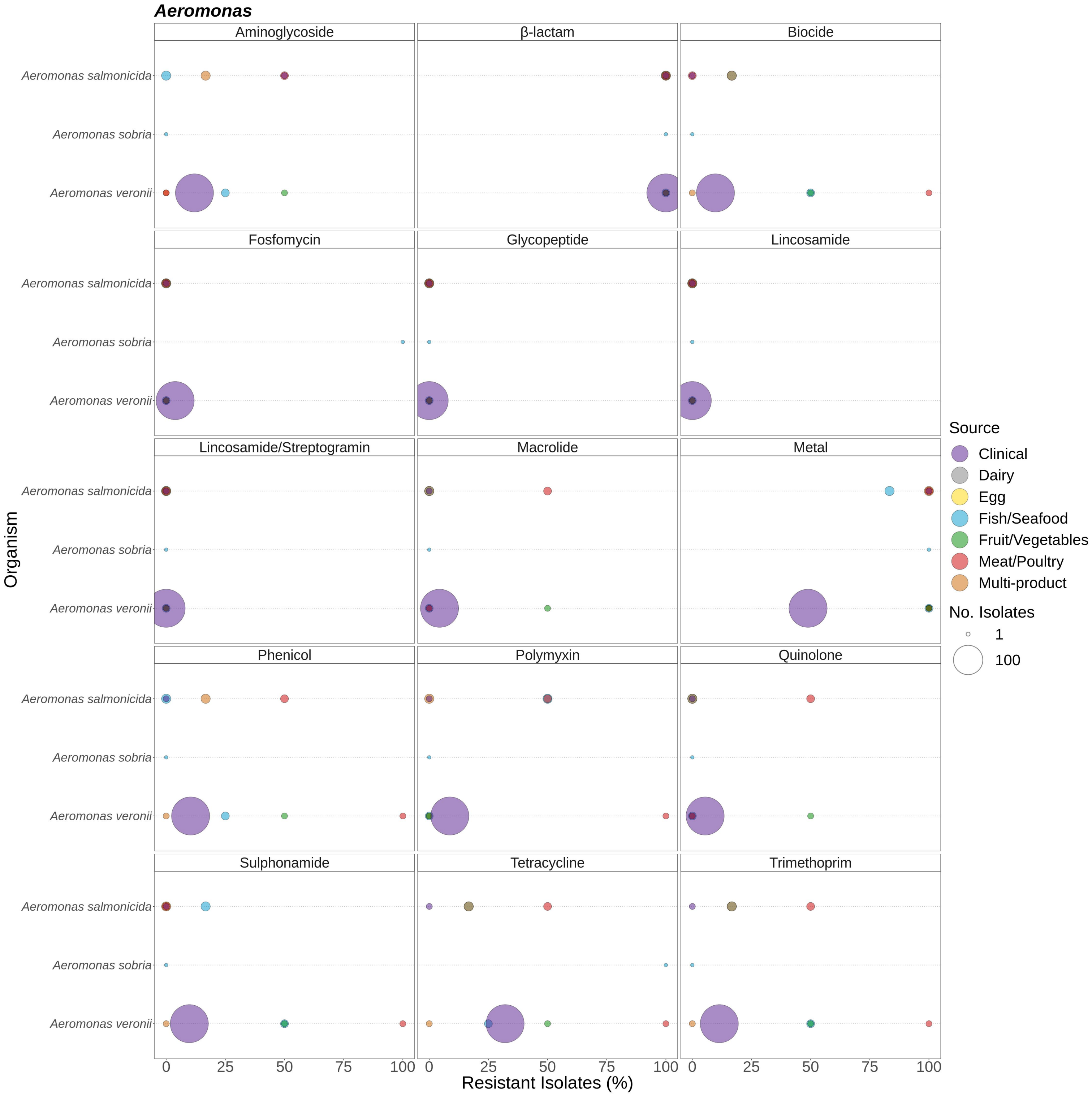
Aeromonas
Aminoglycoside
β-lactam
Biocide
Fosfomycin
Glycopeptide
Lincosamide
Lincosamide/Streptogramin
Macrolide
Metal
Phenicol
Polymyxin
Quinolone
Sulphonamide
Tetracycline
Trimethoprim
Aeromonas salmonicida
Aeromonas sobria
Aeromonas veronii
Organism
Resistant Isolates (%)
0
25
50
75
100
Source
Clinical
Dairy
Egg
Fish/Seafood
Fruit/Vegetables
Meat/Poultry
Multi-product
No. Isolates
1
100

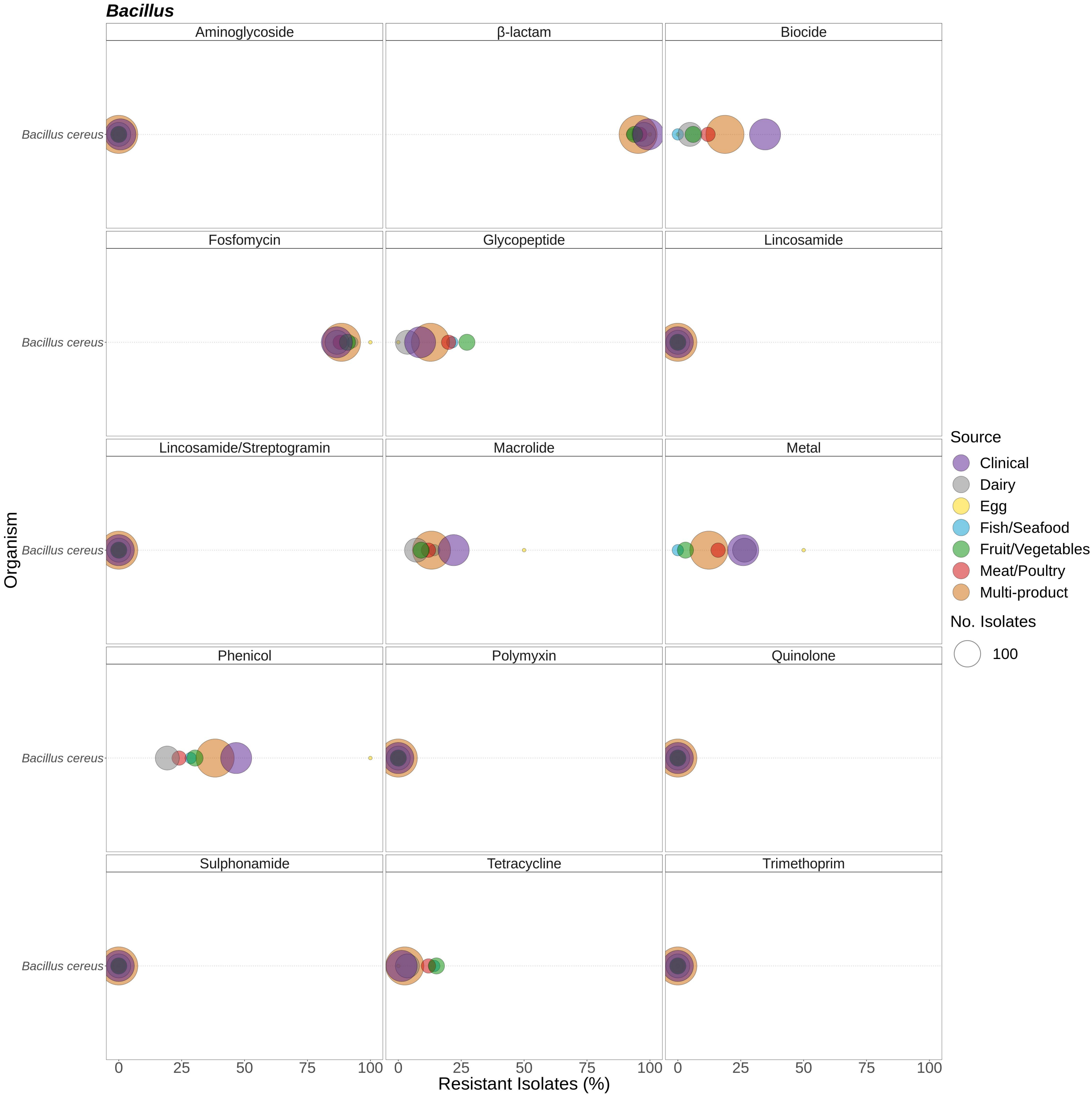

Bacillus
Aminoglycoside
β-lactam
Biocide
Fosfomycin
Glycopeptide
Lincosamide
Lincosamide/Streptogramin
Macrolide
Metal
Phenicol
Polymyxin
Quinolone
Sulphonamide
Tetracycline
Trimethoprim
Bacillus cereus
Organism
0
25
50
75
100
Resistant Isolates (%)
Source
Clinical
Dairy
Egg
Fish/Seafood
Fruit/Vegetables
Meat/Poultry
Multi-product
No. Isolates
100

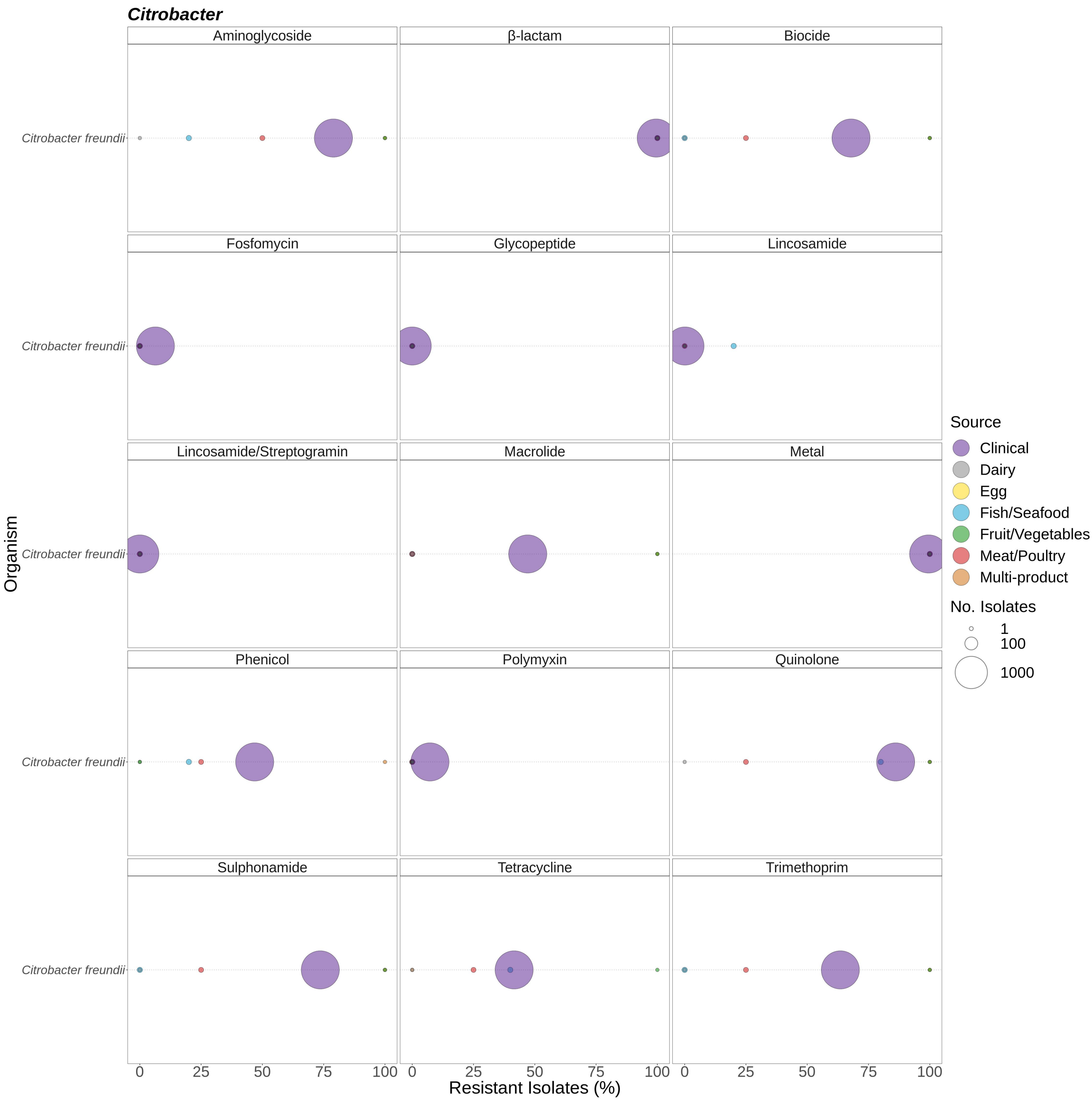

Citrobacter
Aminoglycoside
β-lactam
Biocide
Fosfomycin
Glycopeptide
Lincosamide
Lincosamide/Streptogramin
Macrolide
Metal
Phenicol
Polymyxin
Quinolone
Sulphonamide
Tetracycline
Trimethoprim
Citrobacter freundii
Organism
0 25 50 75 100
Resistant Isolates (%)
Source
Clinical
Dairy
Egg
Fish/Seafood
Fruit/Vegetables
Meat/Poultry
Multi-product
No. Isolates
1
100
1000

Clostridium

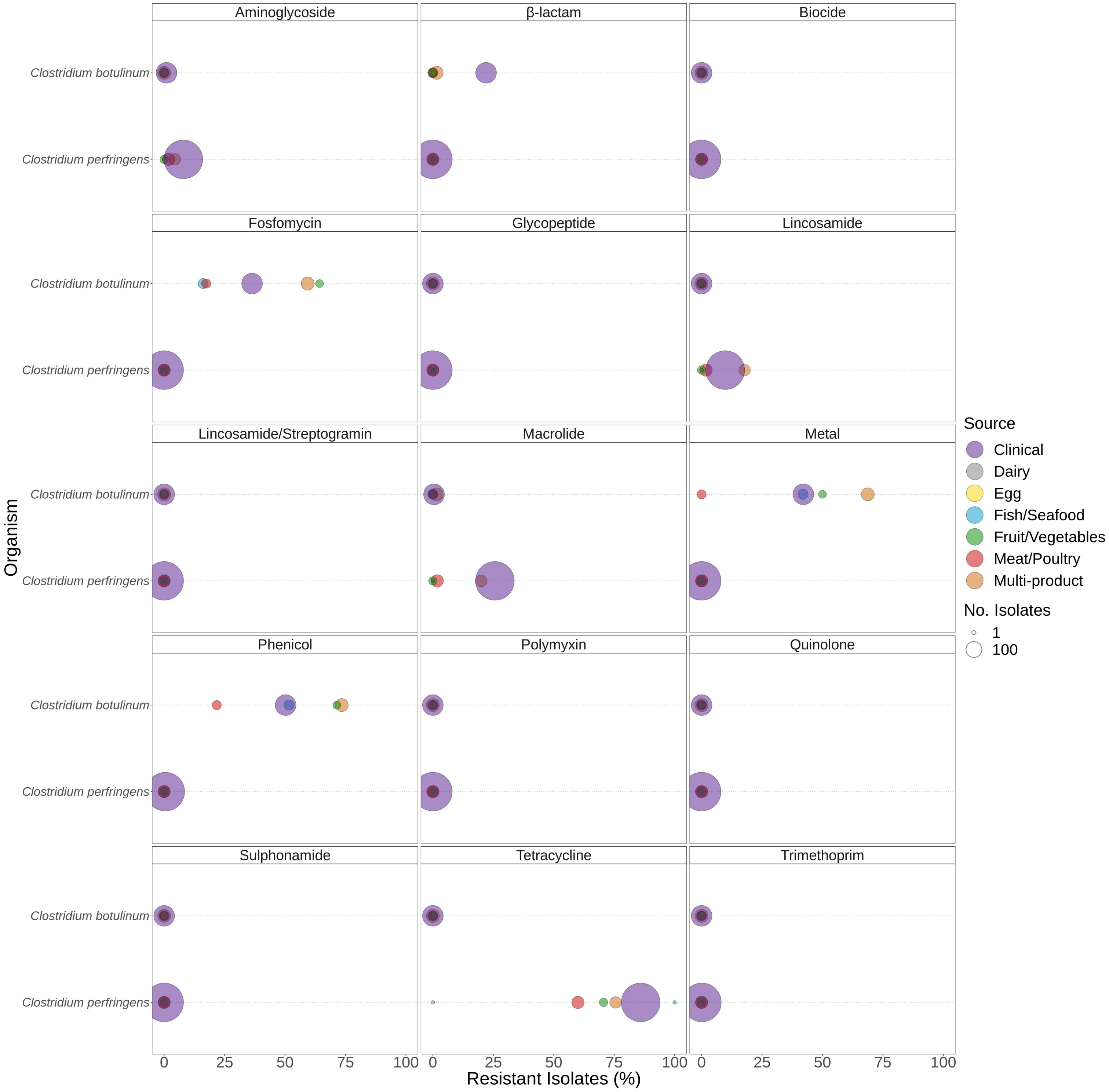

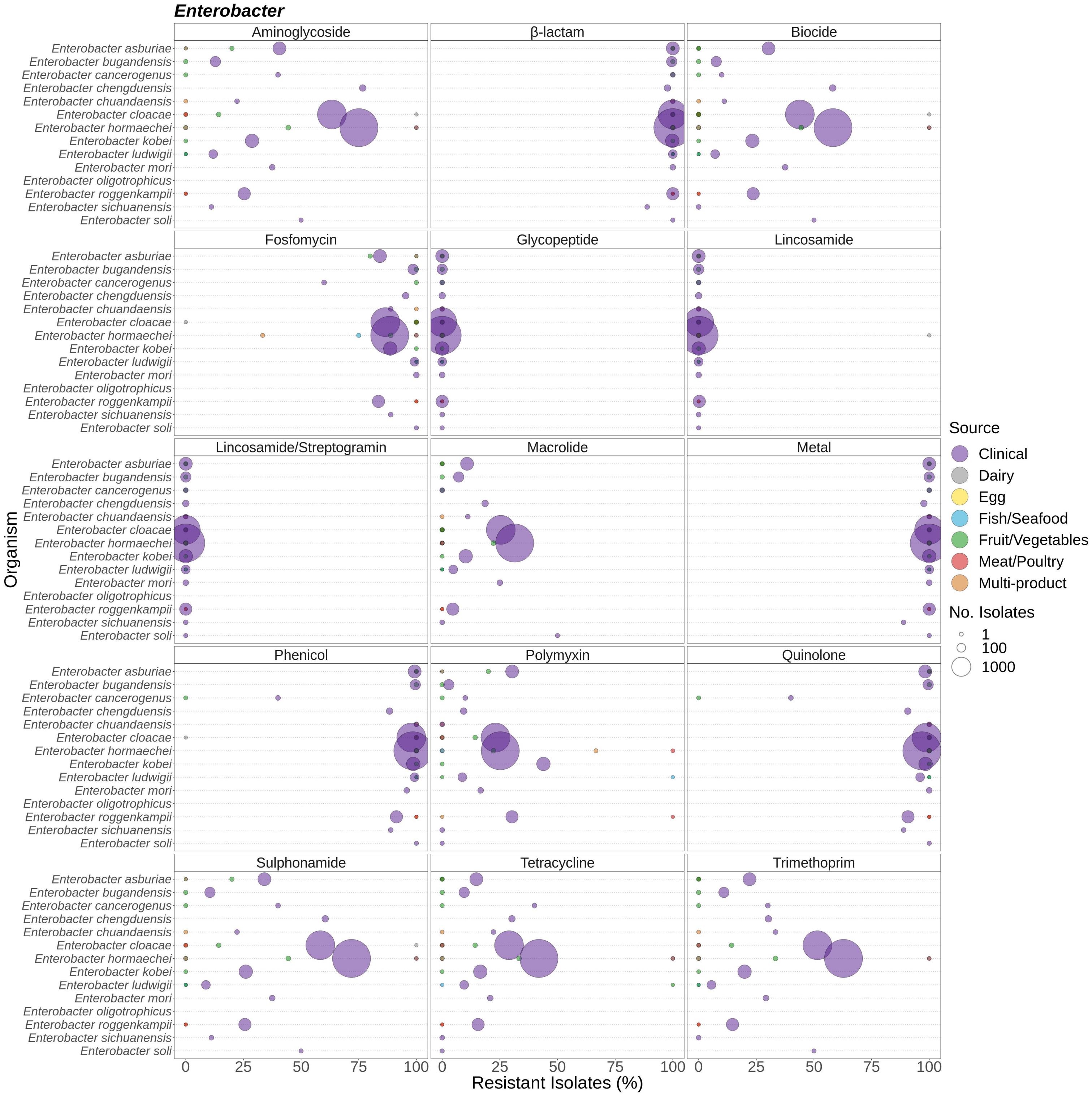
Enterobacter
Aminoglycoside
β-lactam
Biocide
Fosfomycin
Glycopeptide
Lincosamide
Lincosamide/Streptogramin
Macrolide
Metal
Phenicol
Polymyxin
Quinolone
Sulphonamide
Tetracycline
Trimethoprim
Enterobacter asburiae
Enterobacter bugandensis
Enterobacter cancerogenus
Enterobacter chengduensis
Enterobacter chuandaensis
Enterobacter cloacae
Enterobacter hormaechei
Enterobacter kobei
Enterobacter ludwigii
Enterobacter mori
Enterobacter oligotrophicus
Enterobacter roggenkampii
Enterobacter sichuanensis
Enterobacter soli
Organism
0
25
50
75
100
Resistant Isolates (%)
Source
Clinical
Dairy
Egg
Fish/Seafood
Fruit/Vegetables
Meat/Poultry
Multi-product
No. Isolates
1
100
1000

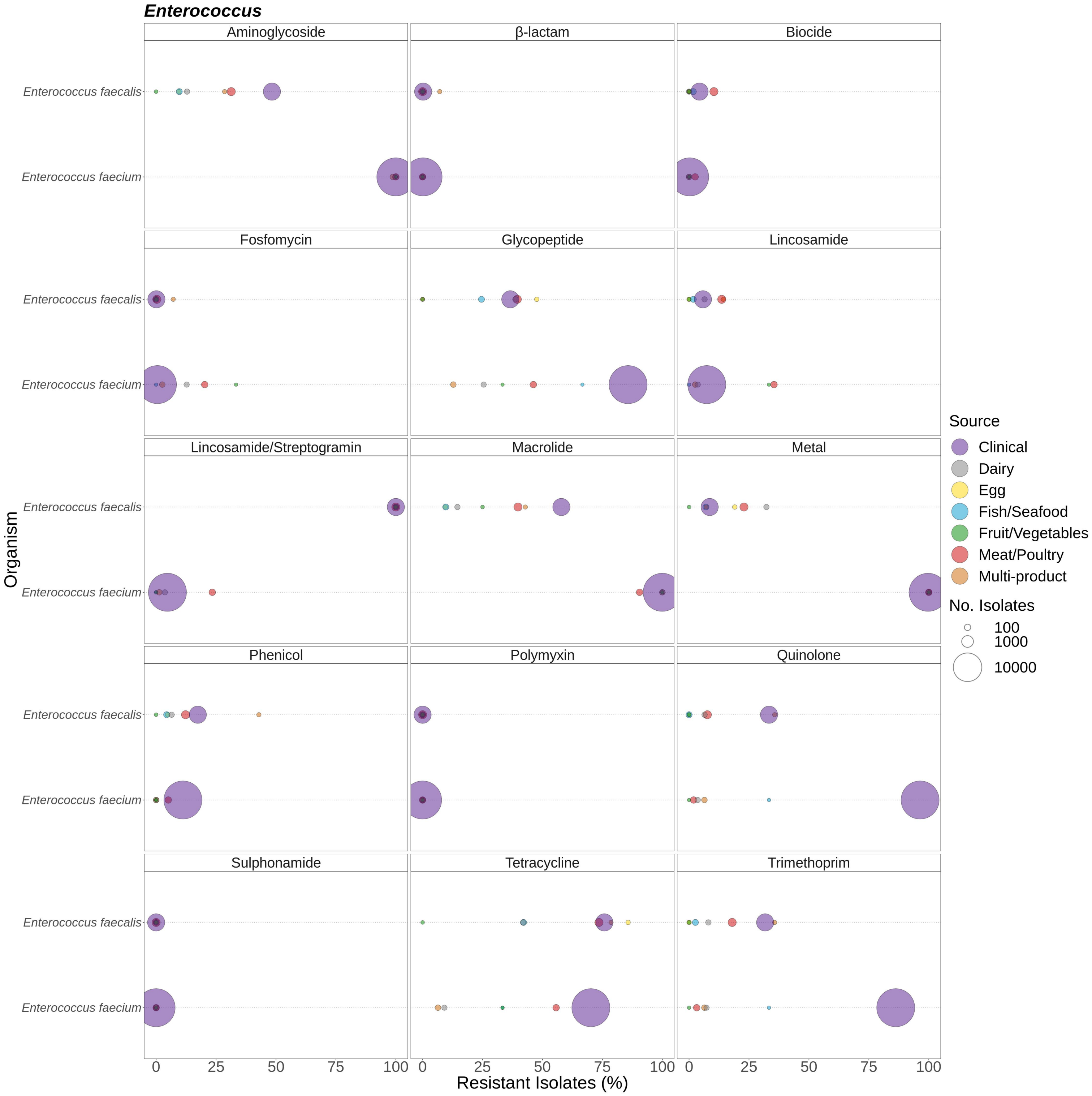

Enterococcus
Aminoglycoside
β-lactam
Biocide
Fosfomycin
Glycopeptide
Lincosamide
Lincosamide/Streptogramin
Macrolide
Metal
Phenicol
Polymyxin
Quinolone
Sulphonamide
Tetracycline
Trimethoprim
Enterococcus faecalis
Enterococcus faecium
Organism
0
25
50
75
100
Resistant Isolates (%)
Source
Clinical
Dairy
Egg
Fish/Seafood
Fruit/Vegetables
Meat/Poultry
Multi-product
No. Isolates
100
1000
10000

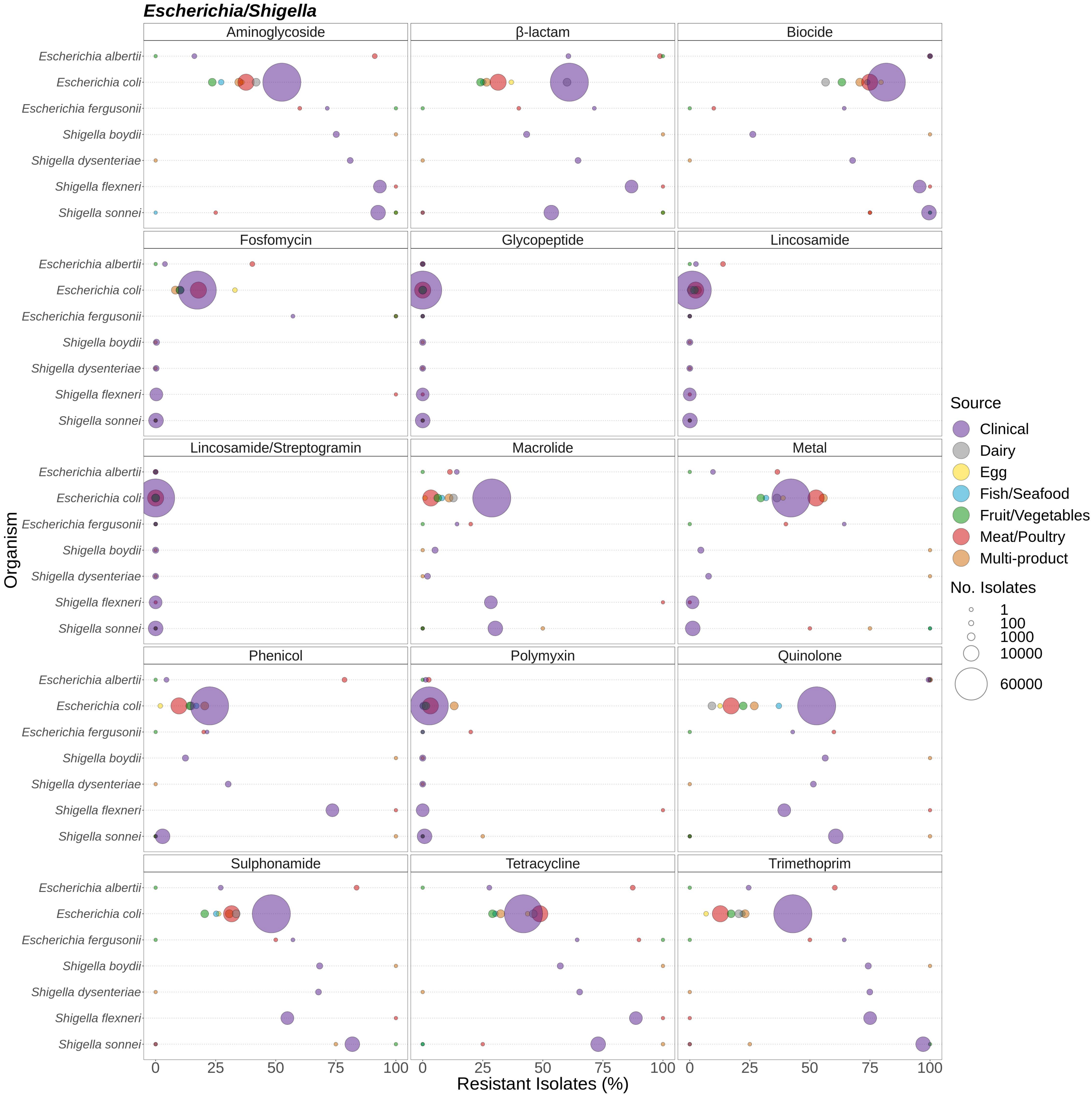

Escherichia/Shigella
Aminoglycoside
β-lactam
Biocide
Fosfomycin
Glycopeptide
Lincosamide
Lincosamide/Streptogramin
Macrolide
Metal
Phenicol
Polymyxin
Quinolone
Sulphonamide
Tetracycline
Trimethoprim
Escherichia albertii
Escherichia coli
Escherichia fergusonii
Shigella boydii
Shigella dysenteriae
Shigella flexneri
Shigella sonnei
Organism
0
25
50
75
100
Resistant Isolates (%)
Source
Clinical
Dairy
Egg
Fish/Seafood
Fruit/Vegetables
Meat/Poultry
Multi-product
No. Isolates
1
100
1000
10000
60000

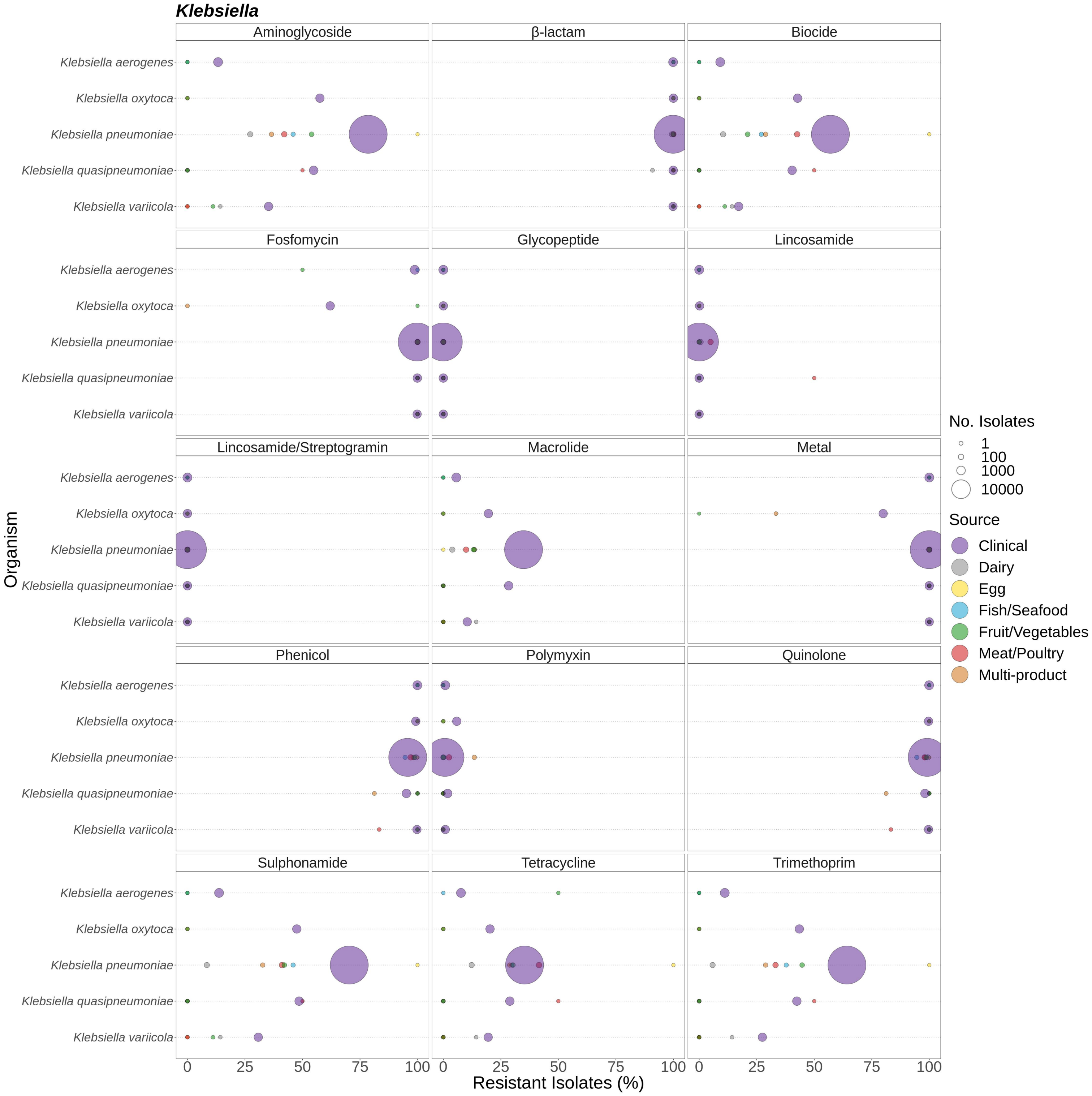
Klebsiella
Aminoglycoside
β-lactam
Biocide
Fosfomycin
Glycopeptide
Lincosamide
Lincosamide/Streptogramin
Macrolide
Metal
Phenicol
Polymyxin
Quinolone
Sulphonamide
Tetracycline
Trimethoprim
Klebsiella aerogenes
Klebsiella oxytoca
Klebsiella pneumoniae
Klebsiella quasipneumoniae
Klebsiella variicola
Organism
0
25
50
75
100
Resistant Isolates (%)
No. Isolates
1
100
1000
10000
Source
Clinical
Dairy
Egg
Fish/Seafood
Fruit/Vegetables
Meat/Poultry
Multi-product

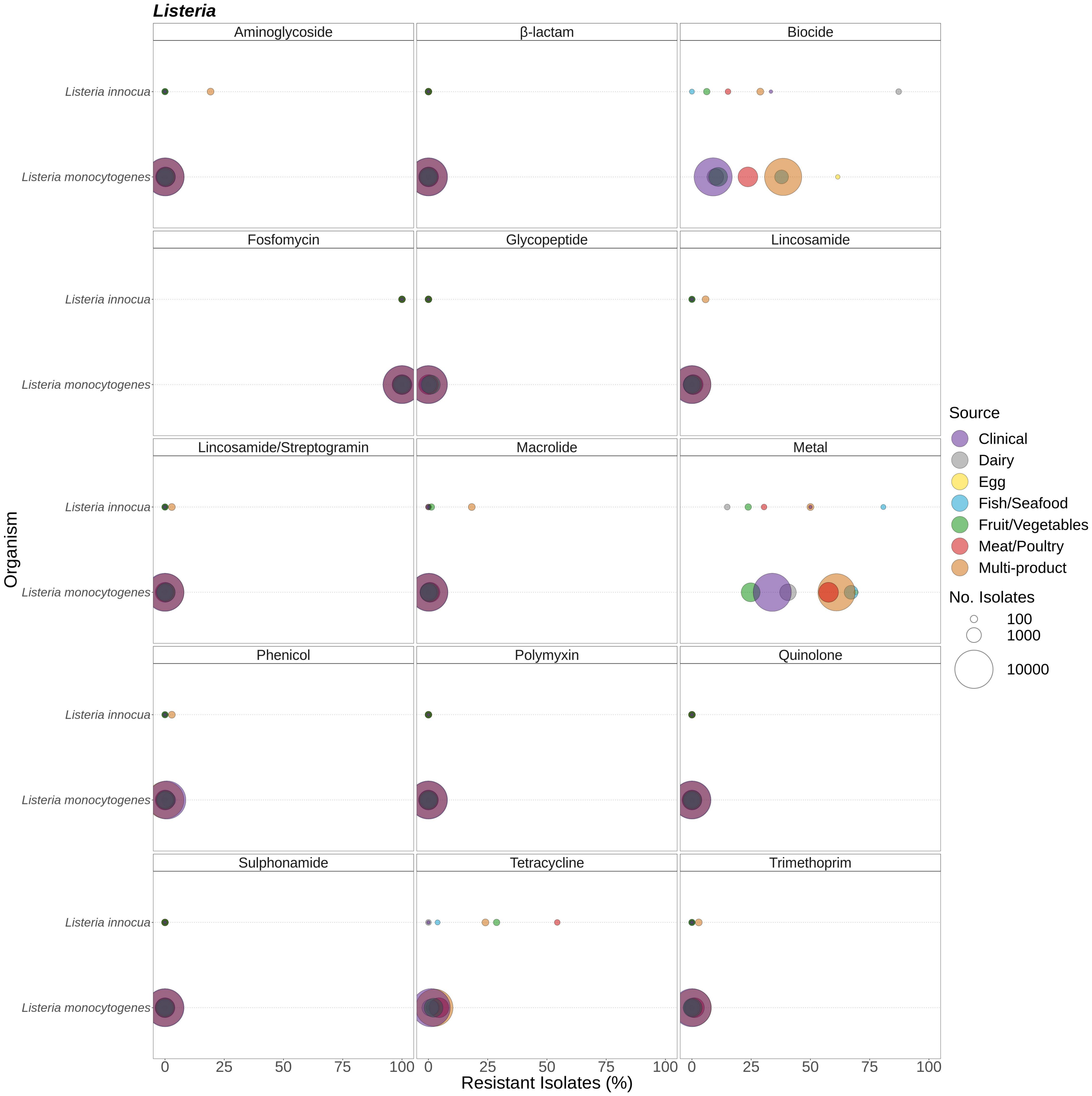
Listeria
Aminoglycoside
β-lactam
Biocide
Fosfomycin
Glycopeptide
Lincosamide
Lincosamide/Streptogramin
Macrolide
Metal
Phenicol
Polymyxin
Quinolone
Sulphonamide
Tetracycline
Trimethoprim
Listeria innocua
Listeria monocytogenes
Organism
0 25 50 75 100
Resistant Isolates (%)
Source
Clinical
Dairy
Egg
Fish/Seafood
Fruit/Vegetables
Meat/Poultry
Multi-product
No. Isolates
100
1000
10000

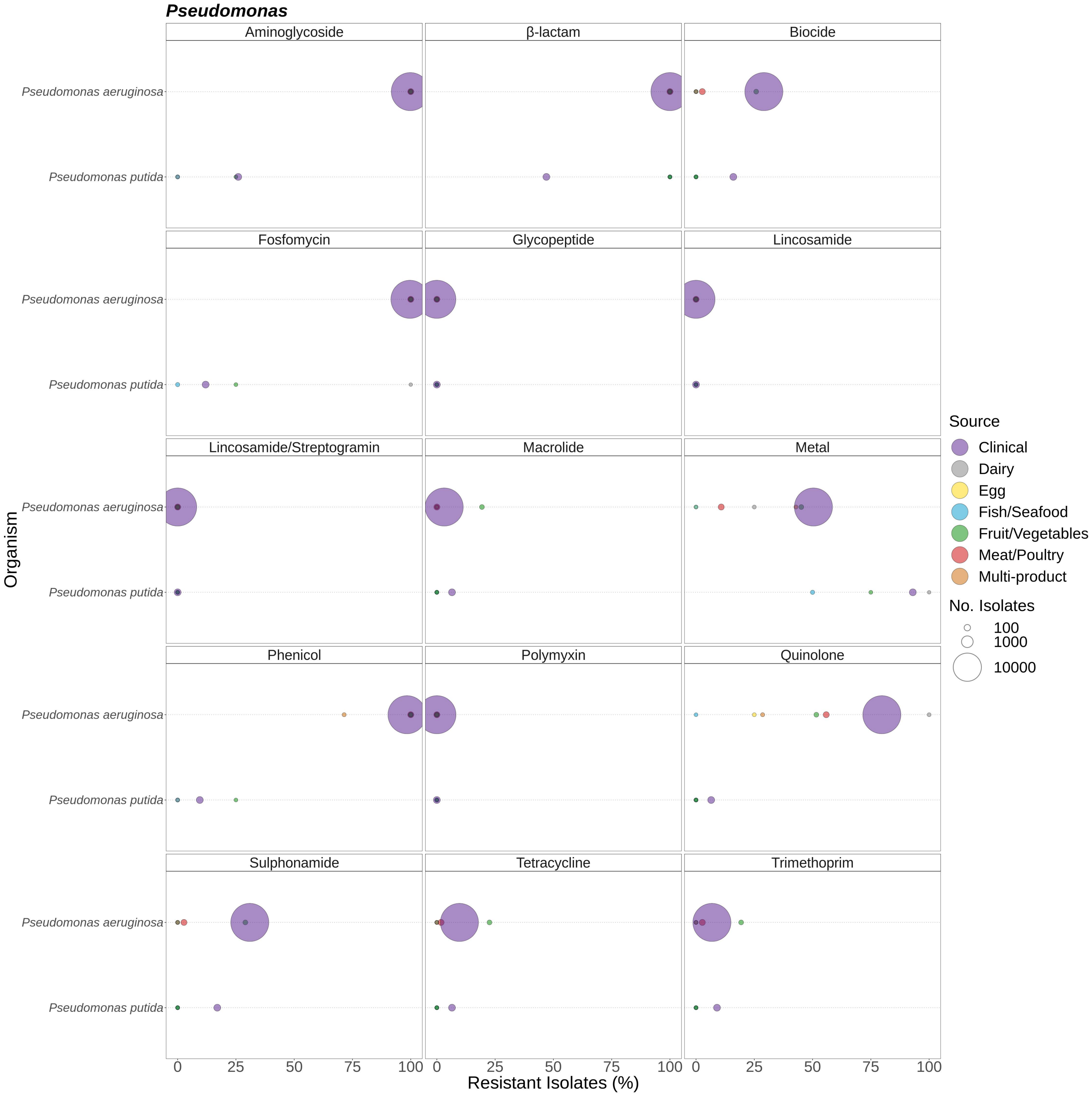
Pseudomonas
Aminoglycoside
β-lactam
Biocide
Fosfomycin
Glycopeptide
Lincosamide
Lincosamide/Streptogramin
Macrolide
Metal
Phenicol
Polymyxin
Quinolone
Sulphonamide
Tetracycline
Trimethoprim
Pseudomonas aeruginosa
Pseudomonas putida
Organism
0 25 50 75 100
Resistant Isolates (%)
Source
Clinical
Dairy
Egg
Fish/Seafood
Fruit/Vegetables
Meat/Poultry
Multi-product
No. Isolates
100
1000
10000

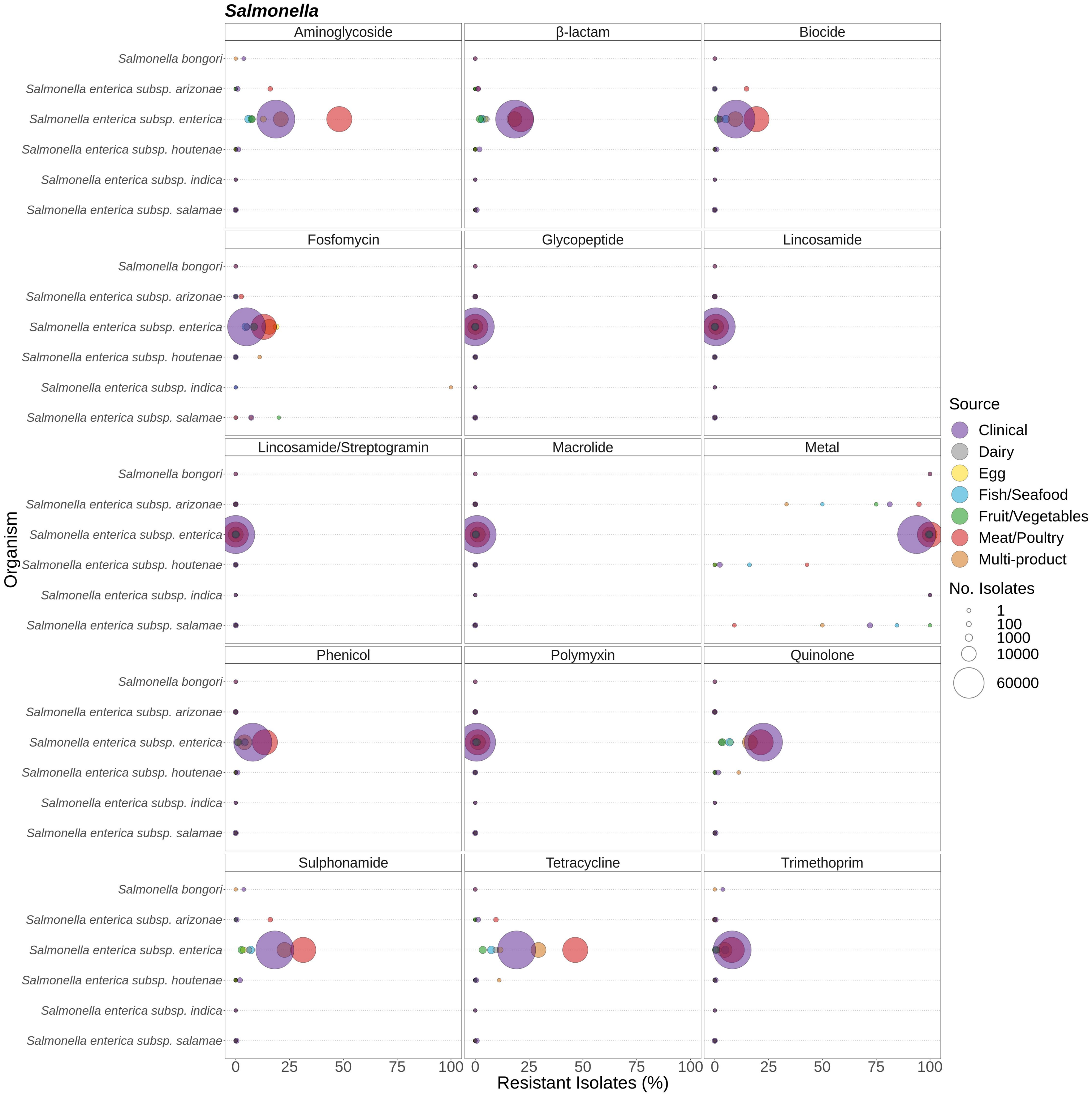
Salmonella
Aminoglycoside
β-lactam
Biocide
Fosfomycin
Glycopeptide
Lincosamide
Lincosamide/Streptogramin
Macrolide
Metal
Phenicol
Polymyxin
Quinolone
Sulphonamide
Tetracycline
Trimethoprim
Salmonella bongori
Salmonella enterica subsp. arizonae
Salmonella enterica subsp. enterica
Salmonella enterica subsp. houtenae
Salmonella enterica subsp. indica
Salmonella enterica subsp. salamae
Organism
0
25
50
75
100
Resistant Isolates (%)
Source
Clinical
Dairy
Egg
Fish/Seafood
Fruit/Vegetables
Meat/Poultry
Multi-product
No. Isolates
1
100
1000
10000
60000

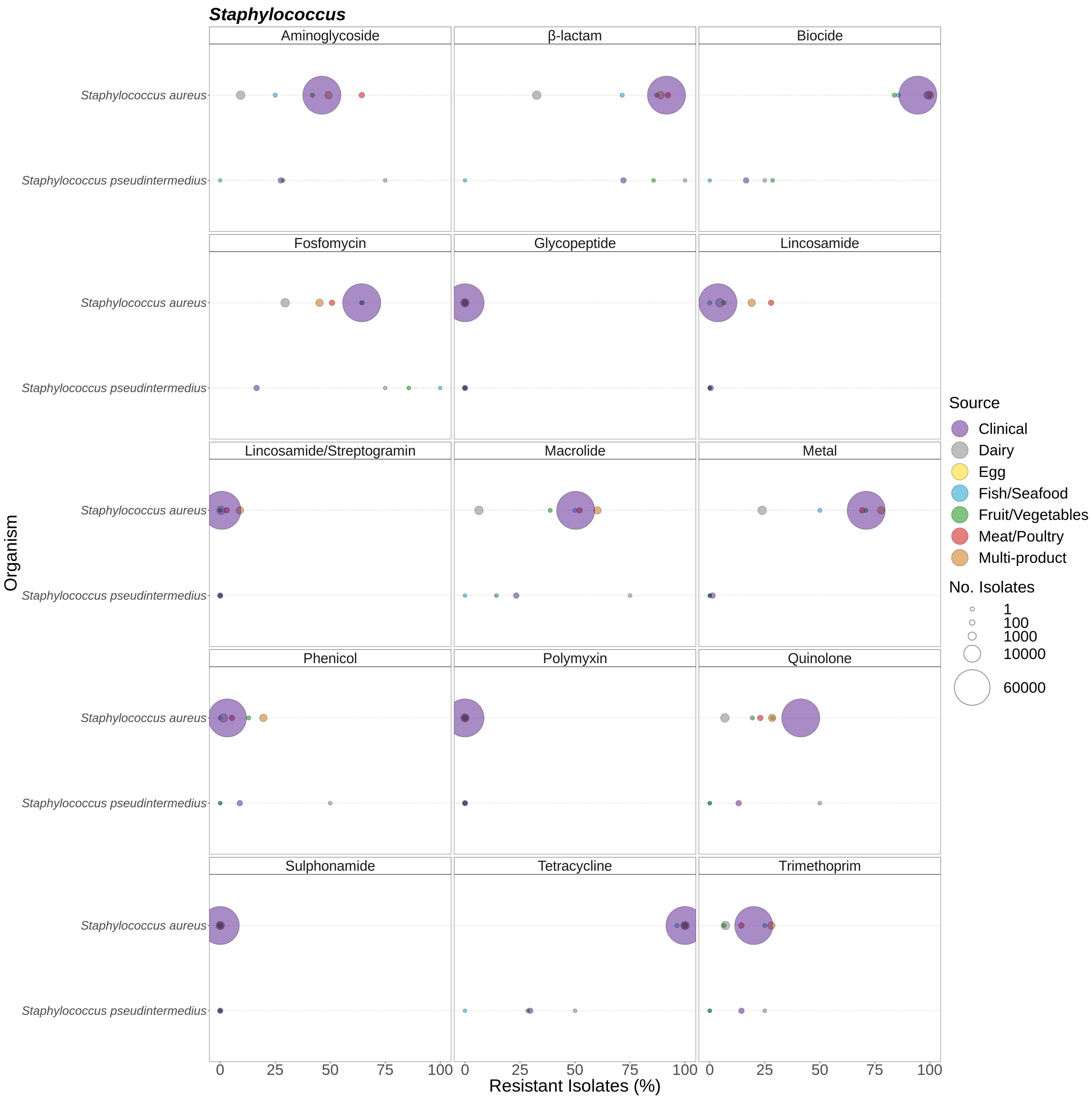
Staphylococcus
Aminoglycoside
β-lactam
Biocide
Fosfomycin
Glycopeptide
Lincosamide
Lincosamide/Streptogramin
Macrolide
Metal
Phenicol
Polymyxin
Quinolone
Sulphonamide
Tetracycline
Trimethoprim
Staphylococcus aureus
Staphylococcus pseudintermedius
Organism
Resistant Isolates (%)
0
25
50
75
100
Source
Clinical
Dairy
Egg
Fish/Seafood
Fruit/Vegetables
Meat/Poultry
Multi-product
No. Isolates
1
100
1000
10000
60000

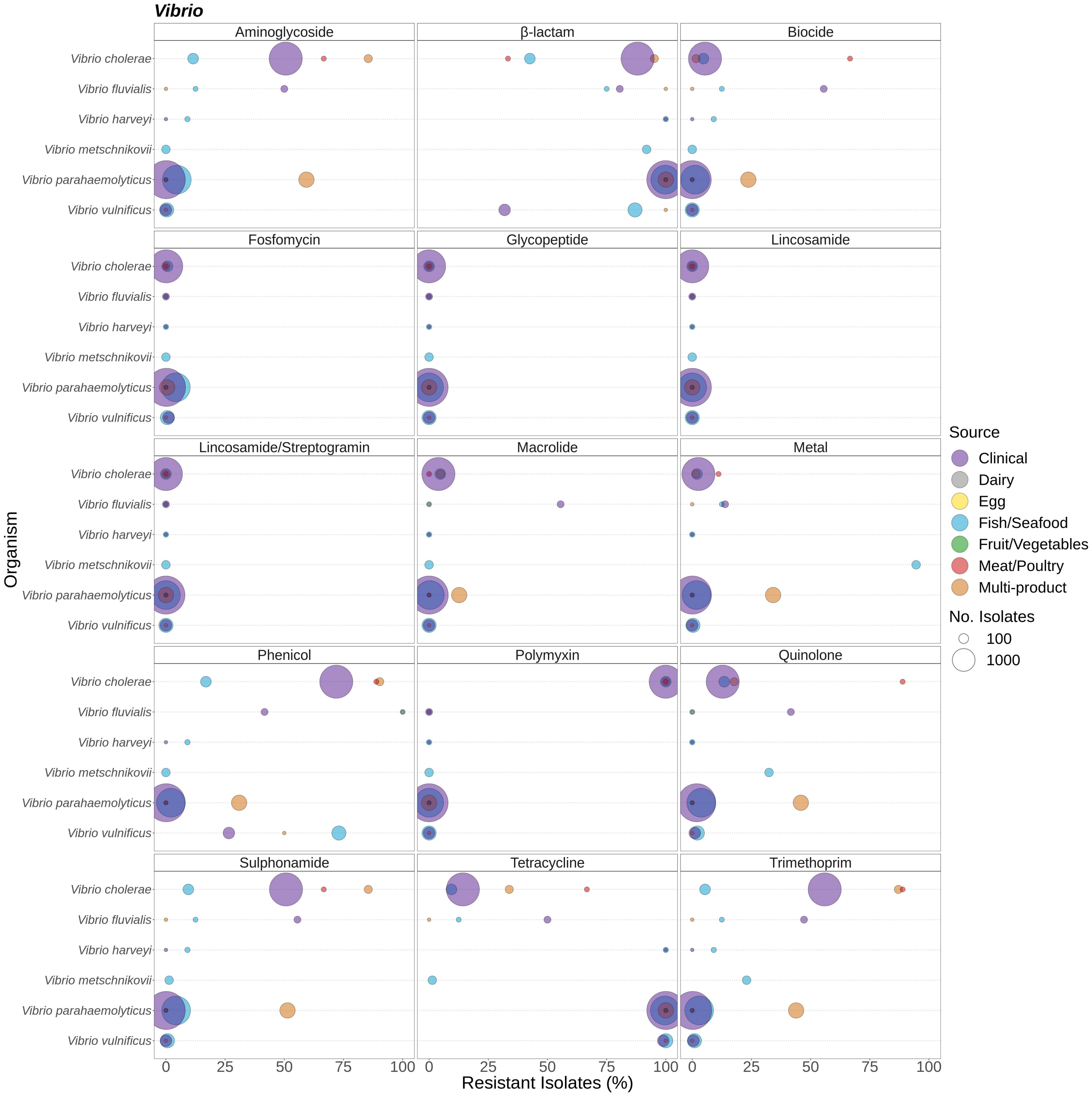
Vibrio
Aminoglycoside
β-lactam
Biocide
Fosfomycin
Glycopeptide
Lincosamide
Lincosamide/Streptogramin
Macrolide
Metal
Phenicol
Polymyxin
Quinolone
Sulphonamide
Tetracycline
Trimethoprim
Vibrio cholerae
Vibrio fluvialis
Vibrio harveyi
Vibrio metschnikovii
Vibrio parahaemolyticus
Vibrio vulnificus
Organism
0
25
50
75
100
Resistant Isolates (%)
Source
Clinical
Dairy
Egg
Fish/Seafood
Fruit/Vegetables
Meat/Poultry
Multi-product
No. Isolates
100
1000
