# Supplementary material for: Analysis of Antimicrobial Resistance in Bacterial Pathogens Recovered from Food and Human Sources: Insights from 639,087 Bacterial Whole-Genome Sequences in the NCBI Pathogen Detection Database
Source: Microorganisms. 2024 Mar 30;12(4):709. doi: 10.3390/microorganisms12040709 (PMC11051753; doi:10.3390/microorganisms12040709)
Supplement: Supplementary file 1 [file microorganisms-12-00709-s001.zip › Supplementary File S2.pdf]

Supplementary File S2

The following pages contain supplementary figures for statistical comparison of resistance between sources for each genus. (A) Heatmaps of statistical analysis comparing proportion of isolates encoding resistance between each isolation source (x- and y- axes) in plot B. Statistical comparison was conducted using pairwise Fisher's exact test fro proportions with Benjamini and Hochberg (BH) corrections. For each antimicrobial class (top-panel headings), significance between sources (heatmap axes) is denoted by fill (scale is log10 adjusted p-value) and within boxes: ns = not significant; $p < 0.05$ *; $p < 0.01$ = **; $p < 0.001$ = ***; $p < 0.0001$ = ****. Grey fill indicates very high significance, which is outside the range of the color gradient (varies by plot). (B) Bubbleplot indicates proportion of resistance (x-axis) for each resistance class (y-axis) from food product and clinical sources (differentiated by color). Sizing of points corresponds to the total number of isolates of genus for which isolates were downloaded from the NCBI Pathogen Detection database. Datasets were analysed and source categories were assigned based on reported sequence submission information. Data was excluded from analysis where source information was missing or incomplete. Quinolone class includes mobile genes (e.g. qnrS) and chromosomal point mutations (gyrA, parE, parC) reported for some genera.

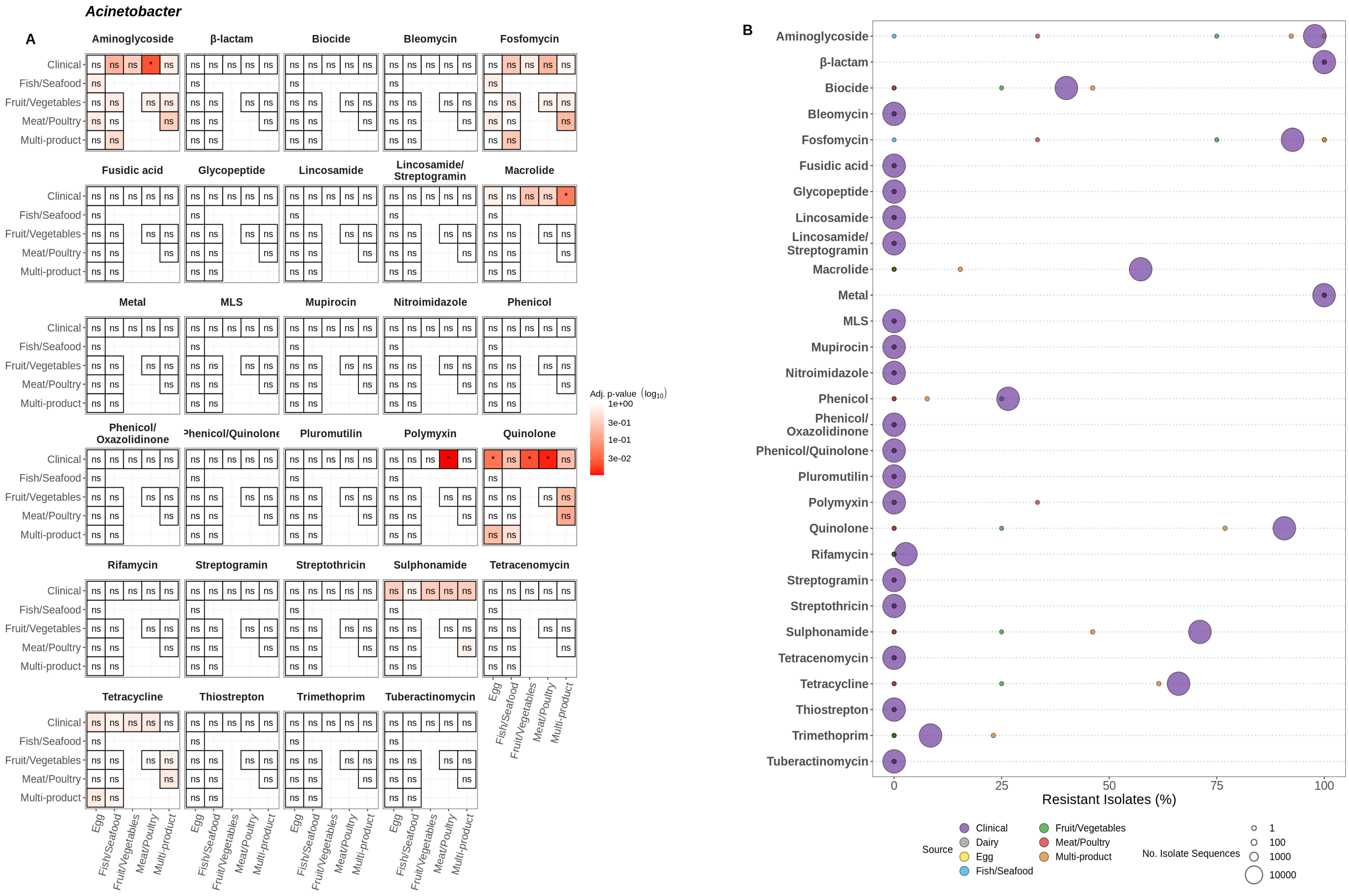
Acinetobacter
A
Aminoglycoside
β-lactam
Biocide
Bleomycin
Fosfomycin
Fusidic acid
Glycopeptide
Lincosamide
Lincosamide/ Streptogramin
Macrolide
Metal
MLS
Mupirocin
Nitroimidazole
Phenicol
Phenicol/ Oxazolidinone
Phenicol/Quinolone
Pluromutilin
Polymyxin
Quinolone
Rifamycin
Streptogramin
Streptothricin
Sulphonamide
Tetracenomycin
Tetracycline
Thiostrepton
Trimethoprim
Tuberactinomycin
Clinical
Fish/Seafood
Fruit/Vegetables
Meat/Poultry
Multi-product
Egg
Adj. p-value (log10)
1e+00
3e-01
1e-01
3e-02
B
Resistant Isolates (%)
0
25
50
75
100
Source
Clinical
Dairy
Egg
Fish/Seafood
Fruit/Vegetables
Meat/Poultry
Multi-product
No. Isolate Sequences
1
100
1000
10000

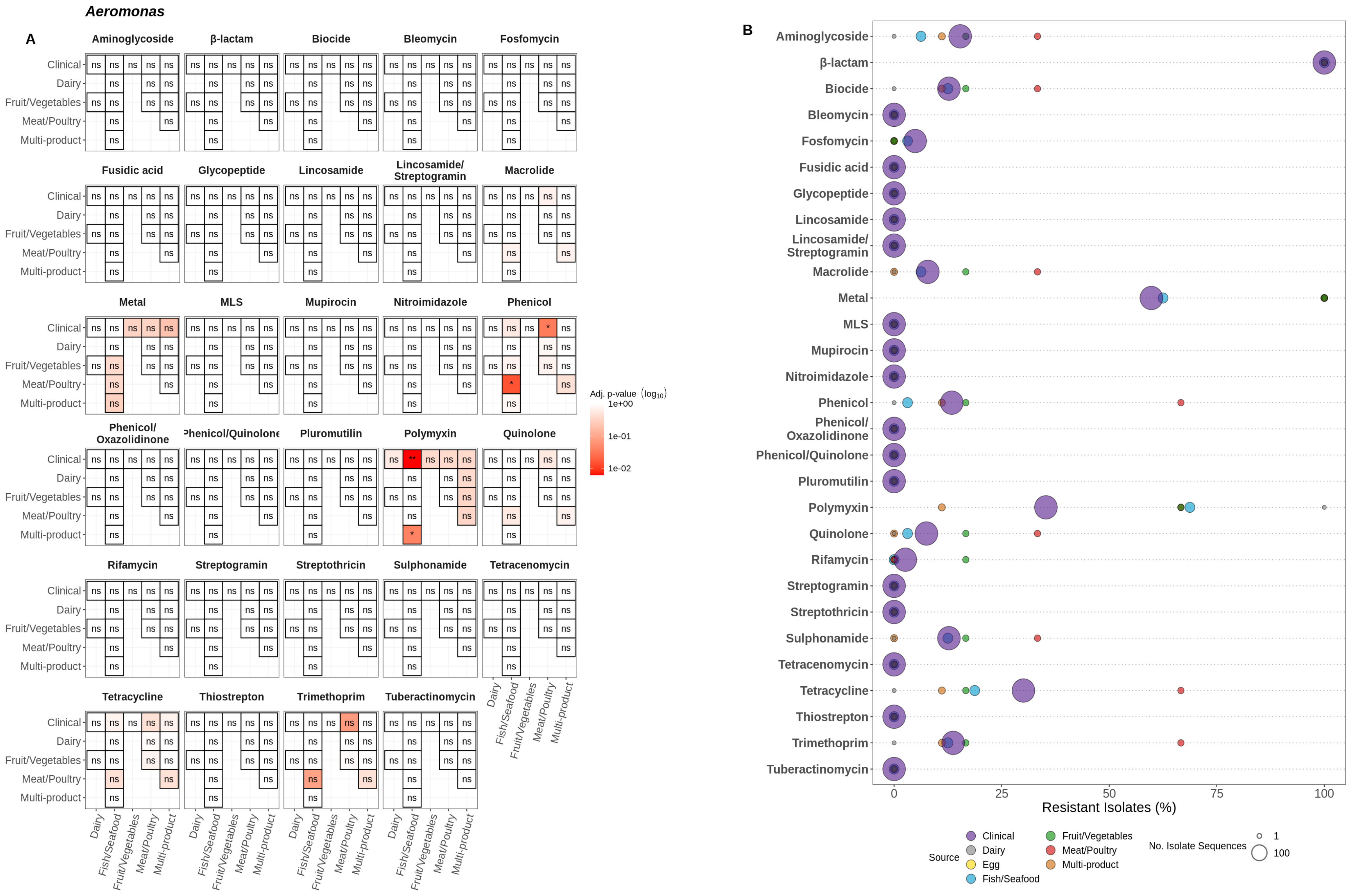

Aeromonas
A
Aminoglycoside
β-lactam
Biocide
Bleomycin
Fosfomycin
Fusidic acid
Glycopeptide
Lincosamide
Lincosamide/ Streptogramin
Macrolide
Metal
MLS
Mupirocin
Nitroimidazole
Phenicol
Phenicol/ Oxazolidinone
Phenicol/Quinolone
Pluromutilin
Polymyxin
Quinolone
Rifamycin
Streptogramin
Streptothricin
Sulphonamide
Tetracenomycin
Tetracycline
Thiostrepton
Trimethoprim
Tuberactinomycin
Clinical
Dairy
Fruit/Vegetables
Meat/Poultry
Multi-product
Fish/Seafood
Adj. p-value (log10)
1e+00
1e-01
1e-02
B
Resistant Isolates (%)
0
25
50
75
100
Source
Clinical
Dairy
Egg
Fish/Seafood
Fruit/Vegetables
Meat/Poultry
Multi-product
No. Isolate Sequences
1
100

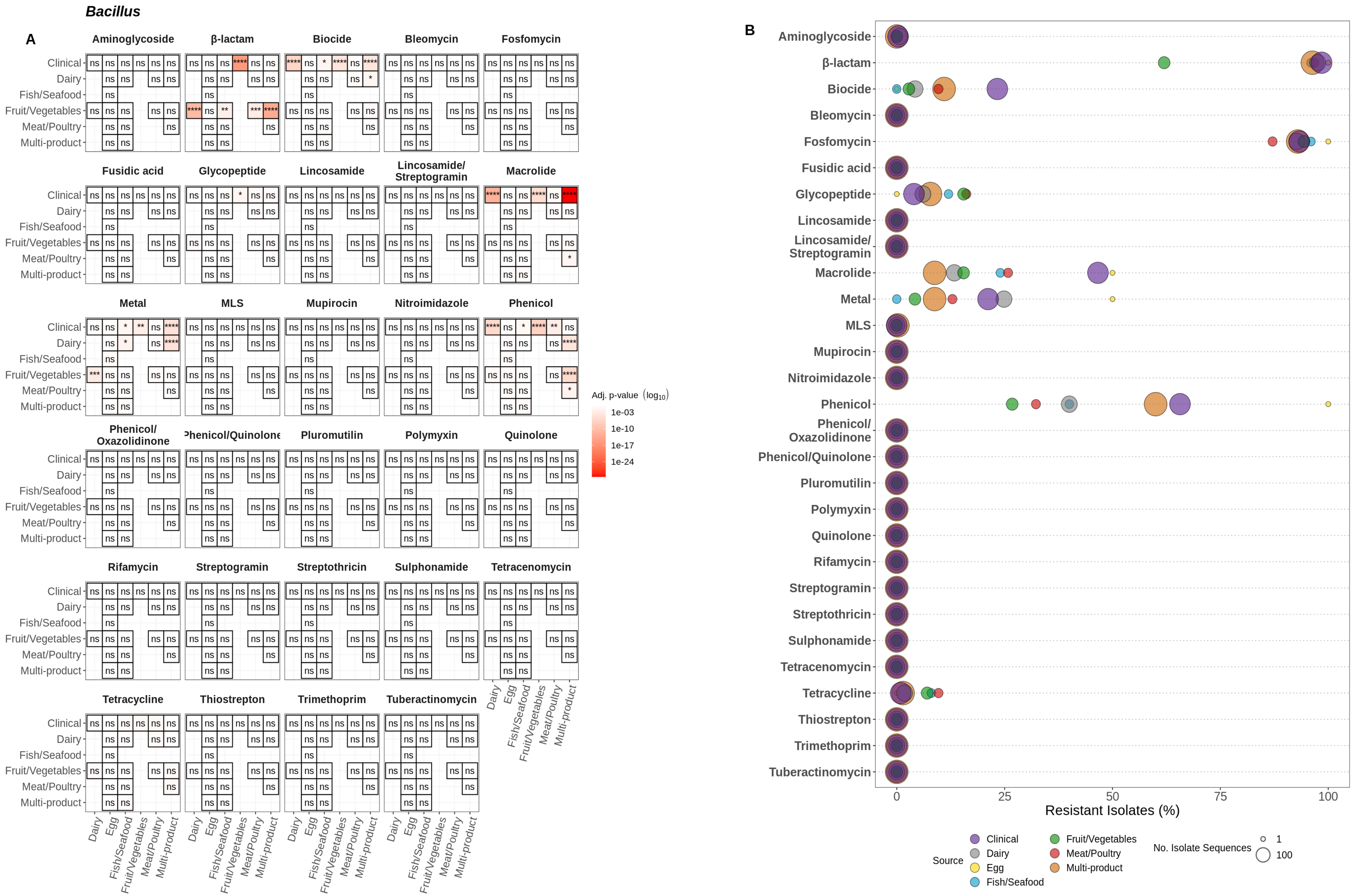
Bacillus
A
Aminoglycoside
β-lactam
Biocide
Bleomycin
Fosfomycin
Fusidic acid
Glycopeptide
Lincosamide
Lincosamide/ Streptogramin
Macrolide
Metal
MLS
Mupirocin
Nitroimidazole
Phenicol
Phenicol/ Oxazolidinone
Phenicol/Quinolone
Pluromutilin
Polymyxin
Quinolone
Rifamycin
Streptogramin
Streptothricin
Sulphonamide
Tetracenomycin
Tetracycline
Thiostrepton
Trimethoprim
Tuberactinomycin
Clinical
Dairy
Fish/Seafood
Fruit/Vegetables
Meat/Poultry
Multi-product
Egg
Adj. p-value (log10)
1e-03
1e-10
1e-17
1e-24
B
Resistant Isolates (%)
0
25
50
75
100
Source
No. Isolate Sequences
1
100

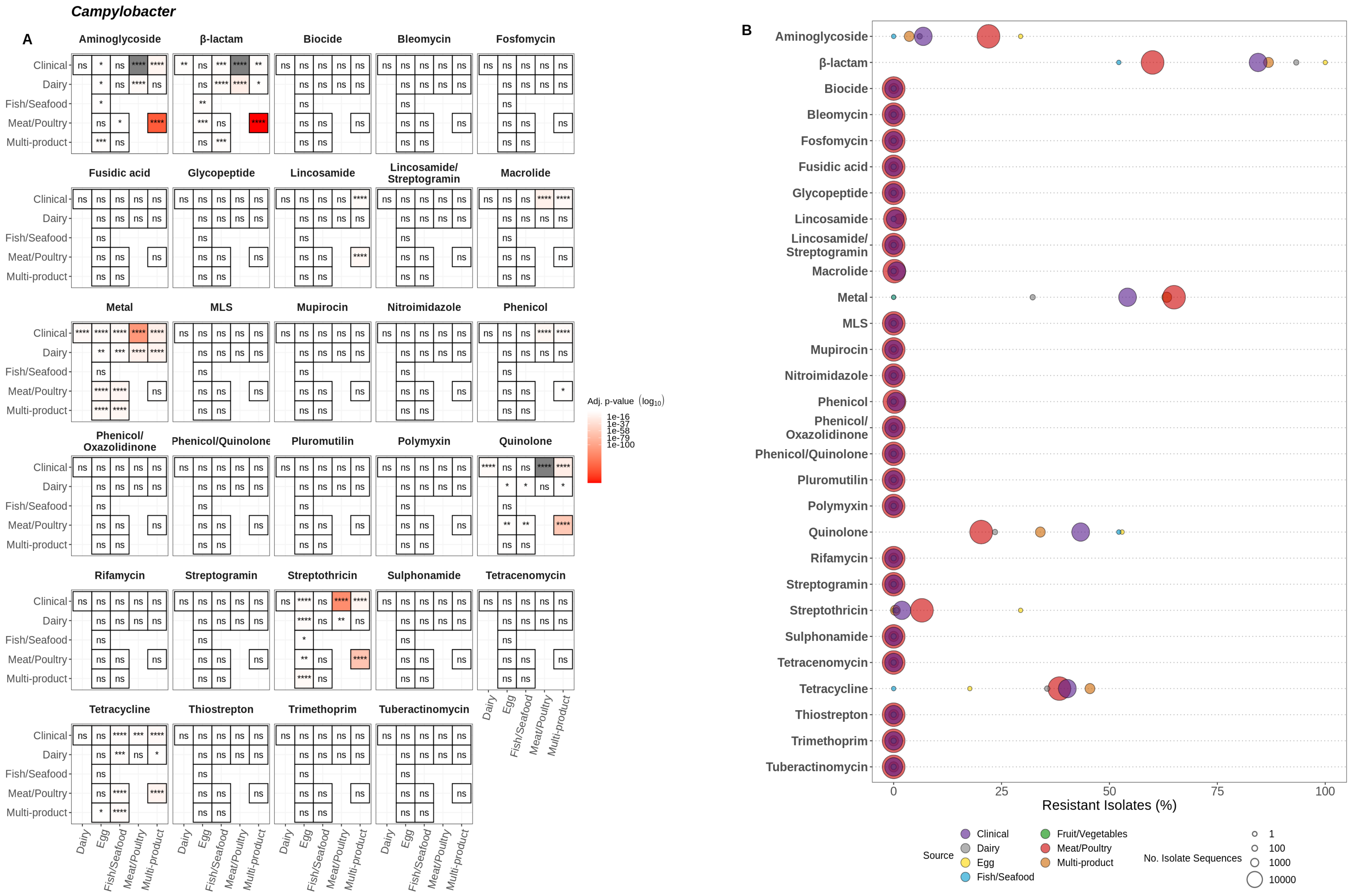
Campylobacter
A
Aminoglycoside
β-lactam
Biocide
Bleomycin
Fosfomycin
Fusidic acid
Glycopeptide
Lincosamide
Lincosamide/ Streptogramin
Macrolide
Metal
MLS
Mupirocin
Nitroimidazole
Phenicol
Phenicol/ Oxazolidinone
Phenicol/Quinolone
Pluromutilin
Polymyxin
Quinolone
Rifamycin
Streptogramin
Streptothricin
Sulphonamide
Tetracenomycin
Tetracycline
Thiostrepton
Trimethoprim
Tuberactinomycin
Clinical
Dairy
Fish/Seafood
Meat/Poultry
Multi-product
Dairy
Egg
Fish/Seafood
Meat/Poultry
Multi-product
Adj. p-value (log10)
1e-16
1e-37
1e-58
1e-79
1e-100
B
Resistant Isolates (%)
0
25
50
75
100
Source
Clinical
Dairy
Egg
Fish/Seafood
Fruit/Vegetables
Meat/Poultry
Multi-product
No. Isolate Sequences
1
100
1000
10000

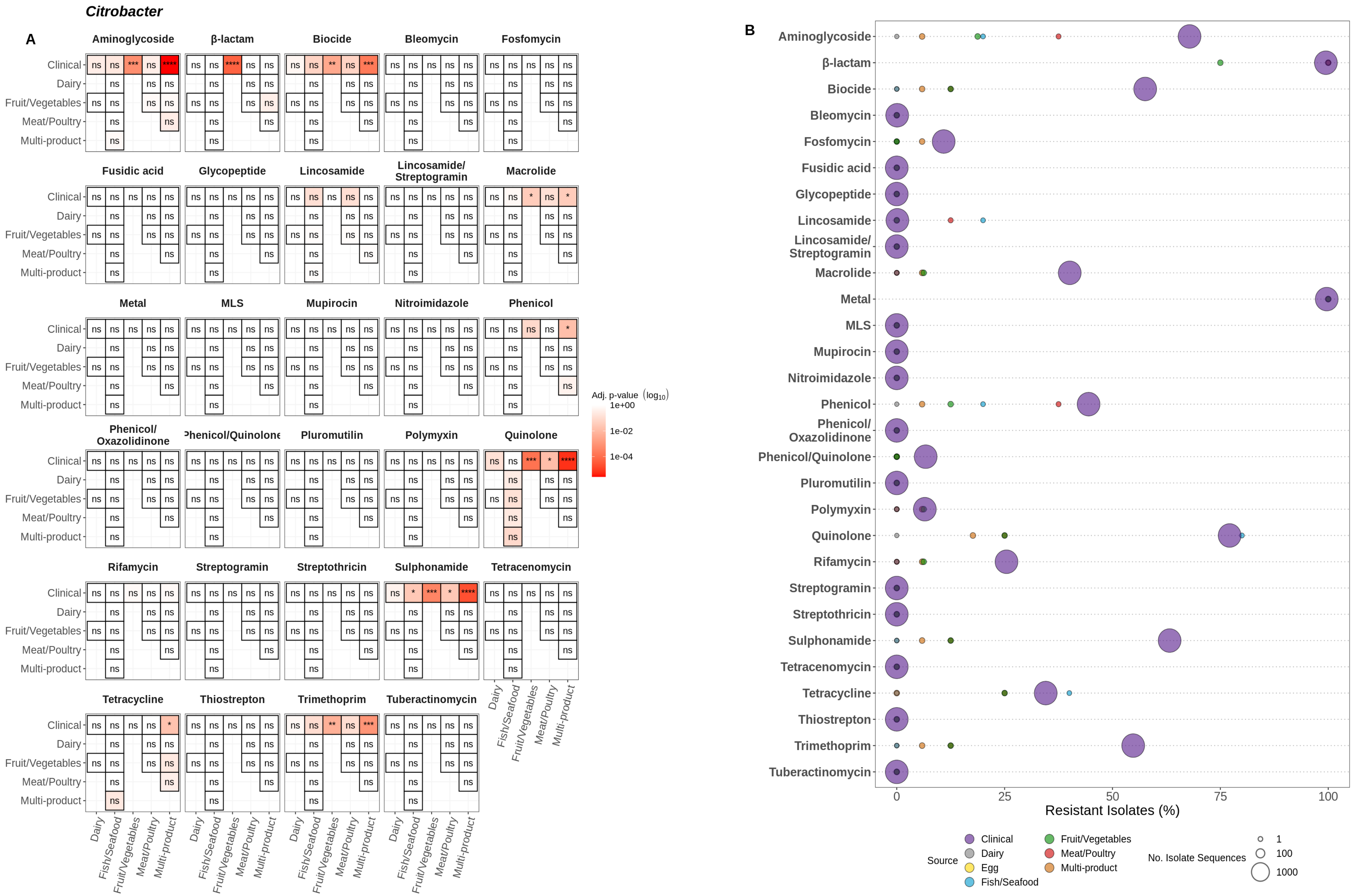
Citrobacter
A
Aminoglycoside
β-lactam
Biocide
Bleomycin
Fosfomycin
Fusidic acid
Glycopeptide
Lincosamide
Lincosamide/ Streptogramin
Macrolide
Metal
MLS
Mupirocin
Nitroimidazole
Phenicol
Phenicol/ Oxazolidinone
Phenicol/Quinolone
Pluromutilin
Polymyxin
Quinolone
Rifamycin
Streptogramin
Streptothricin
Sulphonamide
Tetracenomycin
Tetracycline
Thiostrepton
Trimethoprim
Tuberactinomycin
Clinical
Dairy
Fruit/Vegetables
Meat/Poultry
Multi-product
Dairy
Fish/Seafood
Fruit/Vegetables
Meat/Poultry
Multi-product
Adj. p-value (log10)
1e+00
1e-02
1e-04
B
0
25
50
75
100
Resistant Isolates (%)
Source
Clinical
Dairy
Egg
Fish/Seafood
Fruit/Vegetables
Meat/Poultry
Multi-product
No. Isolate Sequences
1
100
1000

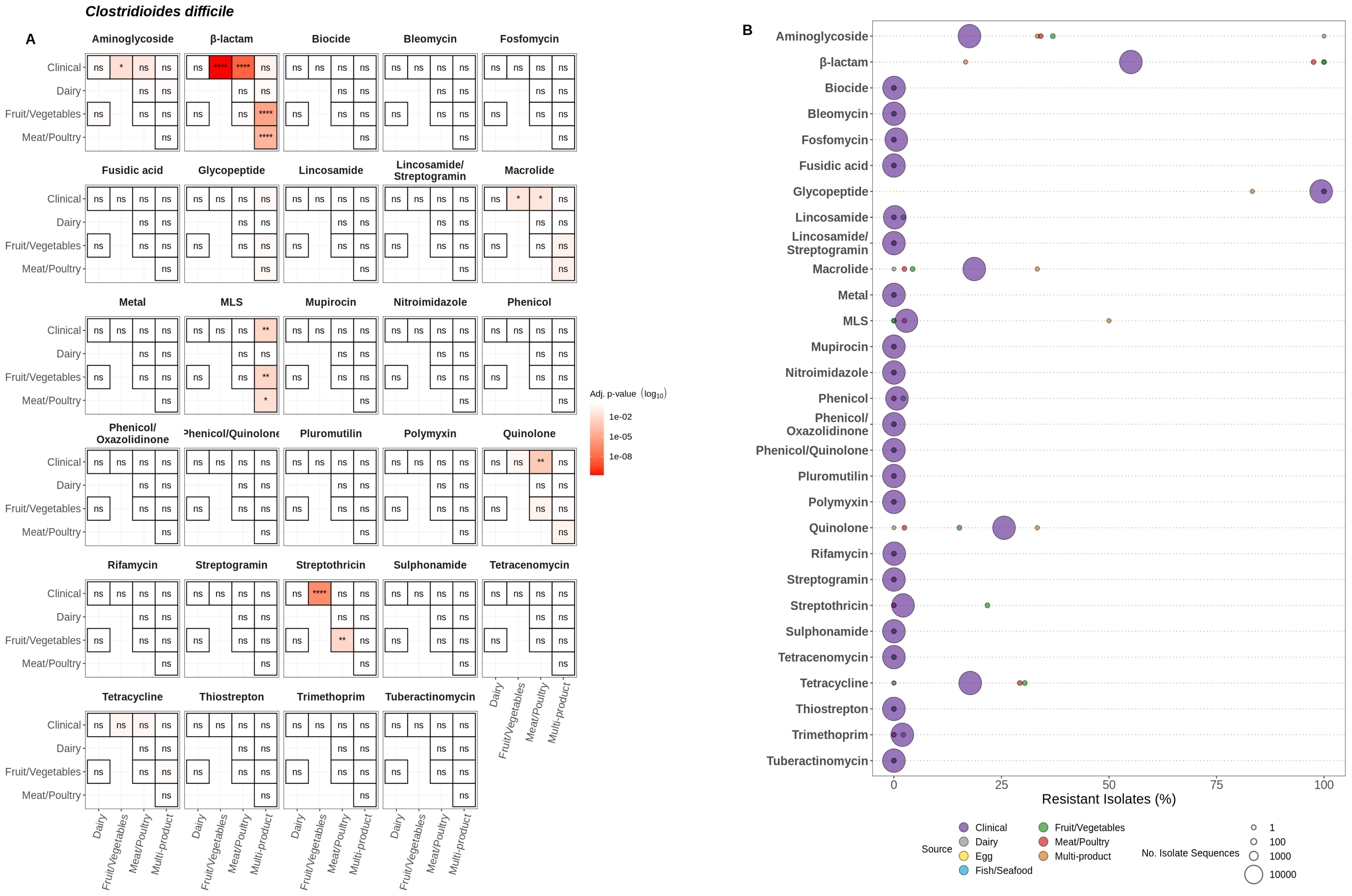
Clostridioides difficile
A
B
Aminoglycoside
β-lactam
Biocide
Bleomycin
Fosfomycin
Fusidic acid
Glycopeptide
Lincosamide
Lincosamide/ Streptogramin
Macrolide
Metal
MLS
Mupirocin
Nitroimidazole
Phenicol
Phenicol/ Oxazolidinone
Phenicol/Quinolone
Pluromutilin
Polymyxin
Quinolone
Rifamycin
Streptogramin
Streptothricin
Sulphonamide
Tetracenomycin
Tetracycline
Thiostrepton
Trimethoprim
Tuberactinomycin
Clinical
Dairy
Fruit/Vegetables
Meat/Poultry
Multi-product
Adj. p-value (log10)
1e-02
1e-05
1e-08
Resistant Isolates (%)
0
25
50
75
100
Source
Clinical
Dairy
Egg
Fish/Seafood
Fruit/Vegetables
Meat/Poultry
Multi-product
No. Isolate Sequences
1
100
1000
10000

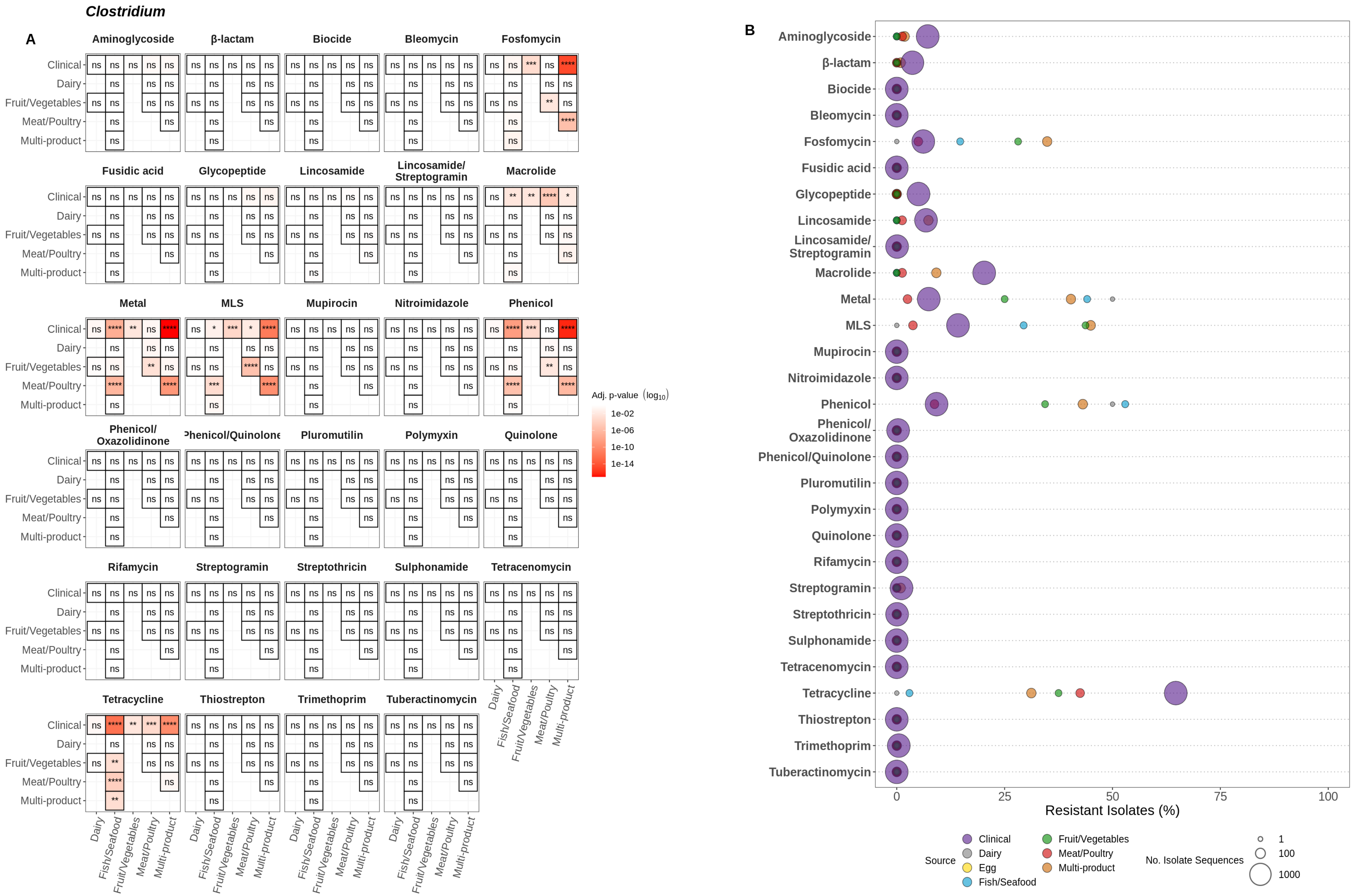
Clostridium
A
Aminoglycoside
β-lactam
Biocide
Bleomycin
Fosfomycin
Fusidic acid
Glycopeptide
Lincosamide
Lincosamide/ Streptogramin
Macrolide
Metal
MLS
Mupirocin
Nitroimidazole
Phenicol
Phenicol/ Oxazolidinone
Phenicol/Quinolone
Pluromutilin
Polymyxin
Quinolone
Rifamycin
Streptogramin
Streptothricin
Sulphonamide
Tetracenomycin
Tetracycline
Thiostrepton
Trimethoprim
Tuberactinomycin
Clinical
Dairy
Fruit/Vegetables
Meat/Poultry
Multi-product
Fish/Seafood
Adj. p-value (log10)
1e-02
1e-06
1e-10
1e-14
B
0
25
50
75
100
Resistant Isolates (%)
Source
Clinical
Dairy
Egg
Fish/Seafood
Fruit/Vegetables
Meat/Poultry
Multi-product
No. Isolate Sequences
1
100
1000

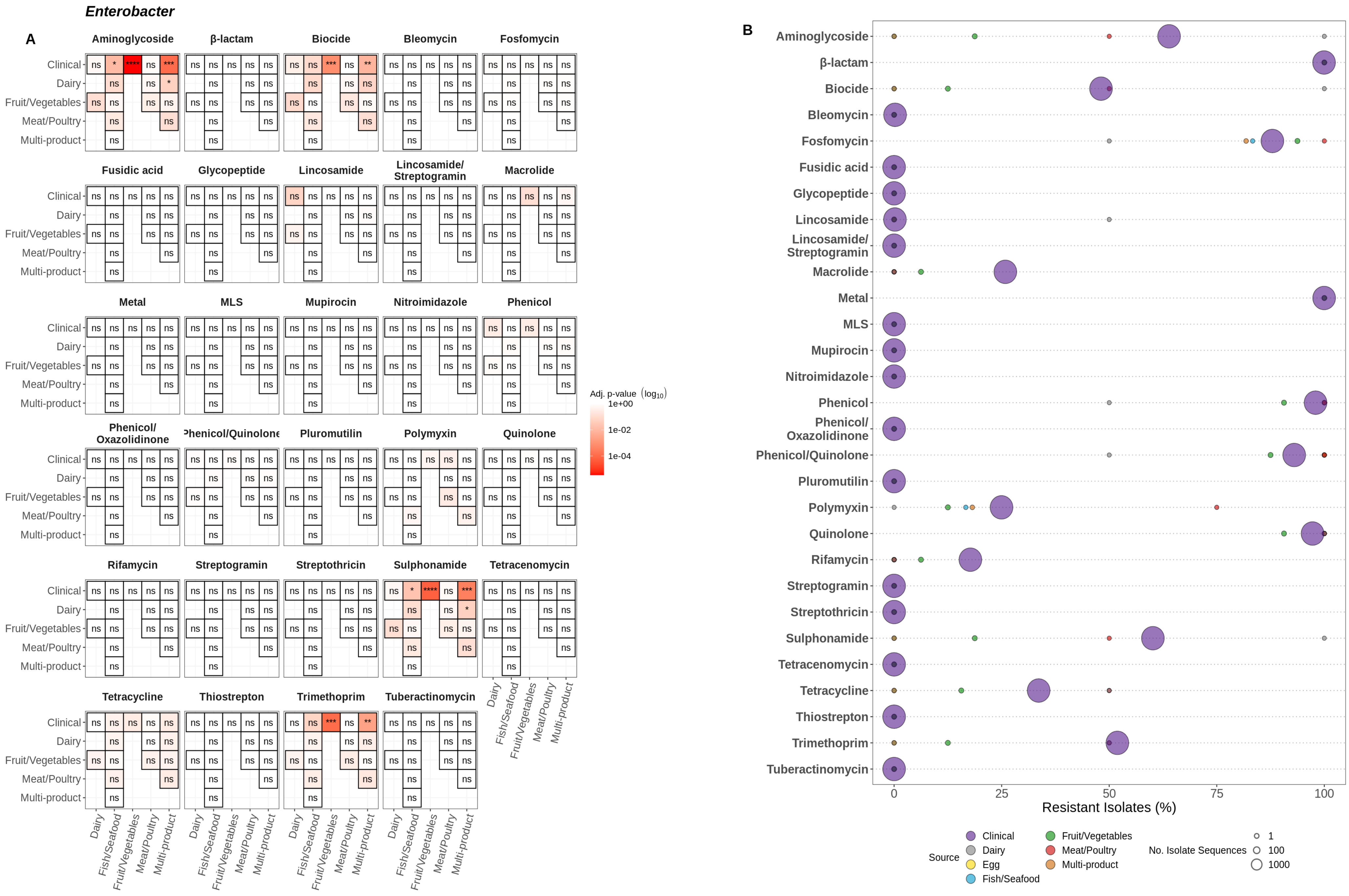
Enterobacter
A
B
Adj. p-value (log10)
Resistant Isolates (%)
Source
Clinical
Dairy
Egg
Fish/Seafood
Fruit/Vegetables
Meat/Poultry
Multi-product
No. Isolate Sequences

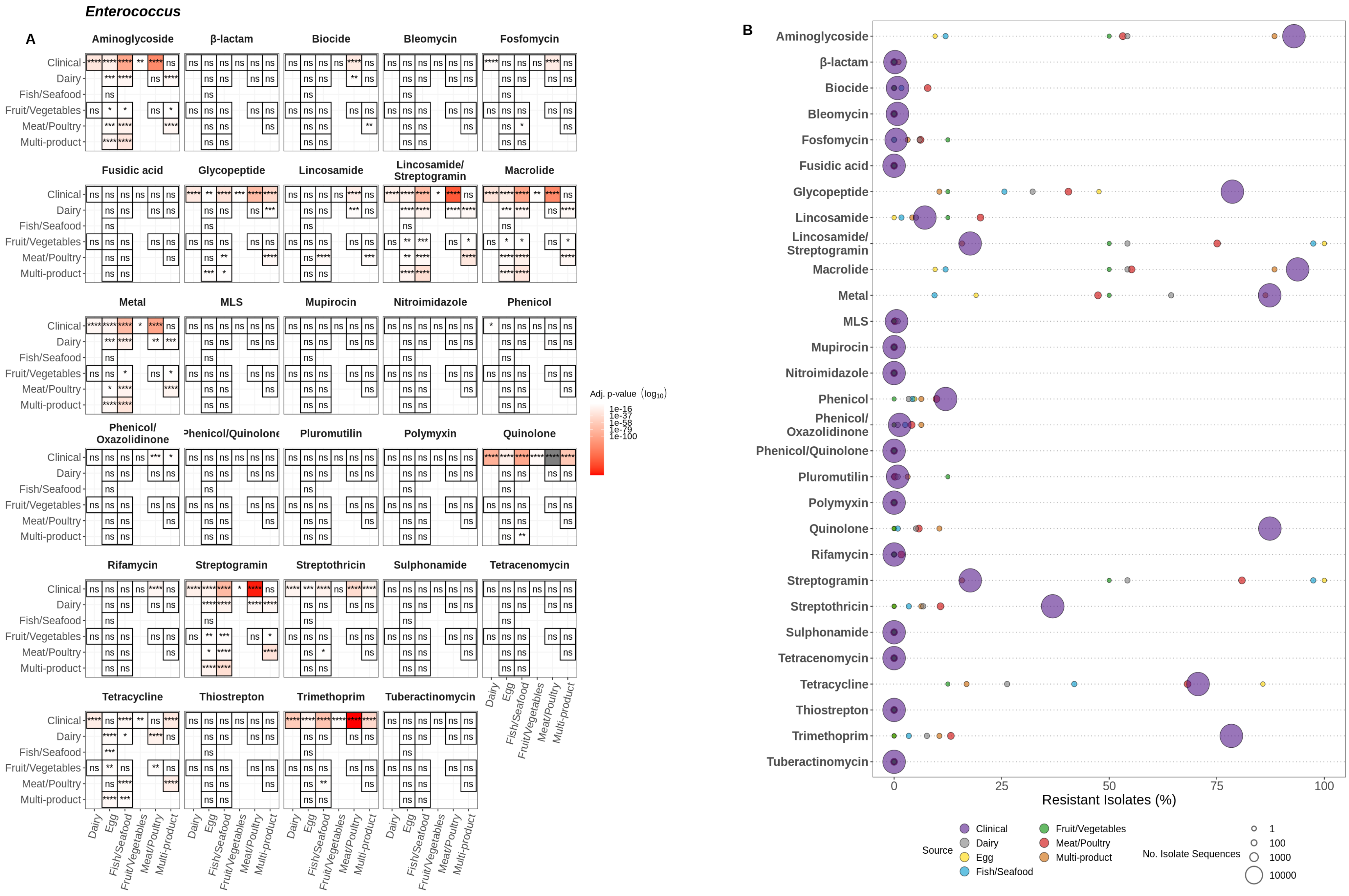
Enterococcus
A
Aminoglycoside
β-lactam
Biocide
Bleomycin
Fosfomycin
Fusidic acid
Glycopeptide
Lincosamide
Lincosamide/ Streptogramin
Macrolide
Metal
MLS
Mupirocin
Nitroimidazole
Phenicol
Phenicol/ Oxazolidinone
Phenicol/Quinolone
Pluromutilin
Polymyxin
Quinolone
Rifamycin
Streptogramin
Streptothricin
Sulphonamide
Tetracenomycin
Tetracycline
Thiostrepton
Trimethoprim
Tuberactinomycin
Clinical
Dairy
Fish/Seafood
Fruit/Vegetables
Meat/Poultry
Multi-product
Dairy
Egg
Fish/Seafood
Fruit/Vegetables
Meat/Poultry
Multi-product
Adj. p-value (log10)
1e-16
1e-37
1e-58
1e-79
1e-100
B
Resistant Isolates (%)
0
25
50
75
100
Source
Clinical
Dairy
Egg
Fish/Seafood
Fruit/Vegetables
Meat/Poultry
Multi-product
No. Isolate Sequences
1
100
1000
10000

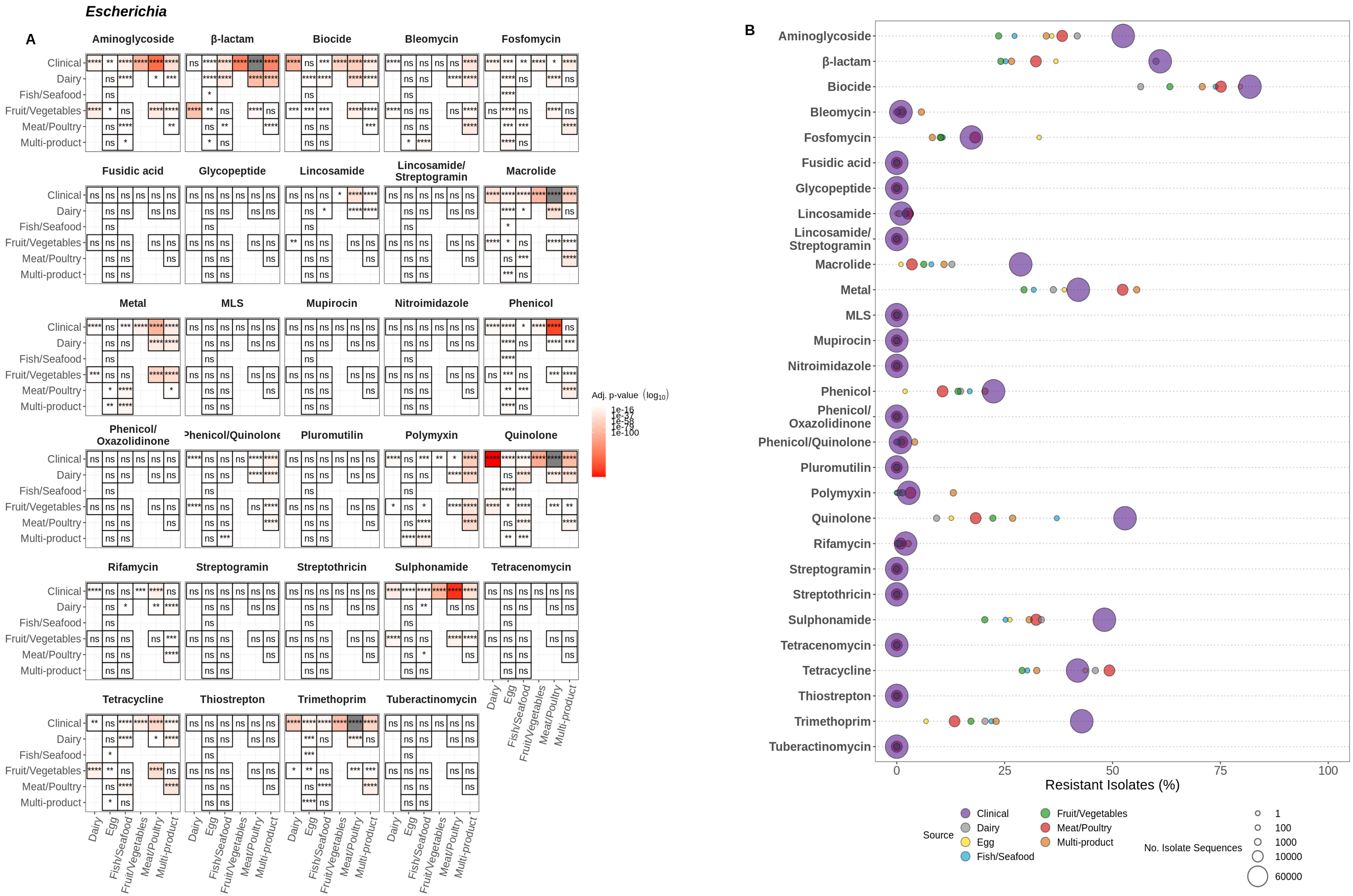
Escherichia
A
Aminoglycoside
β-lactam
Biocide
Bleomycin
Fosfomycin
Fusidic acid
Glycopeptide
Lincosamide
Lincosamide/ Streptogramin
Macrolide
Metal
MLS
Mupirocin
Nitroimidazole
Phenicol
Phenicol/ Oxazolidinone
Phenicol/Quinolone
Pluromutilin
Polymyxin
Quinolone
Rifamycin
Streptogramin
Streptothricin
Sulphonamide
Tetracenomycin
Tetracycline
Thiostrepton
Trimethoprim
Tuberactinomycin
Clinical
Dairy
Fish/Seafood
Fruit/Vegetables
Meat/Poultry
Multi-product
Dairy
Egg
Fish/Seafood
Fruit/Vegetables
Meat/Poultry
Multi-product
Adj. p-value (log10)
1e-16
1e-37
1e-58
1e-79
1e-100
B
Resistant Isolates (%)
0
25
50
75
100
Source
Clinical
Dairy
Egg
Fish/Seafood
Fruit/Vegetables
Meat/Poultry
Multi-product
No. Isolate Sequences
1
100
1000
10000
60000

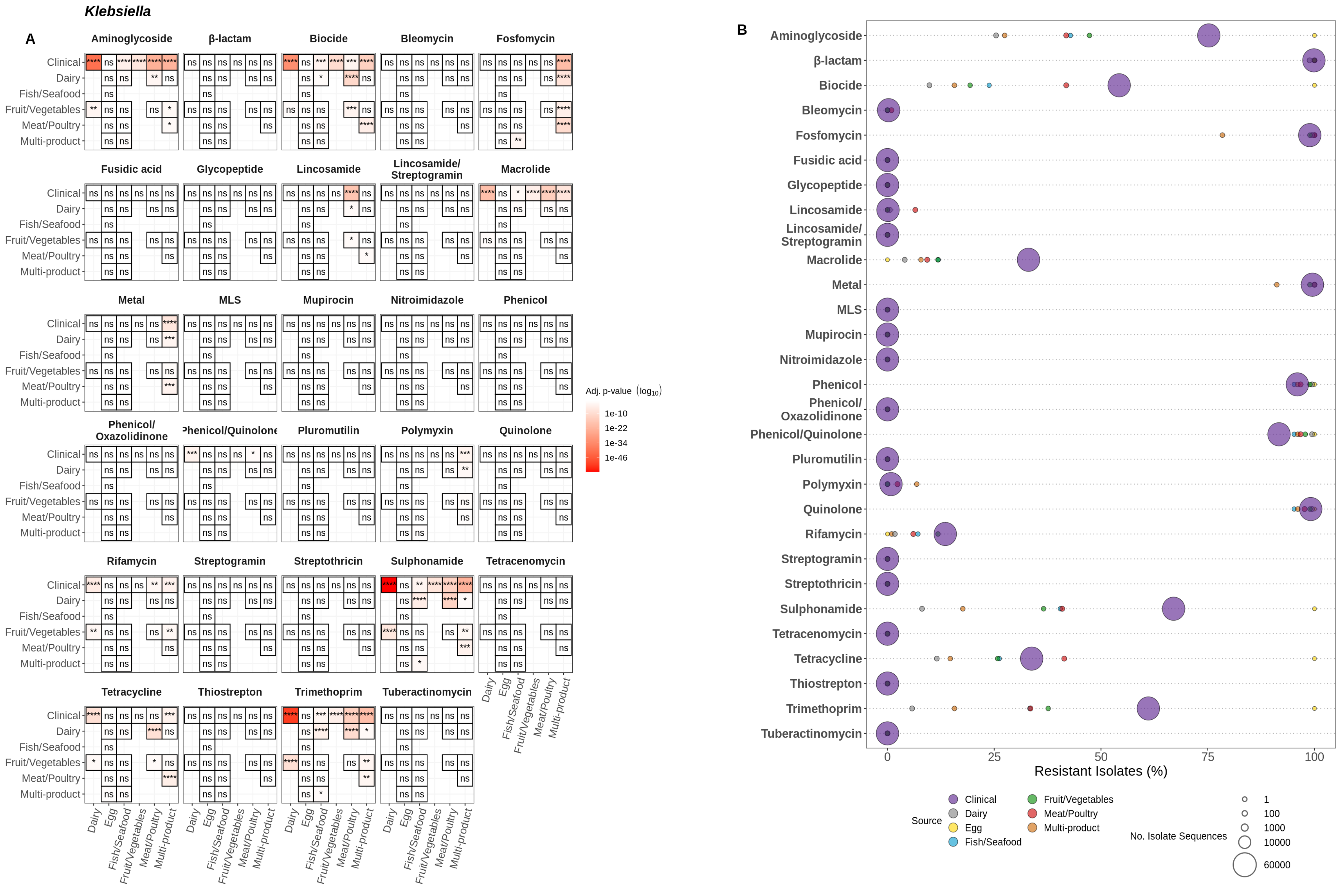
Klebsiella
A
Aminoglycoside
β-lactam
Biocide
Bleomycin
Fosfomycin
Fusidic acid
Glycopeptide
Lincosamide
Lincosamide/ Streptogramin
Macrolide
Metal
MLS
Mupirocin
Nitroimidazole
Phenicol
Phenicol/ Oxazolidinone
Phenicol/Quinolone
Pluromutilin
Polymyxin
Quinolone
Rifamycin
Streptogramin
Streptothricin
Sulphonamide
Tetracenomycin
Tetracycline
Thiostrepton
Trimethoprim
Tuberactinomycin
Clinical
Dairy
Fish/Seafood
Fruit/Vegetables
Meat/Poultry
Multi-product
Egg
Adj. p-value (log10)
1e-10
1e-22
1e-34
1e-46
B
0
25
50
75
100
Resistant Isolates (%)
Source
Clinical
Dairy
Egg
Fish/Seafood
Fruit/Vegetables
Meat/Poultry
Multi-product
No. Isolate Sequences
1
100
1000
10000
60000

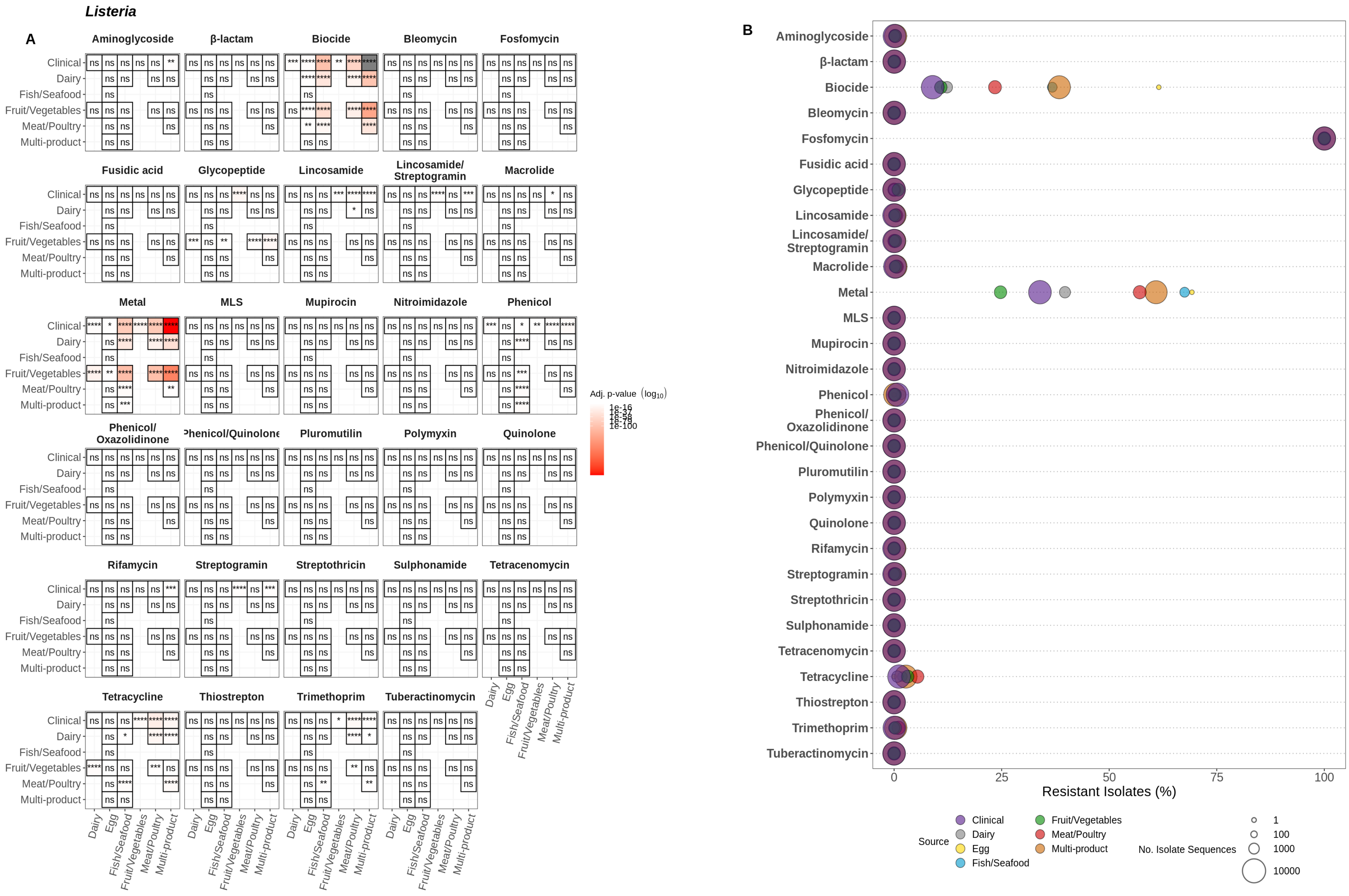
Listeria
A
Aminoglycoside
β-lactam
Biocide
Bleomycin
Fosfomycin
Fusidic acid
Glycopeptide
Lincosamide
Lincosamide/ Streptogramin
Macrolide
Metal
MLS
Mupirocin
Nitroimidazole
Phenicol
Phenicol/ Oxazolidinone
Phenicol/Quinolone
Pluromutilin
Polymyxin
Quinolone
Rifamycin
Streptogramin
Streptothricin
Sulphonamide
Tetracenomycin
Tetracycline
Thiostrepton
Trimethoprim
Tuberactinomycin
Clinical
Dairy
Fish/Seafood
Fruit/Vegetables
Meat/Poultry
Multi-product
Dairy
Egg
Fish/Seafood
Fruit/Vegetables
Meat/Poultry
Multi-product
Adj. p-value (log10)
1e-16
1e-100
B
Resistant Isolates (%)
0
25
50
75
100
Source
Clinical
Dairy
Egg
Fish/Seafood
Fruit/Vegetables
Meat/Poultry
Multi-product
No. Isolate Sequences
1
100
1000
10000

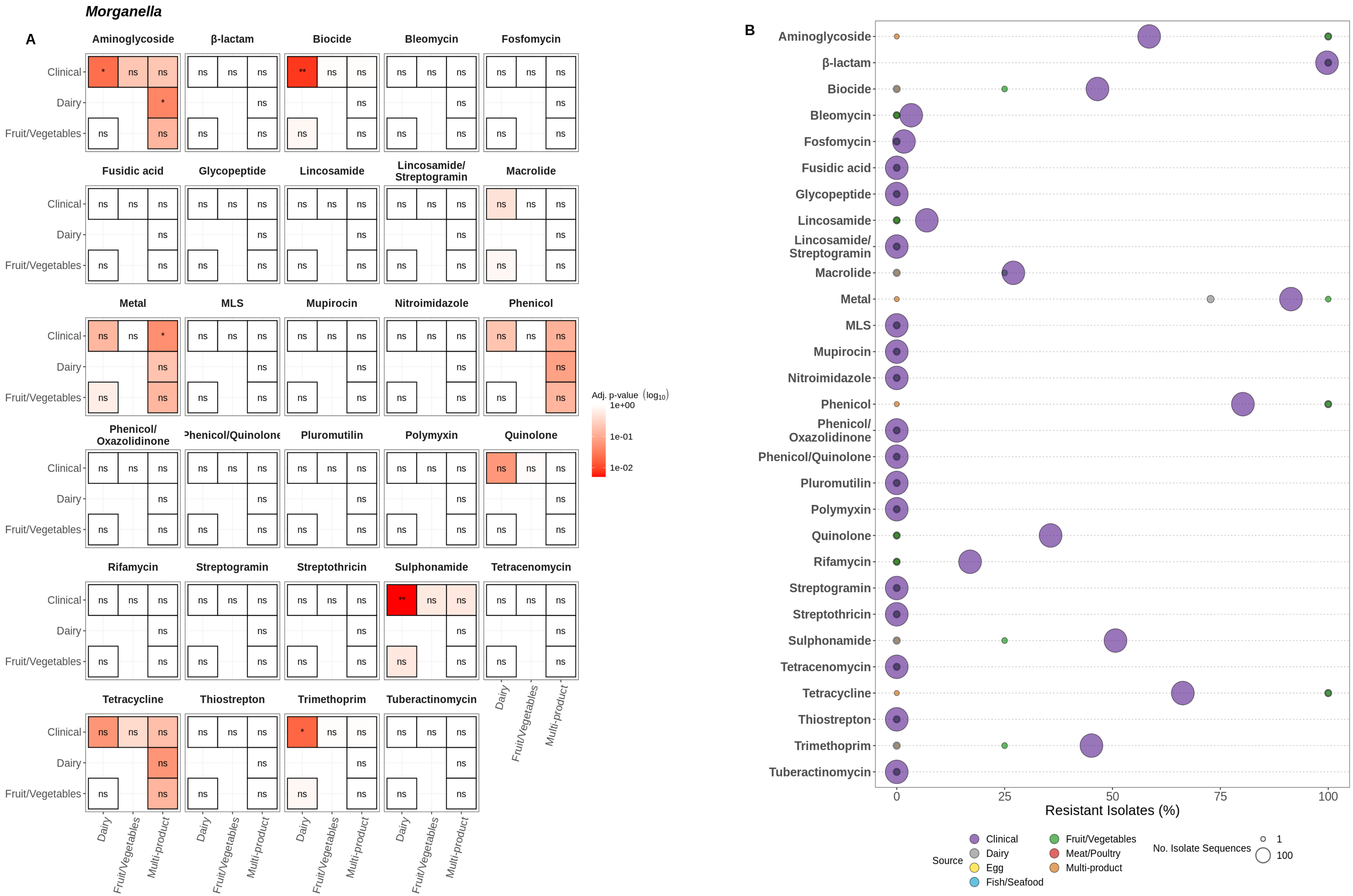
Morganella
A
Aminoglycoside
β-lactam
Biocide
Bleomycin
Fosfomycin
Fusidic acid
Glycopeptide
Lincosamide
Lincosamide/ Streptogramin
Macrolide
Metal
MLS
Mupirocin
Nitroimidazole
Phenicol
Phenicol/ Oxazolidinone
Phenicol/Quinolone
Pluromutilin
Polymyxin
Quinolone
Rifamycin
Streptogramin
Streptothricin
Sulphonamide
Tetracenomycin
Tetracycline
Thiostrepton
Trimethoprim
Tuberactinomycin
Clinical
Dairy
Fruit/Vegetables
Multi-product
ns
*
**
Adj. p-value (log_{10})
1e+00
1e-01
1e-02
B
0
25
50
75
100
Resistant Isolates (%)
Source
Clinical
Dairy
Egg
Fish/Seafood
Fruit/Vegetables
Meat/Poultry
Multi-product
No. Isolate Sequences
1
100

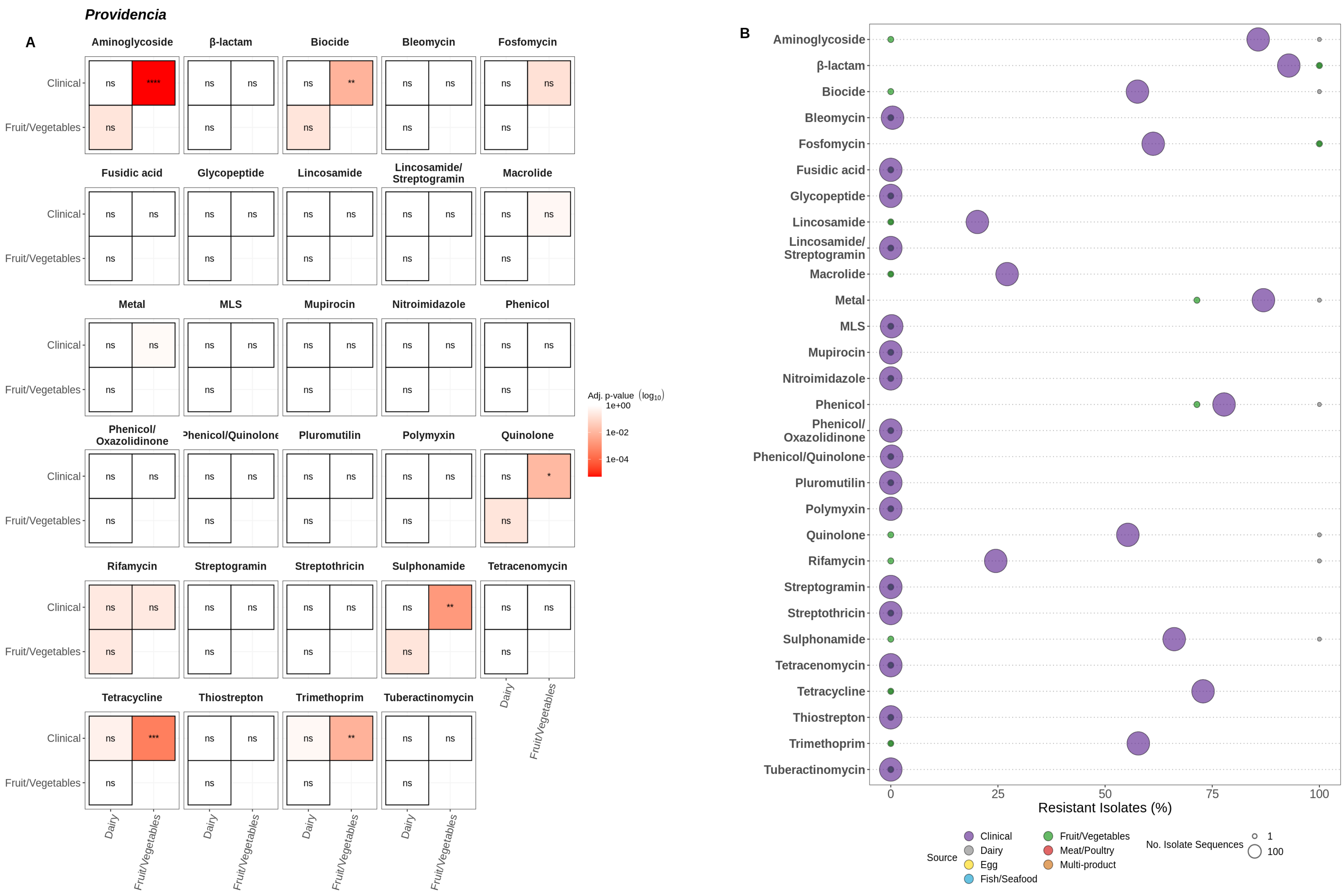
Providencia
A
Aminoglycoside
β-lactam
Biocide
Bleomycin
Fosfomycin
Fusidic acid
Glycopeptide
Lincosamide
Lincosamide/ Streptogramin
Macrolide
Metal
MLS
Mupirocin
Nitroimidazole
Phenicol
Phenicol/ Oxazolidinone
Phenicol/Quinolone
Pluromutilin
Polymyxin
Quinolone
Rifamycin
Streptogramin
Streptothricin
Sulphonamide
Tetracenomycin
Tetracycline
Thiostrepton
Trimethoprim
Tuberactinomycin
Clinical
Fruit/Vegetables
Dairy
ns
****
**
*
***
Adj. p-value (log10)
1e+00
1e-02
1e-04
B
Resistant Isolates (%)
0
25
50
75
100
Source
Clinical
Dairy
Egg
Fish/Seafood
Fruit/Vegetables
Meat/Poultry
Multi-product
No. Isolate Sequences
1
100

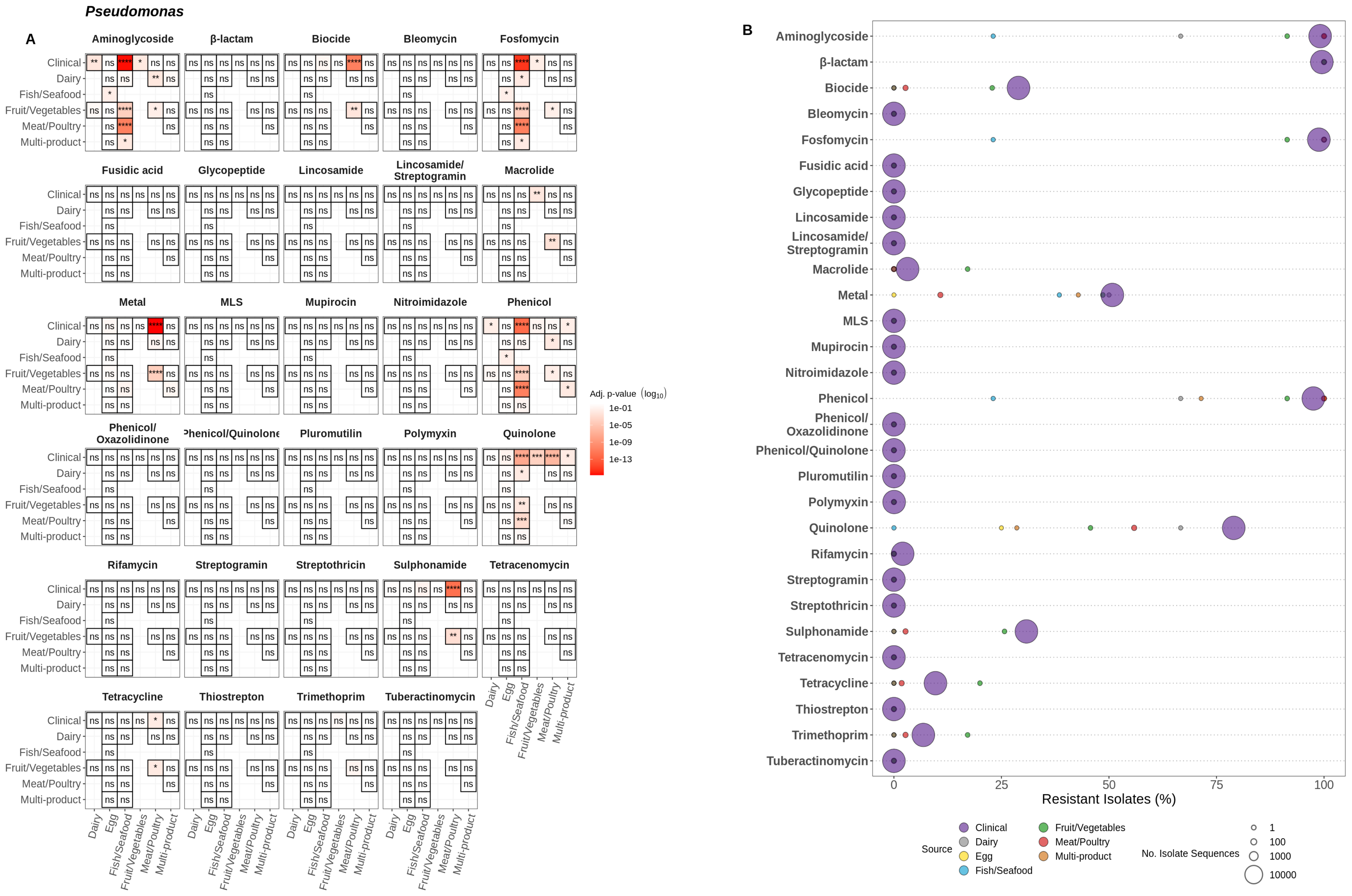
Pseudomonas
A
Aminoglycoside
β-lactam
Biocide
Bleomycin
Fosfomycin
Fusidic acid
Glycopeptide
Lincosamide
Lincosamide/ Streptogramin
Macrolide
Metal
MLS
Mupirocin
Nitroimidazole
Phenicol
Phenicol/ Oxazolidinone
Phenicol/Quinolone
Pluromutilin
Polymyxin
Quinolone
Rifamycin
Streptogramin
Streptothricin
Sulphonamide
Tetracenomycin
Tetracycline
Thiostrepton
Trimethoprim
Tuberactinomycin
Clinical
Dairy
Fish/Seafood
Fruit/Vegetables
Meat/Poultry
Multi-product
Egg
Adj. p-value (log10)
1e-01
1e-05
1e-09
1e-13
B
Resistant Isolates (%)
0
25
50
75
100
Source
No. Isolate Sequences
1
100
1000
10000

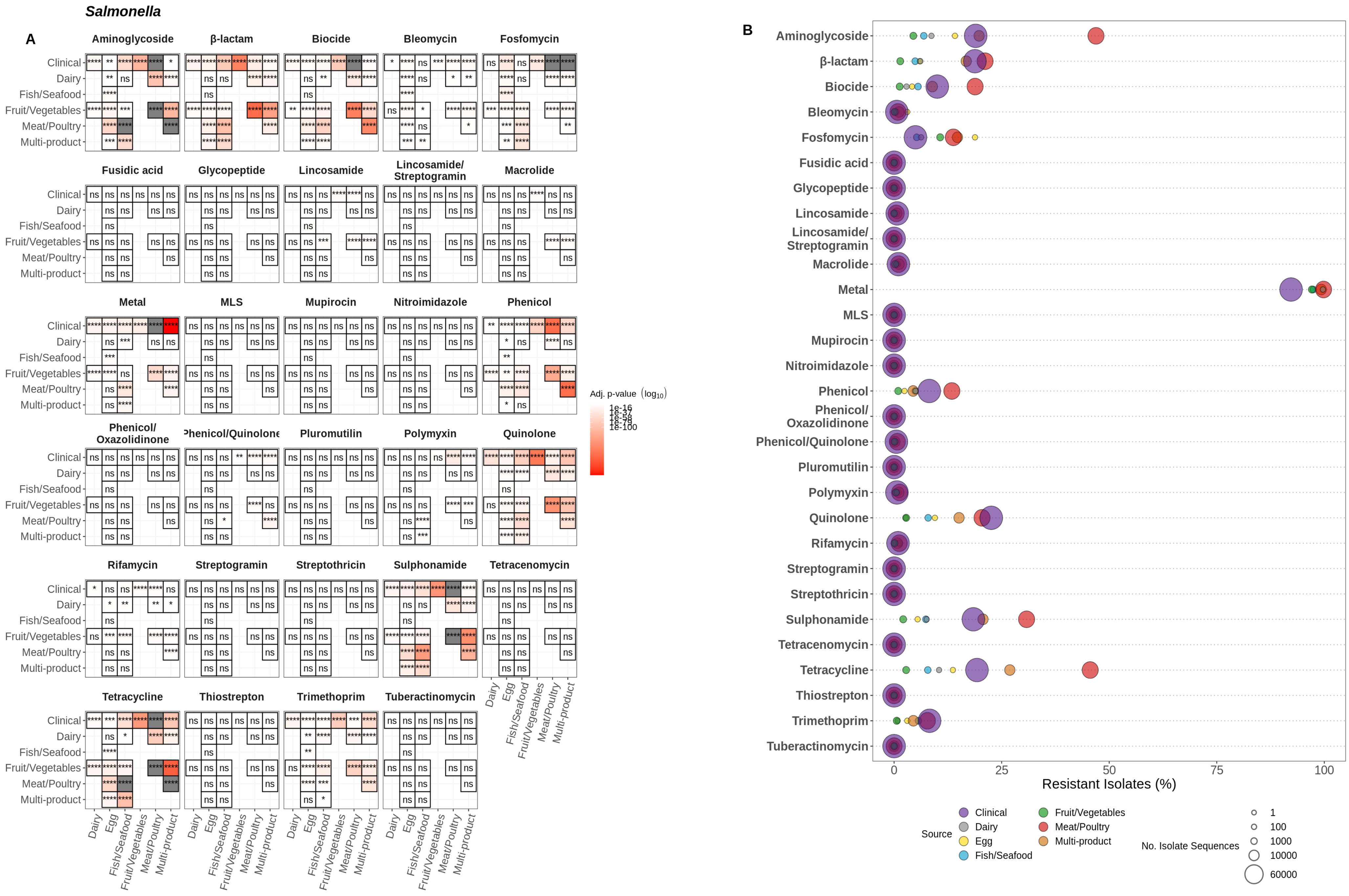

Salmonella
A
Aminoglycoside
β-lactam
Biocide
Bleomycin
Fosfomycin
Fusidic acid
Glycopeptide
Lincosamide
Lincosamide/ Streptogramin
Macrolide
Metal
MLS
Mupirocin
Nitroimidazole
Phenicol
Phenicol/ Oxazolidinone
Phenicol/Quinolone
Pluromutilin
Polymyxin
Quinolone
Rifamycin
Streptogramin
Streptothricin
Sulphonamide
Tetracenomycin
Tetracycline
Thiostrepton
Trimethoprim
Tuberactinomycin
Clinical
Dairy
Fish/Seafood
Fruit/Vegetables
Meat/Poultry
Multi-product
Egg
Adj. p-value (log10)
1e-16
1e-37
1e-58
1e-79
1e-100
B
0
25
50
75
100
Resistant Isolates (%)
Source
Clinical
Dairy
Egg
Fish/Seafood
Fruit/Vegetables
Meat/Poultry
Multi-product
No. Isolate Sequences
1
100
1000
10000
60000

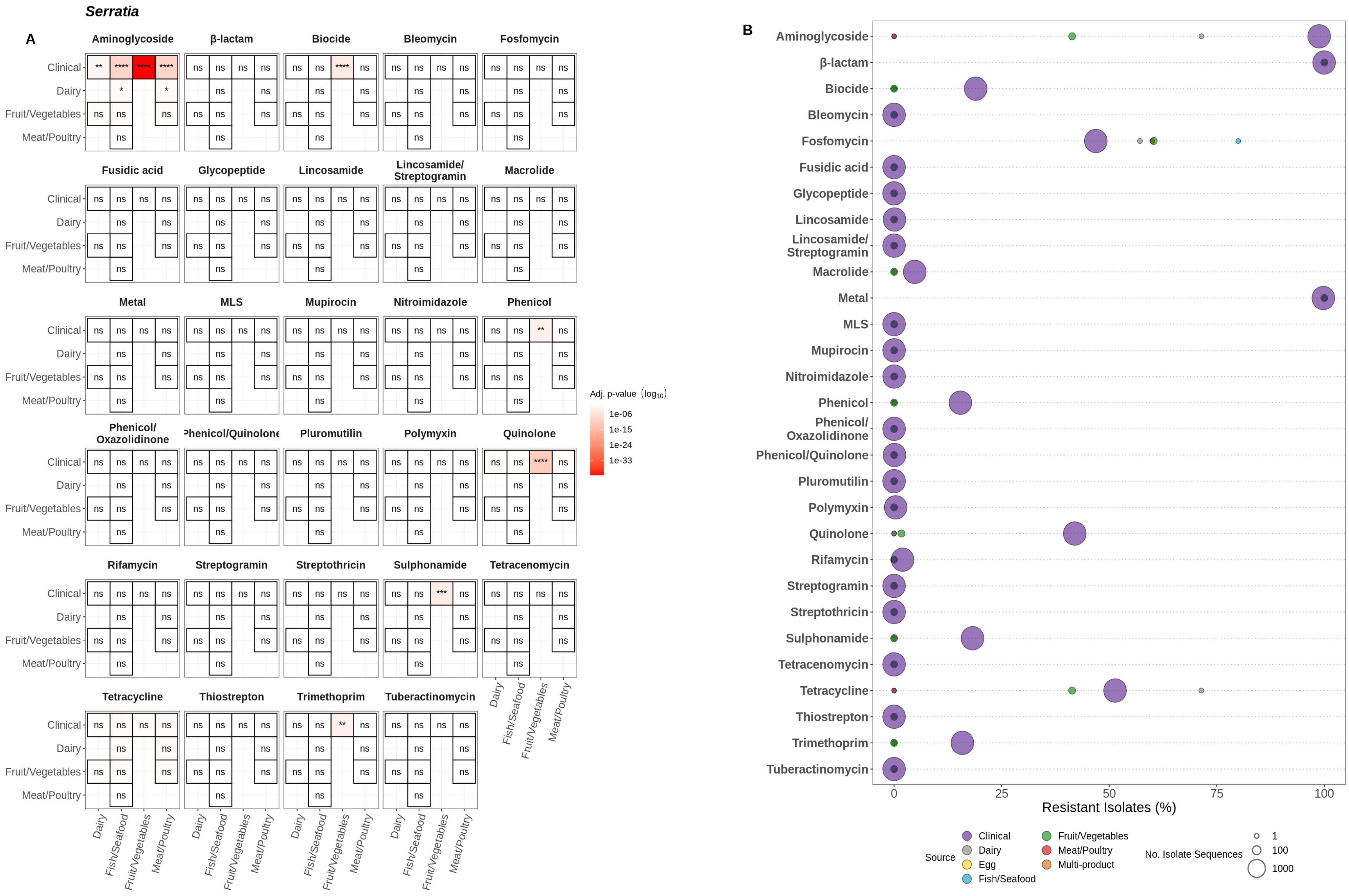

Serratia
A
Aminoglycoside
β-lactam
Biocide
Bleomycin
Fosfomycin
Fusidic acid
Glycopeptide
Lincosamide
Lincosamide/ Streptogramin
Macrolide
Metal
MLS
Mupirocin
Nitroimidazole
Phenicol
Phenicol/ Oxazolidinone
Phenicol/Quinolone
Pluromutilin
Polymyxin
Quinolone
Rifamycin
Streptogramin
Streptothricin
Sulphonamide
Tetracenomycin
Tetracycline
Thiostrepton
Trimethoprim
Tuberactinomycin
Clinical
Dairy
Fruit/Vegetables
Meat/Poultry
Fish/Seafood
Adj. p-value (log10)
1e-06
1e-15
1e-24
1e-33
B
Resistant Isolates (%)
0
25
50
75
100
Source
Clinical
Dairy
Egg
Fish/Seafood
Fruit/Vegetables
Meat/Poultry
Multi-product
No. Isolate Sequences
1
100
1000

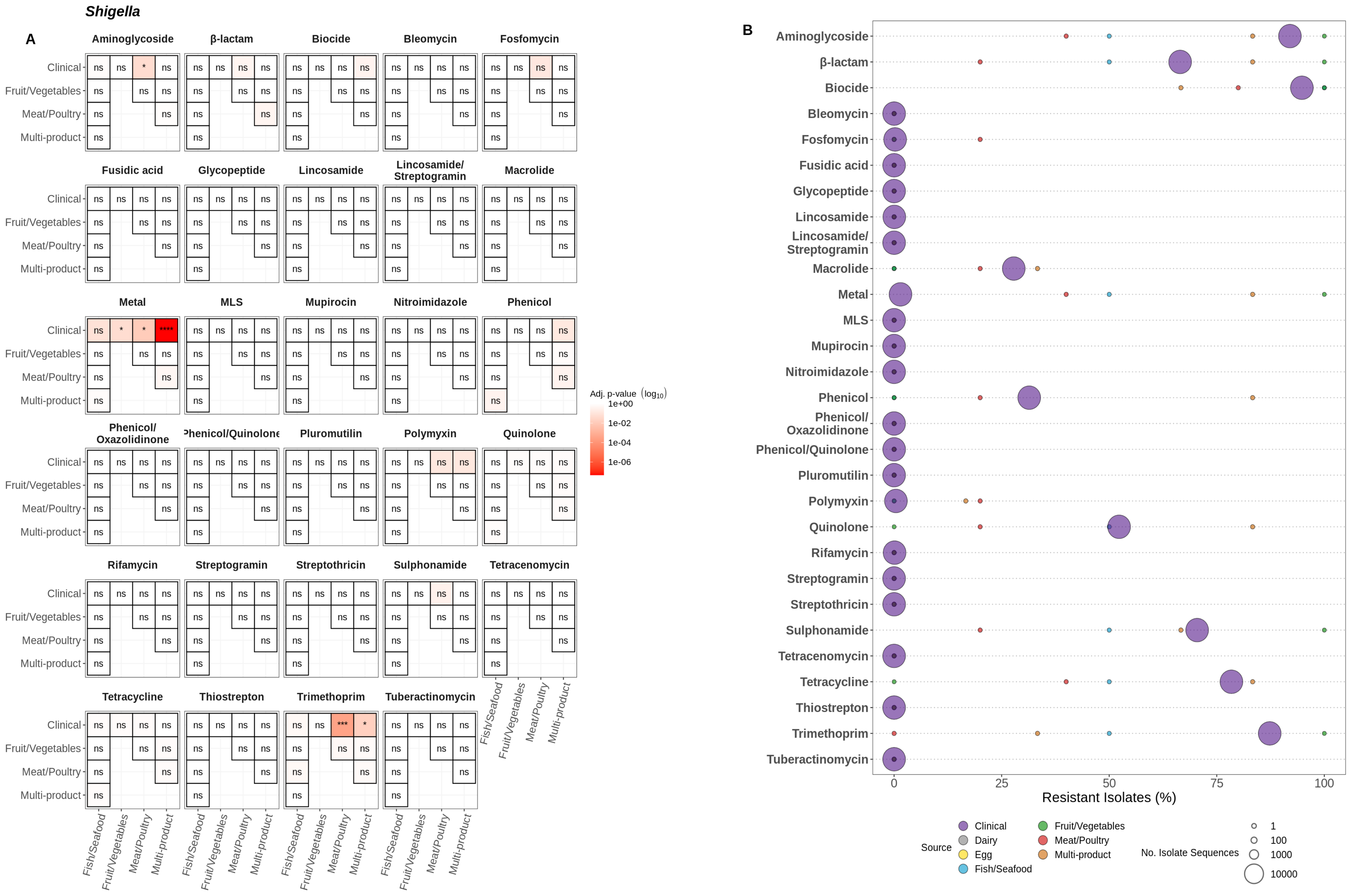
Shigella
A
B
Aminoglycoside
β-lactam
Biocide
Bleomycin
Fosfomycin
Fusidic acid
Glycopeptide
Lincosamide
Lincosamide/ Streptogramin
Macrolide
Metal
MLS
Mupirocin
Nitroimidazole
Phenicol
Phenicol/ Oxazolidinone
Phenicol/Quinolone
Pluromutilin
Polymyxin
Quinolone
Rifamycin
Streptogramin
Streptothricin
Sulphonamide
Tetracenomycin
Tetracycline
Thiostrepton
Trimethoprim
Tuberactinomycin
Clinical
Fruit/Vegetables
Meat/Poultry
Multi-product
Fish/Seafood
Adj. p-value (log10)
1e+00
1e-02
1e-04
1e-06
Resistant Isolates (%)
0
25
50
75
100
Source
Clinical
Dairy
Egg
Fish/Seafood
Fruit/Vegetables
Meat/Poultry
Multi-product
No. Isolate Sequences
1
100
1000
10000

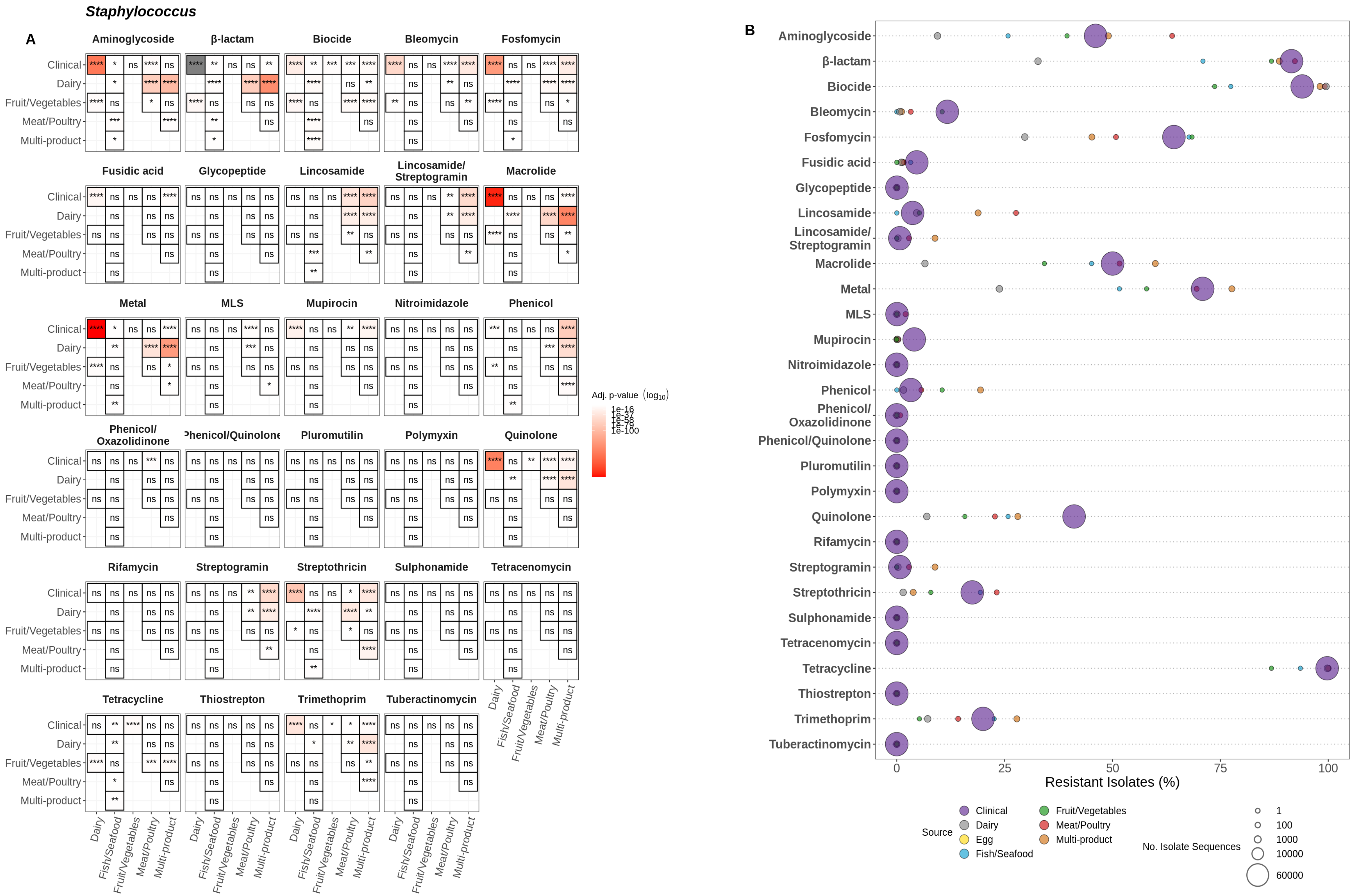
Staphylococcus
A
Aminoglycoside
β-lactam
Biocide
Bleomycin
Fosfomycin
Fusidic acid
Glycopeptide
Lincosamide
Lincosamide/ Streptogramin
Macrolide
Metal
MLS
Mupirocin
Nitroimidazole
Phenicol
Phenicol/ Oxazolidinone
Phenicol/Quinolone
Pluromutilin
Polymyxin
Quinolone
Rifamycin
Streptogramin
Streptothricin
Sulphonamide
Tetracenomycin
Tetracycline
Thiostrepton
Trimethoprim
Tuberactinomycin
Clinical
Dairy
Fruit/Vegetables
Meat/Poultry
Multi-product
Fish/Seafood
Adj. p-value (log10)
1e-16
1e-37
1e-58
1e-79
1e-100
B
Resistant Isolates (%)
0
25
50
75
100
Source
Clinical
Dairy
Egg
Fish/Seafood
Fruit/Vegetables
Meat/Poultry
Multi-product
No. Isolate Sequences
1
100
1000
10000
60000

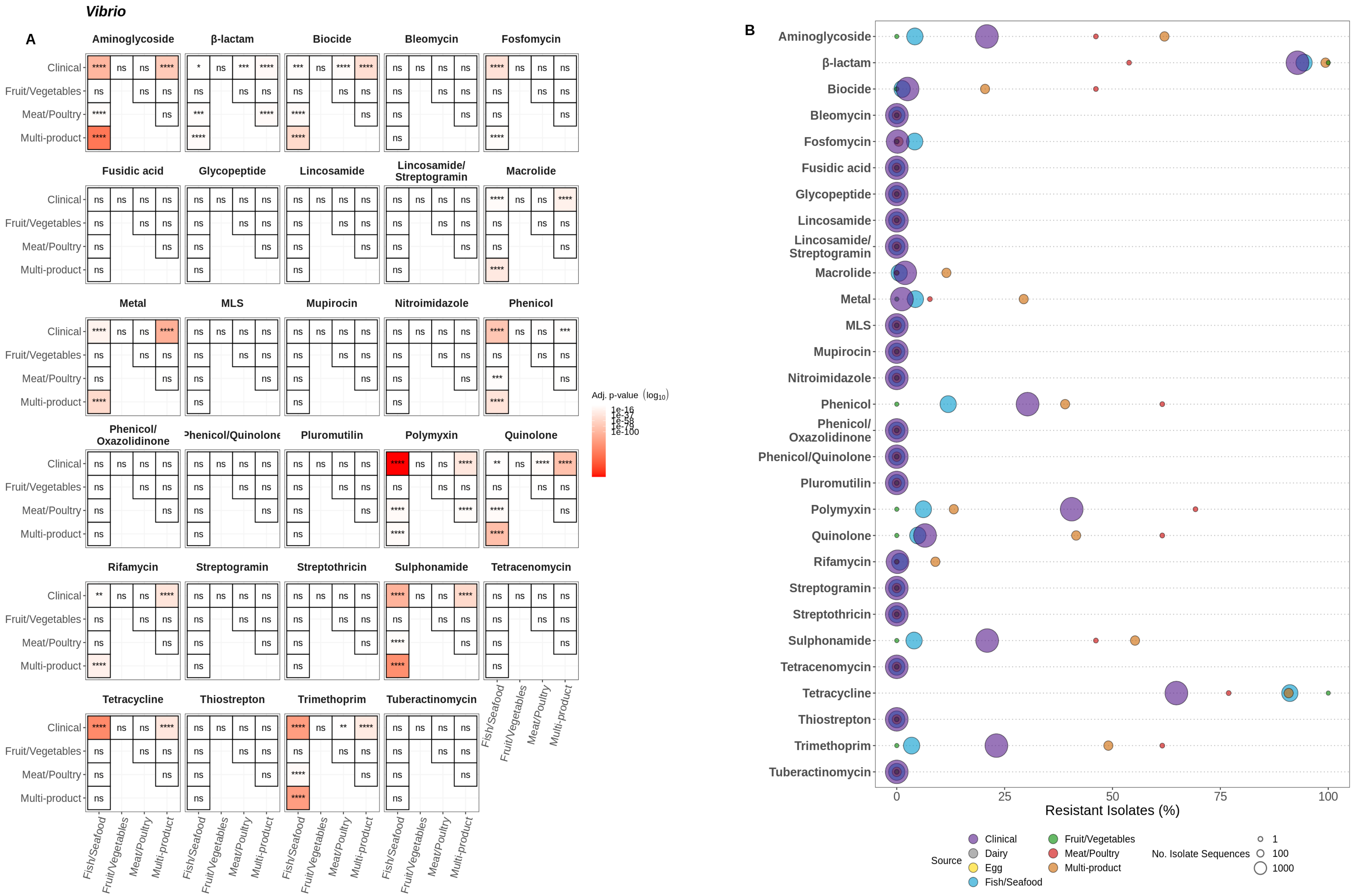
Vibrio
A
Aminoglycoside
β-lactam
Biocide
Bleomycin
Fosfomycin
Fusidic acid
Glycopeptide
Lincosamide
Lincosamide/ Streptogramin
Macrolide
Metal
MLS
Mupirocin
Nitroimidazole
Phenicol
Phenicol/ Oxazolidinone
Phenicol/Quinolone
Pluromutilin
Polymyxin
Quinolone
Rifamycin
Streptogramin
Streptothricin
Sulphonamide
Tetracenomycin
Tetracycline
Thiostrepton
Trimethoprim
Tuberactinomycin
Clinical
Fruit/Vegetables
Meat/Poultry
Multi-product
Fish/Seafood
Adj. p-value (log10)
1e-16
1e-37
1e-58
1e-79
1e-100
B
Resistant Isolates (%)
0
25
50
75
100
Source
Clinical
Dairy
Egg
Fish/Seafood
Fruit/Vegetables
Meat/Poultry
Multi-product
No. Isolate Sequences
1
100
1000
