# Supplementary material for: Analysis of Antimicrobial Resistance in Bacterial Pathogens Recovered from Food and Human Sources: Insights from 639,087 Bacterial Whole-Genome Sequences in the NCBI Pathogen Detection Database
Source: Microorganisms. 2024 Mar 30;12(4):709. doi: 10.3390/microorganisms12040709 (PMC11051753; doi:10.3390/microorganisms12040709)
Supplement: Supplementary file 1 [file microorganisms-12-00709-s001.zip › Supplementary File S3.pdf]

Supplementary File S3

The following pages contain supplementary figures for correlation analysis of the resistance levels for each genus (top left of page). For each genus, chi-square was performed for each isolation source for which there was at least one sequence/isolate. Correlation plots were created for each source (top panel headings) using the Pearson standardised residuals output by Chi square analysis. The Pearson standardised residuals measure the departure of each cell from independence, thereby showing the strength and direction of association. Positive residuals are blue and specify a positive association or attraction of resistance between corresponding antimicrobial class (left row labels) and source (column label). Red fill indicates a negative association or repulsion between antimicrobial class and isolation source.The filled portion of the pie indicates the magnitude of the correlation (eg. amount of the cell contribution (resistance class) to the overall test statistic for that isolation source). Legend bars differ between genera and sources, and indicate the standardised residual.

Acinetobacter

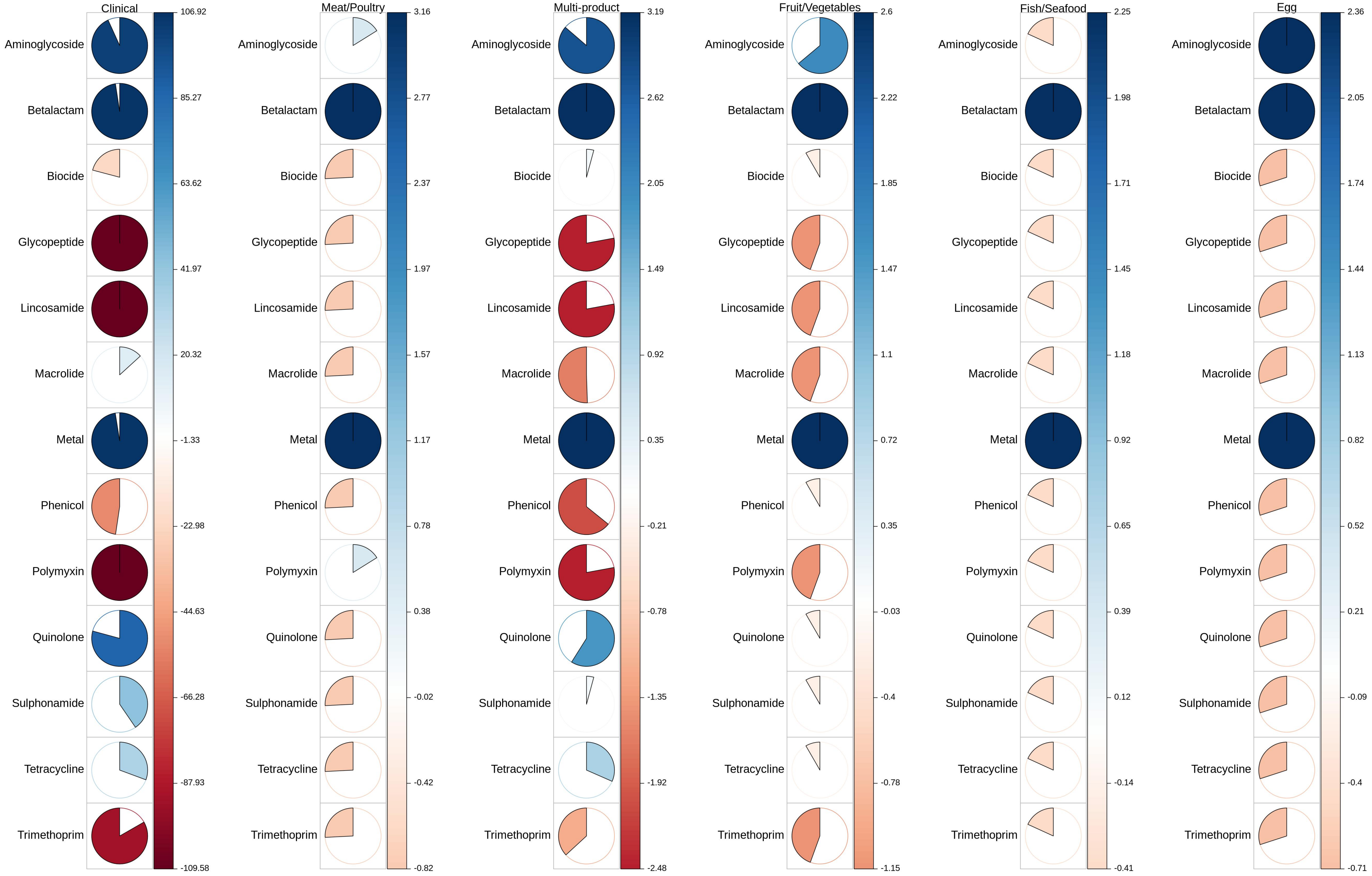

Aeromonas

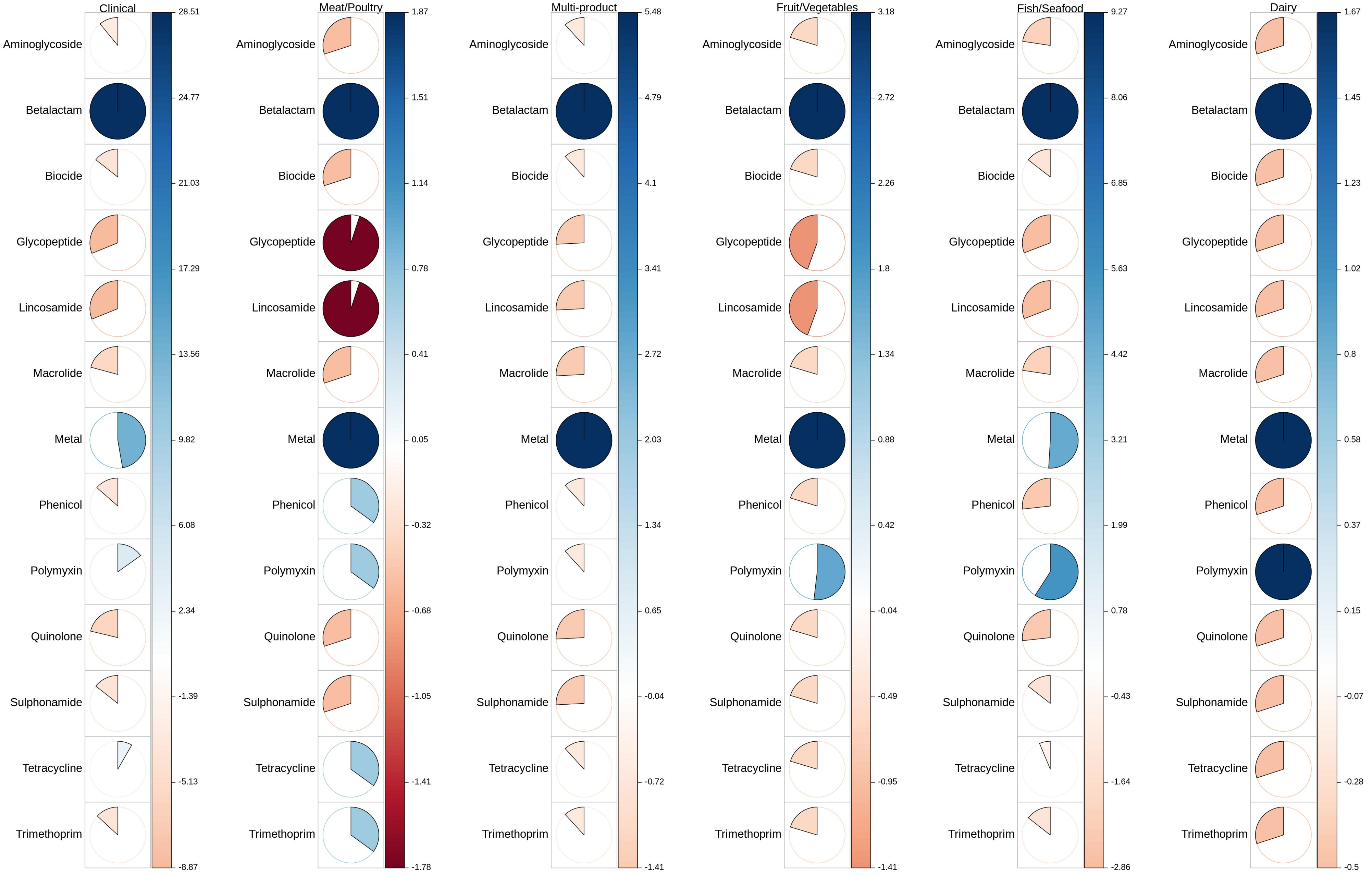
Clinical
Meat/Poultry
Multi-product
Fruit/Vegetables
Fish/Seafood
Dairy
Aminoglycoside
Betalactam
Biocide
Glycopeptide
Lincosamide
Macrolide
Metal
Phenicol
Polymyxin
Quinolone
Sulphonamide
Tetracycline
Trimethoprim
28.51
24.77
21.03
17.29
13.56
9.82
6.08
2.34
-1.39
-5.13
-8.87
1.87
1.51
1.14
0.78
0.41
0.05
-0.32
-0.68
-1.05
-1.41
-1.78
5.48
4.79
4.1
3.41
2.72
2.03
1.34
0.65
-0.04
-0.72
-1.41
3.18
2.72
2.26
1.8
1.34
0.88
0.42
-0.04
-0.49
-0.95
-1.41
9.27
8.06
6.85
5.63
4.42
3.21
1.99
0.78
-0.43
-1.64
-2.86
1.67
1.45
1.23
1.02
0.8
0.58
0.37
0.15
-0.07
-0.28
-0.5

Bacillus

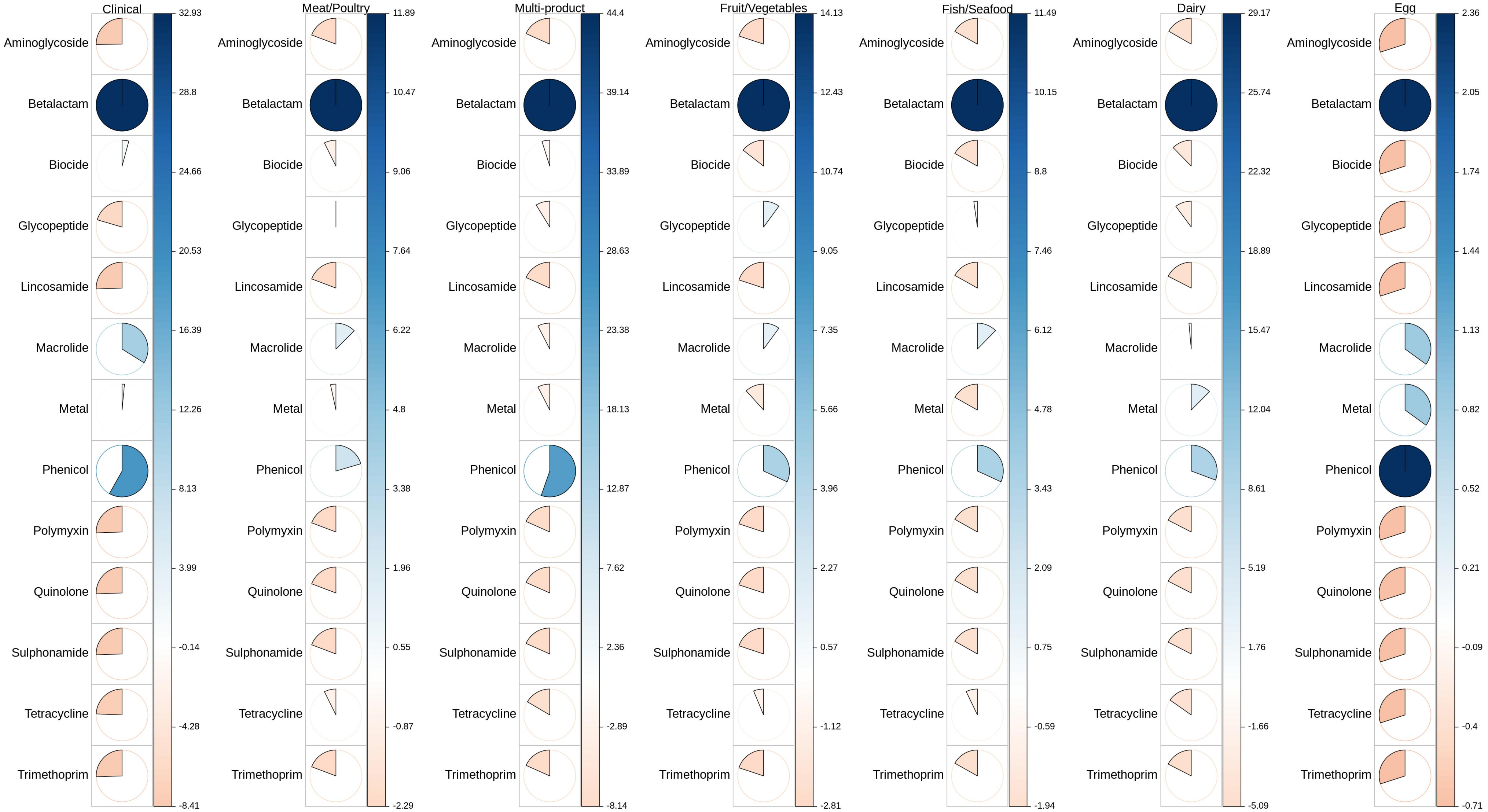
Clinical
Meat/Poultry
Multi-product
Fruit/Vegetables
Fish/Seafood
Dairy
Egg
Aminoglycoside
Betalactam
Biocide
Glycopeptide
Lincosamide
Macrolide
Metal
Phenicol
Polymyxin
Quinolone
Sulphonamide
Tetracycline
Trimethoprim
32.93 28.8 24.66 20.53 16.39 12.26 8.13 3.99 -0.14 -4.28 -8.41
11.89 10.47 9.06 7.64 6.22 4.8 3.38 1.96 0.55 -0.87 -2.29
44.4 39.14 33.89 28.63 23.38 18.13 12.87 7.62 2.36 -2.89 -8.14
14.13 12.43 10.74 9.05 7.35 5.66 3.96 2.27 0.57 -1.12 -2.81
11.49 10.15 8.8 7.46 6.12 4.78 3.43 2.09 0.75 -0.59 -1.94
29.17 25.74 22.32 18.89 15.47 12.04 8.61 5.19 1.76 -1.66 -5.09
2.36 2.05 1.74 1.44 1.13 0.82 0.52 0.21 -0.09 -0.4 -0.71

Campylobacter

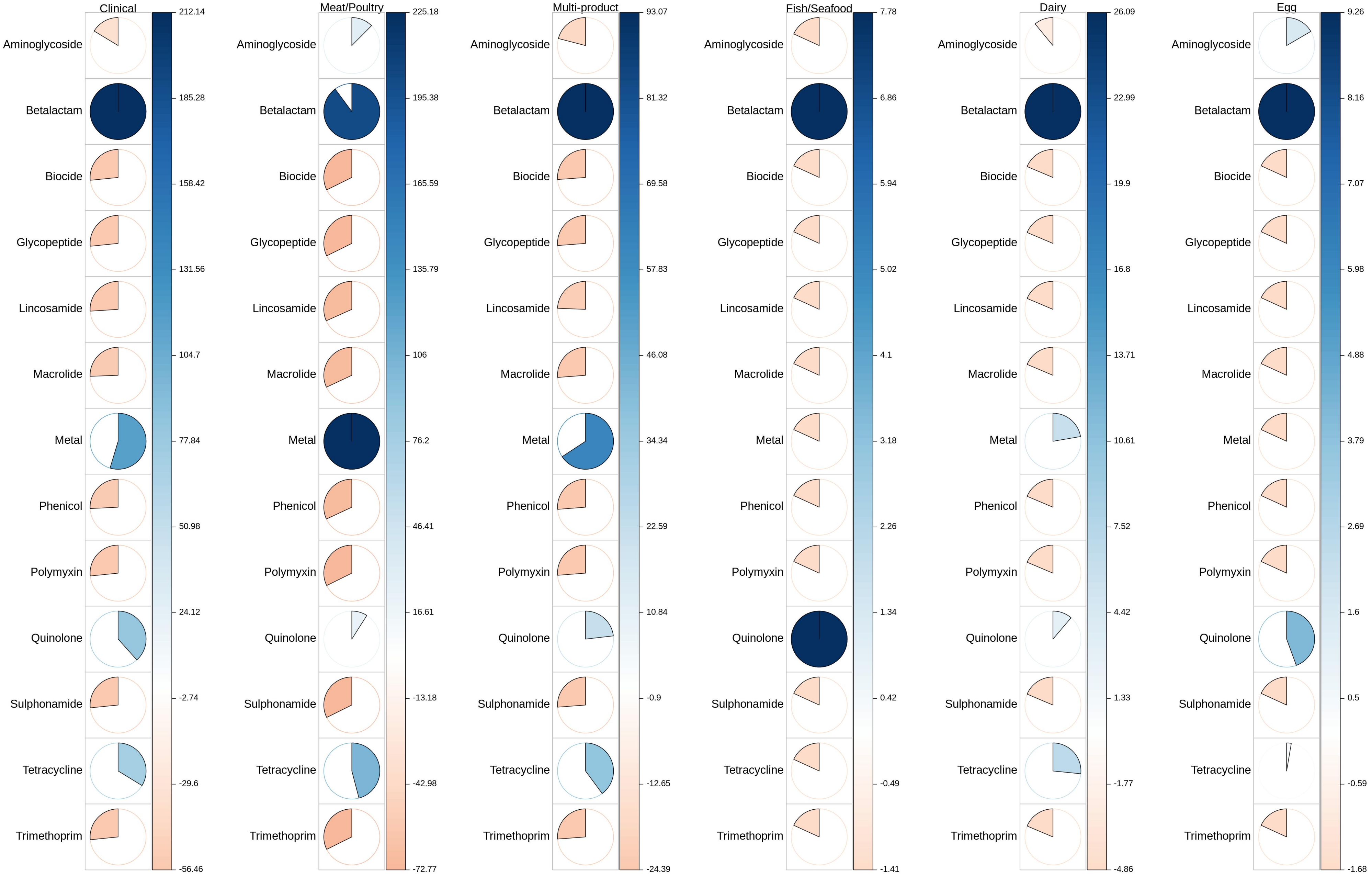

Citrobacter

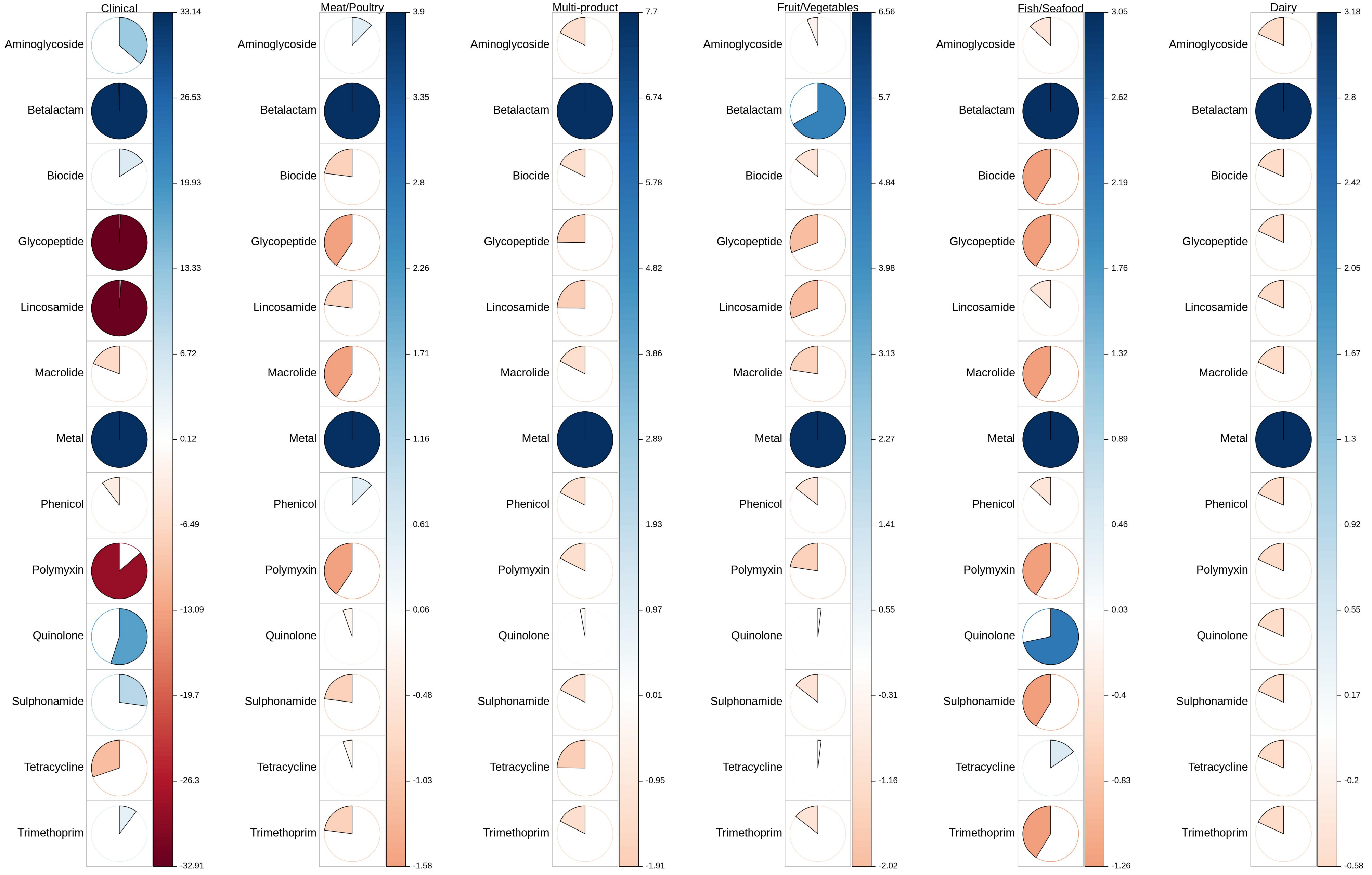

Clostridioides difficile

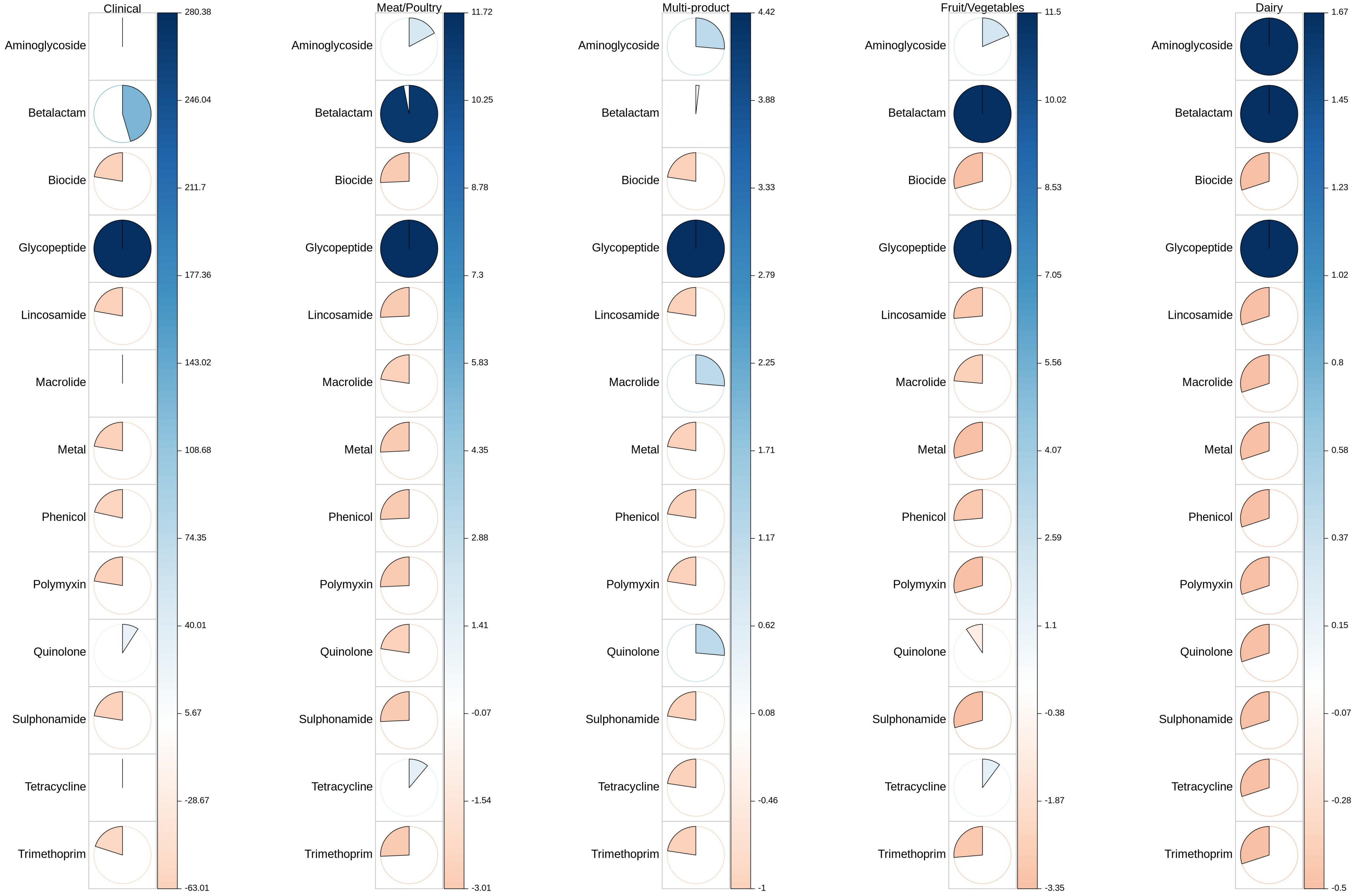

Clostridium

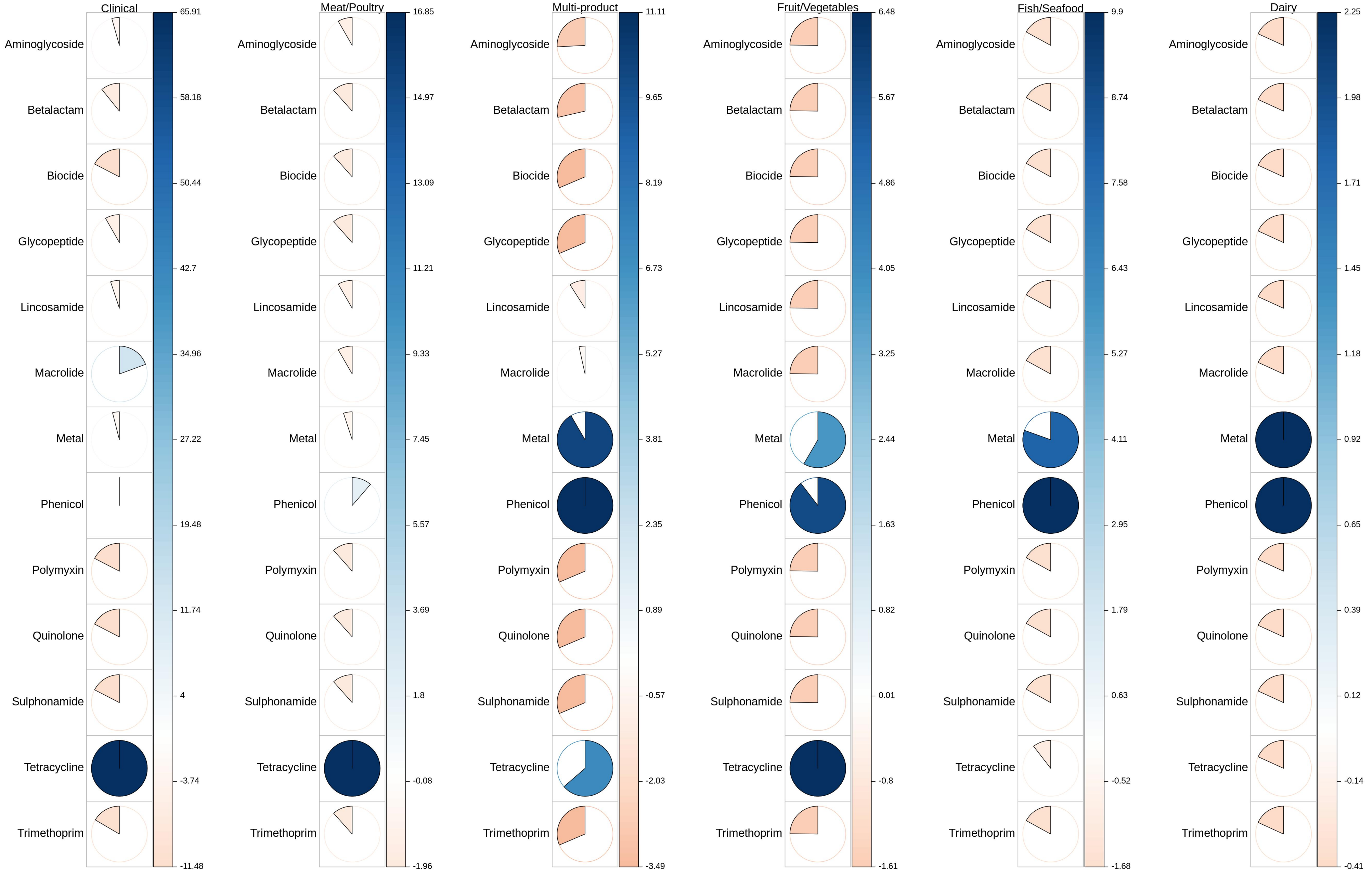

Enterobacter

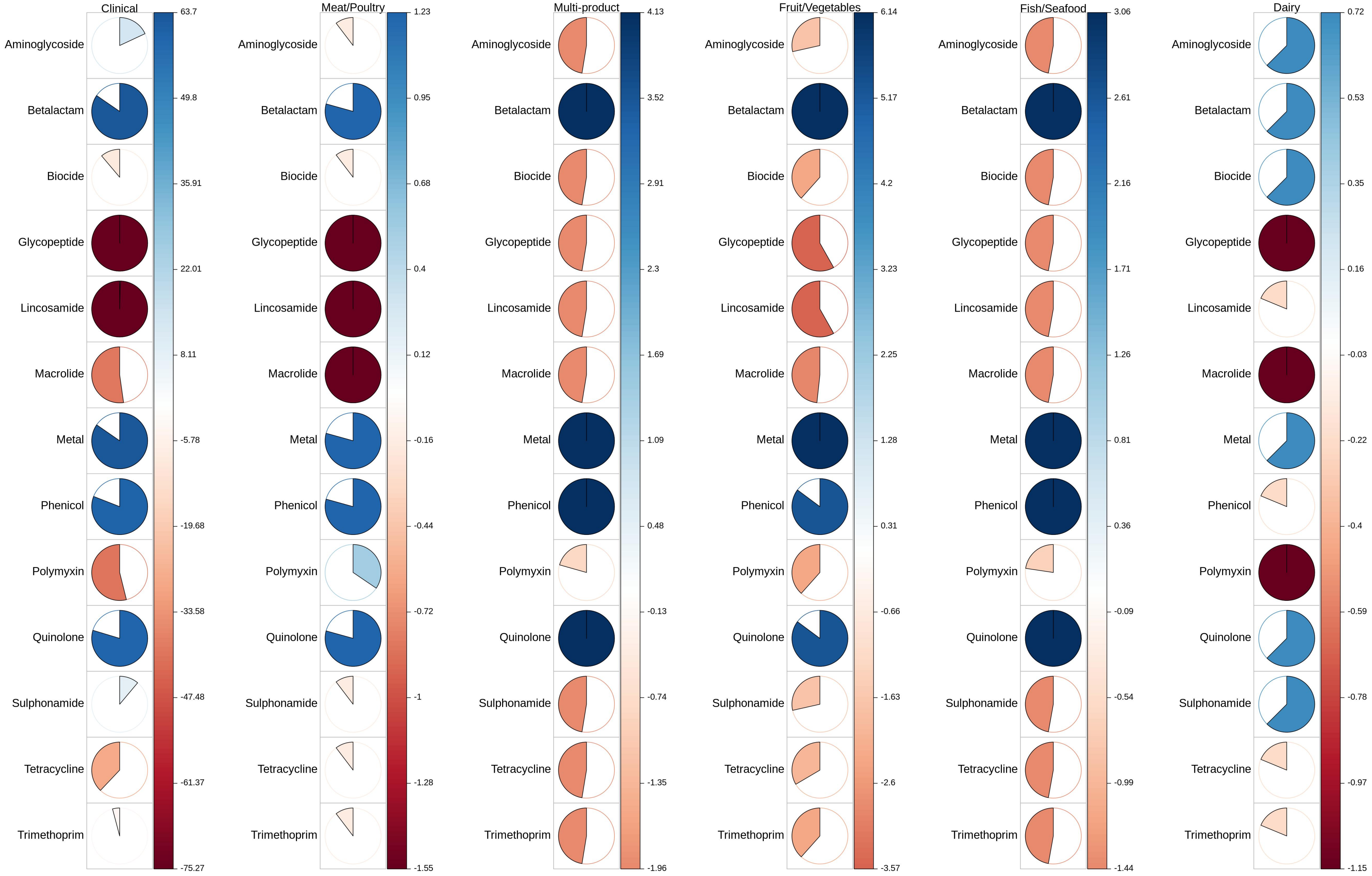

Enterococcus

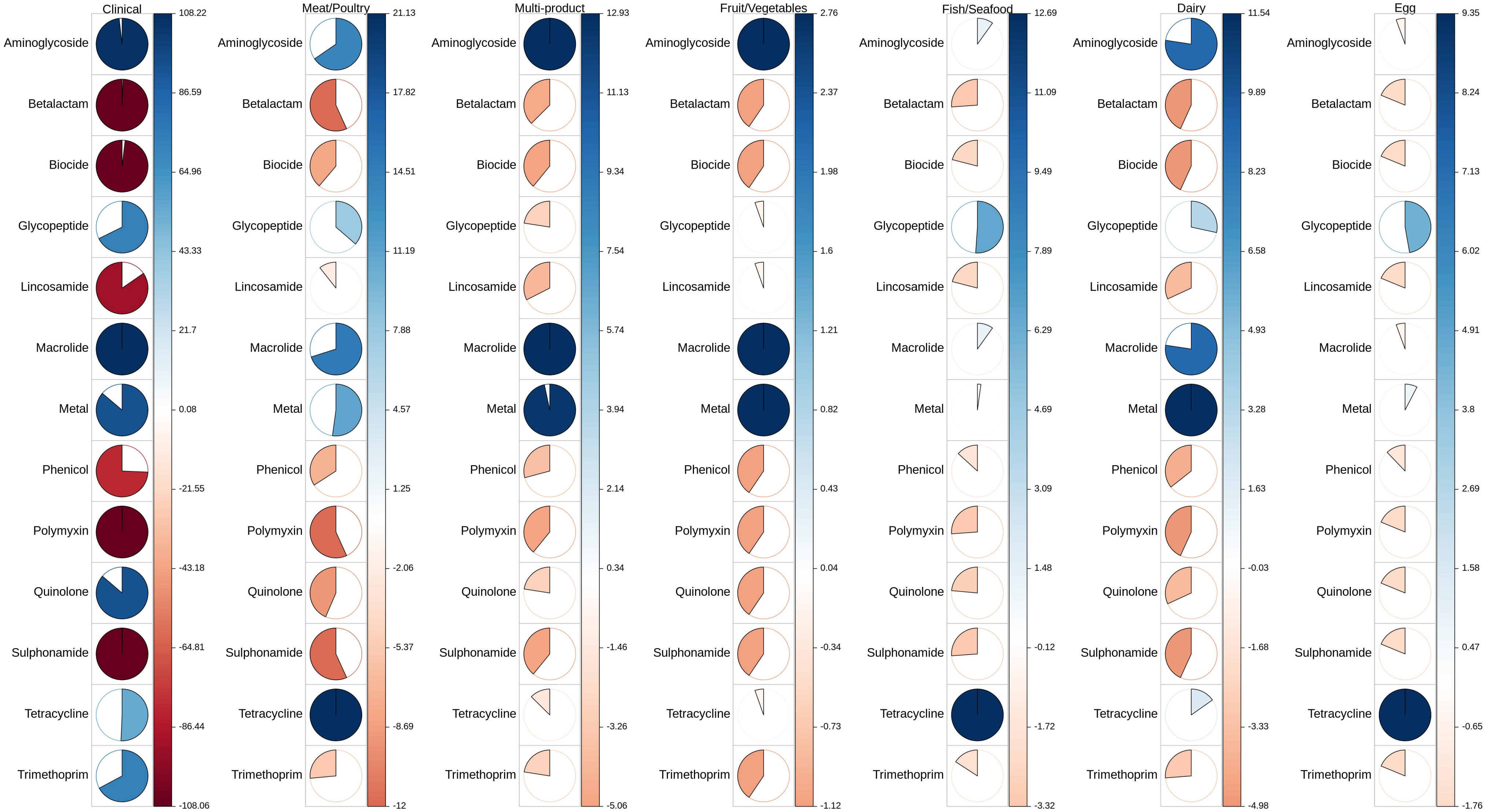

Escherichia

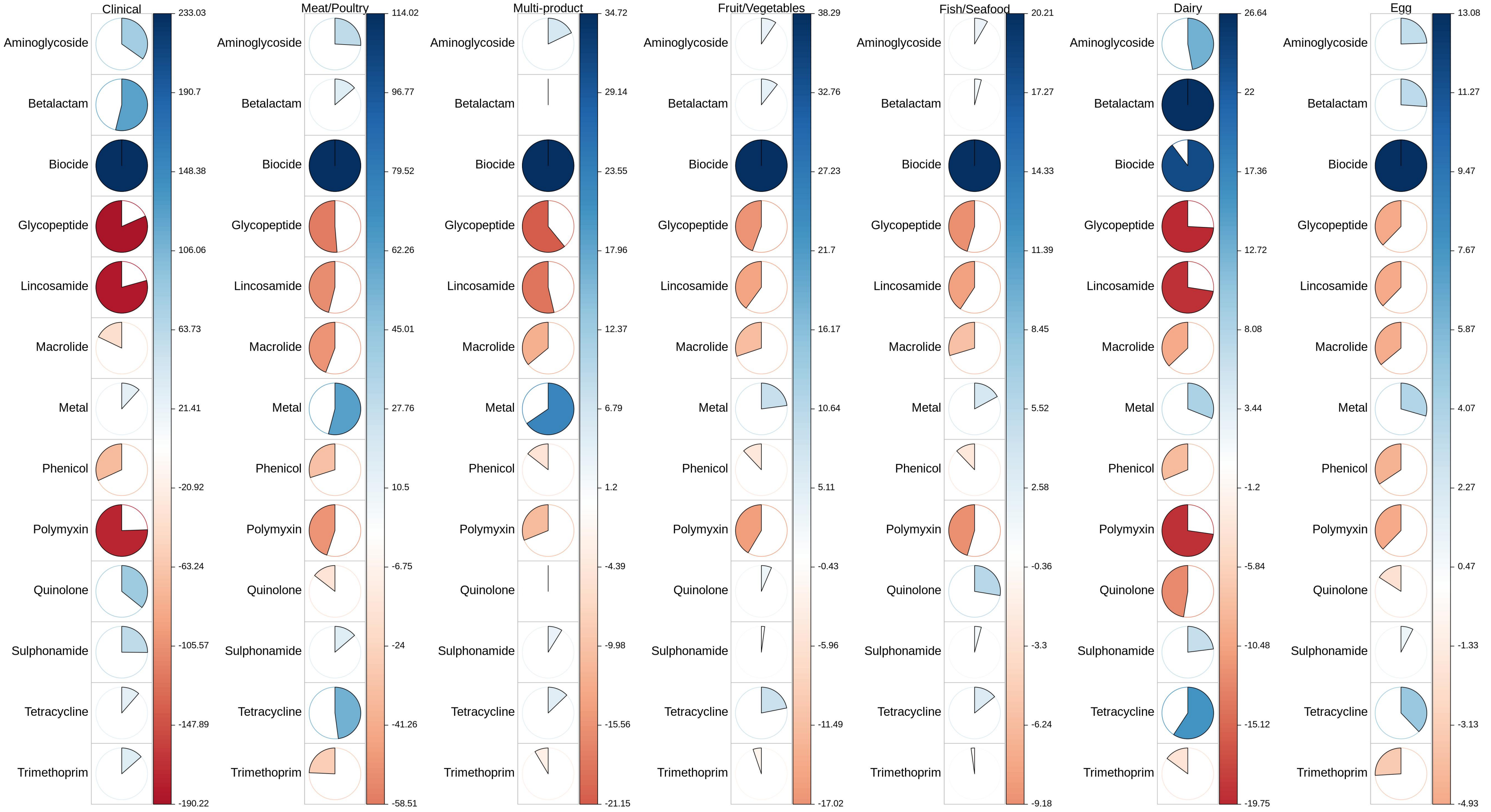

Klebsiella

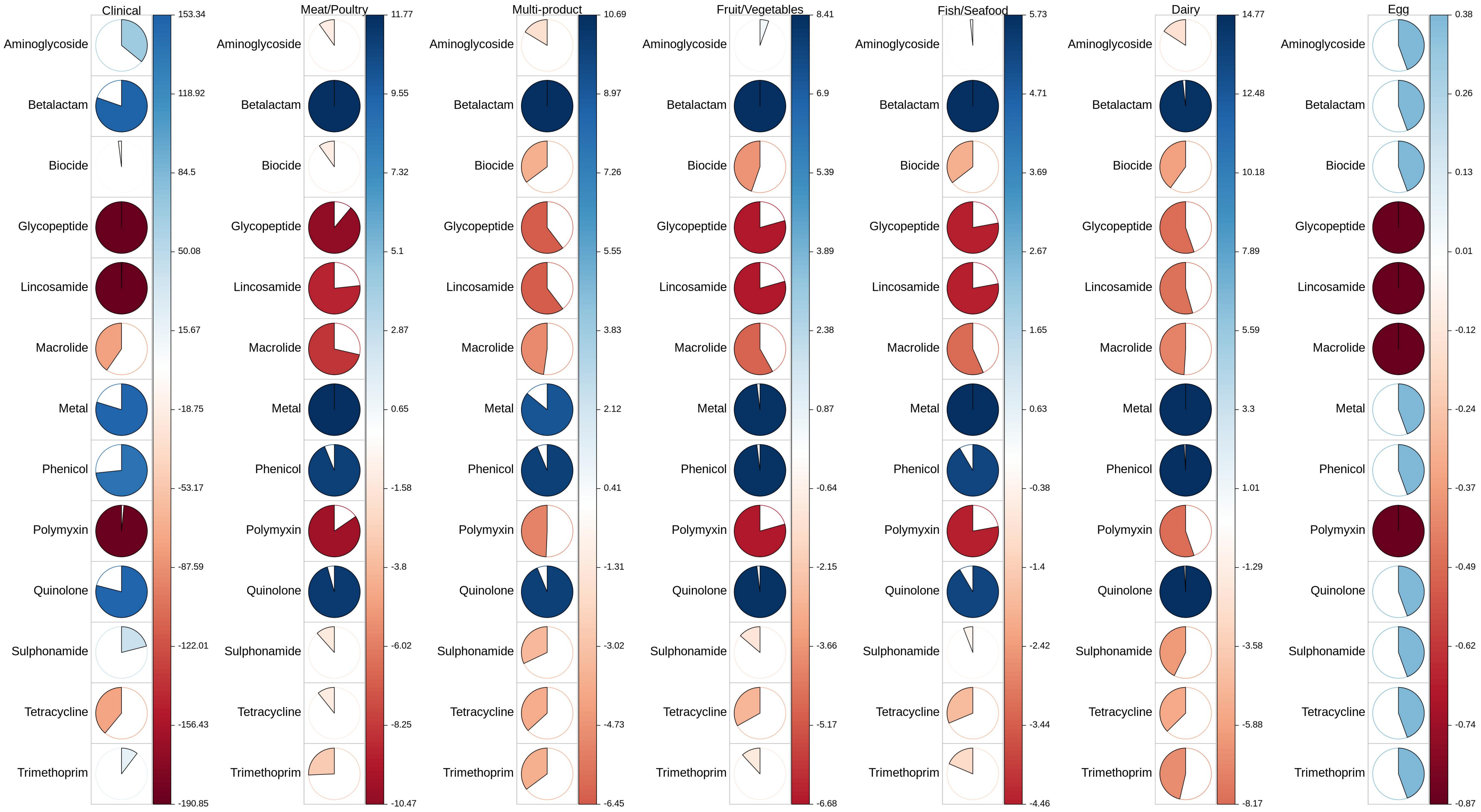
Clinical
Meat/Poultry
Multi-product
Fruit/Vegetables
Fish/Seafood
Dairy
Egg
Aminoglycoside
Betalactam
Biocide
Glycopeptide
Lincosamide
Macrolide
Metal
Phenicol
Polymyxin
Quinolone
Sulphonamide
Tetracycline
Trimethoprim
153.34 118.92 84.5 50.08 15.67 -18.75 -53.17 -87.59 -122.01 -156.43 -190.85
11.77 9.55 7.32 5.1 2.87 0.65 -1.58 -3.8 -6.02 -8.25 -10.47
10.69 8.97 7.26 5.55 3.83 2.12 0.41 -1.31 -3.02 -4.73 -6.45
8.41 6.9 5.39 3.89 2.38 0.87 -0.64 -2.15 -3.66 -5.17 -6.68
5.73 4.71 3.69 2.67 1.65 0.63 -0.38 -1.4 -2.42 -3.44 -4.46
14.77 12.48 10.18 7.89 5.59 3.3 1.01 -1.29 -3.58 -5.88 -8.17
0.38 0.26 0.13 0.01 -0.12 -0.24 -0.37 -0.49 -0.62 -0.74 -0.87

Kluyvera intermedia

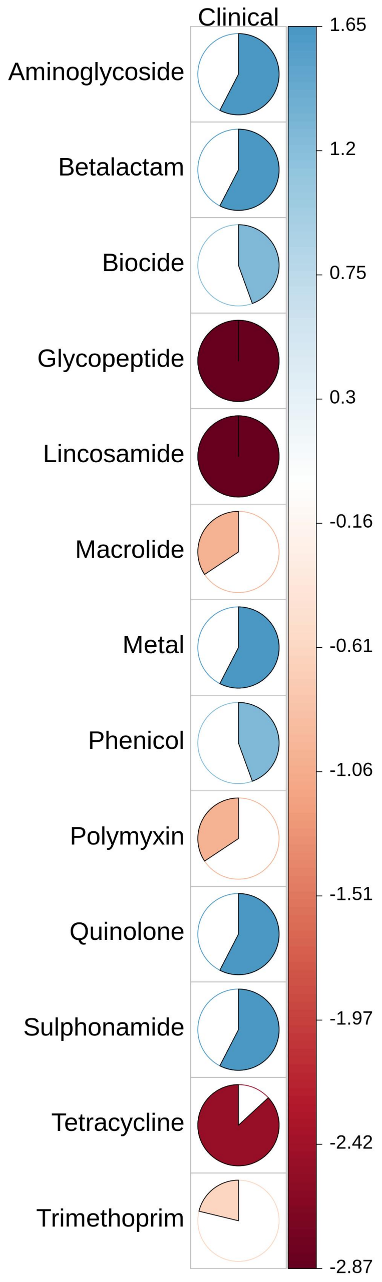

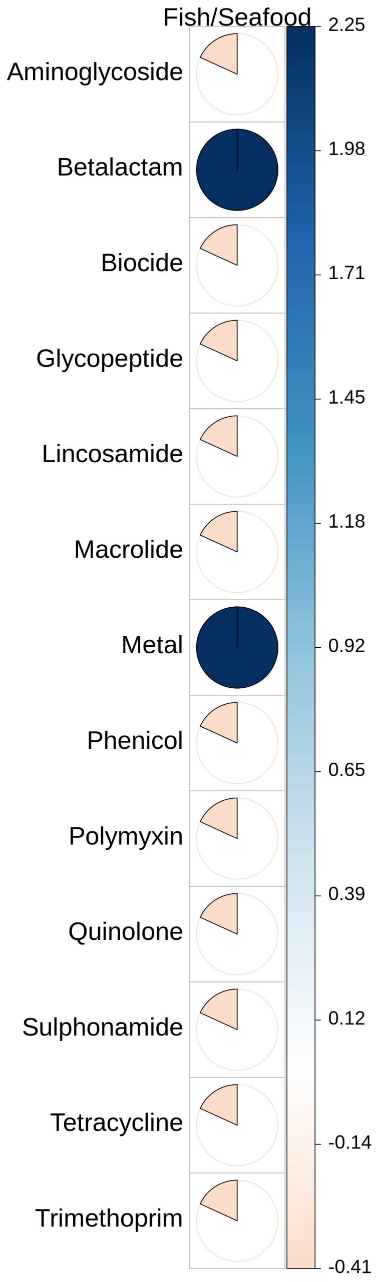

Listeria

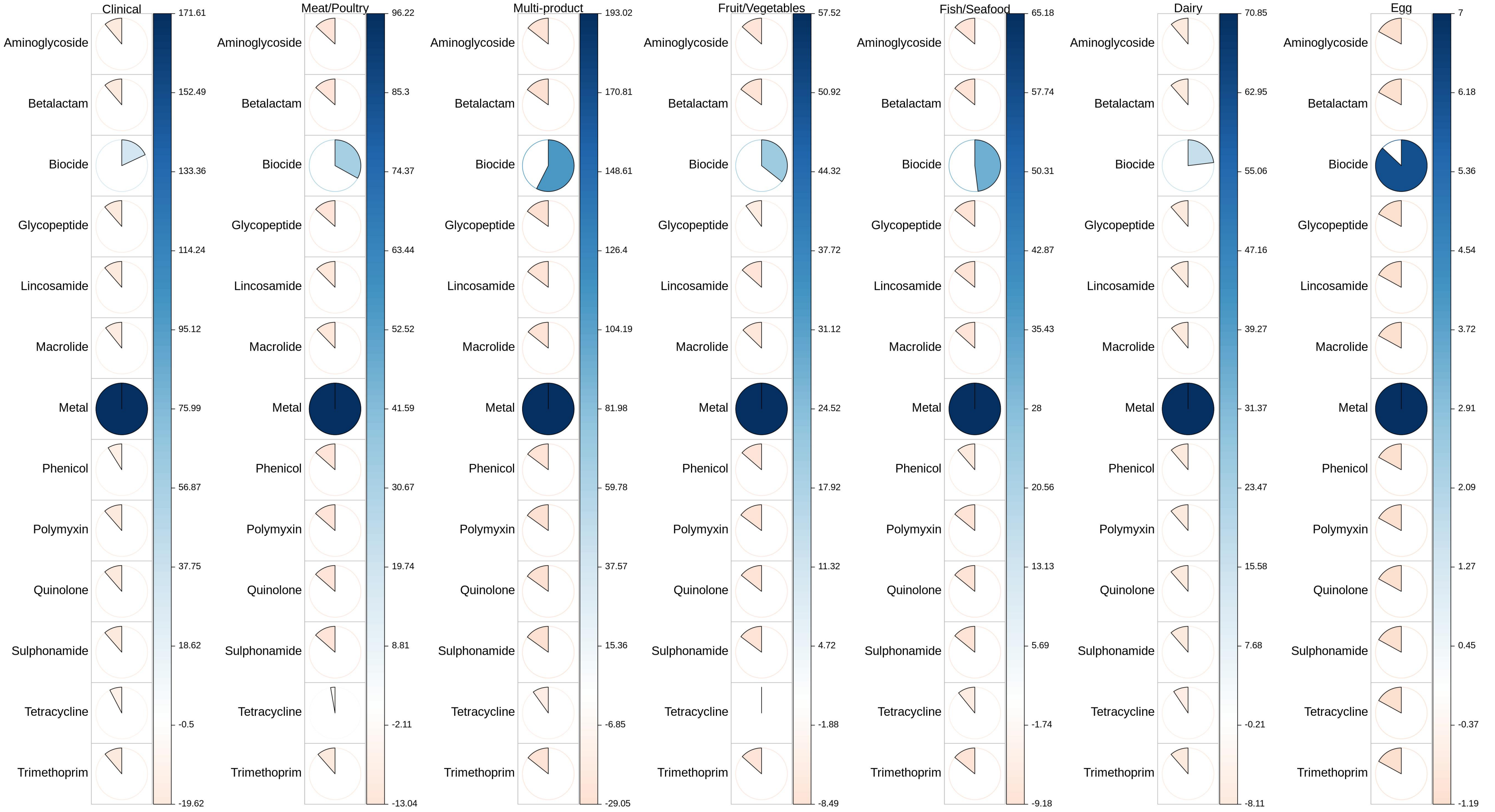
Clinical
Aminoglycoside
Betalactam
Biocide
Glycopeptide
Lincosamide
Macrolide
Metal
Phenicol
Polymyxin
Quinolone
Sulphonamide
Tetracycline
Trimethoprim
171.61
152.49
133.36
114.24
95.12
75.99
56.87
37.75
18.62
-0.5
-19.62
Meat/Poultry
96.22
85.3
74.37
63.44
52.52
41.59
30.67
19.74
8.81
-2.11
-13.04
Multi-product
193.02
170.81
148.61
126.4
104.19
81.98
59.78
37.57
15.36
-6.85
-29.05
Fruit/Vegetables
57.52
50.92
44.32
37.72
31.12
24.52
17.92
11.32
4.72
-1.88
-8.49
Fish/Seafood
65.18
57.74
50.31
42.87
35.43
28
20.56
13.13
5.69
-1.74
-9.18
Dairy
70.85
62.95
55.06
47.16
39.27
31.37
23.47
15.58
7.68
-0.21
-8.11
Egg
7
6.18
5.36
4.54
3.72
2.91
2.09
1.27
0.45
-0.37
-1.19

Morganella

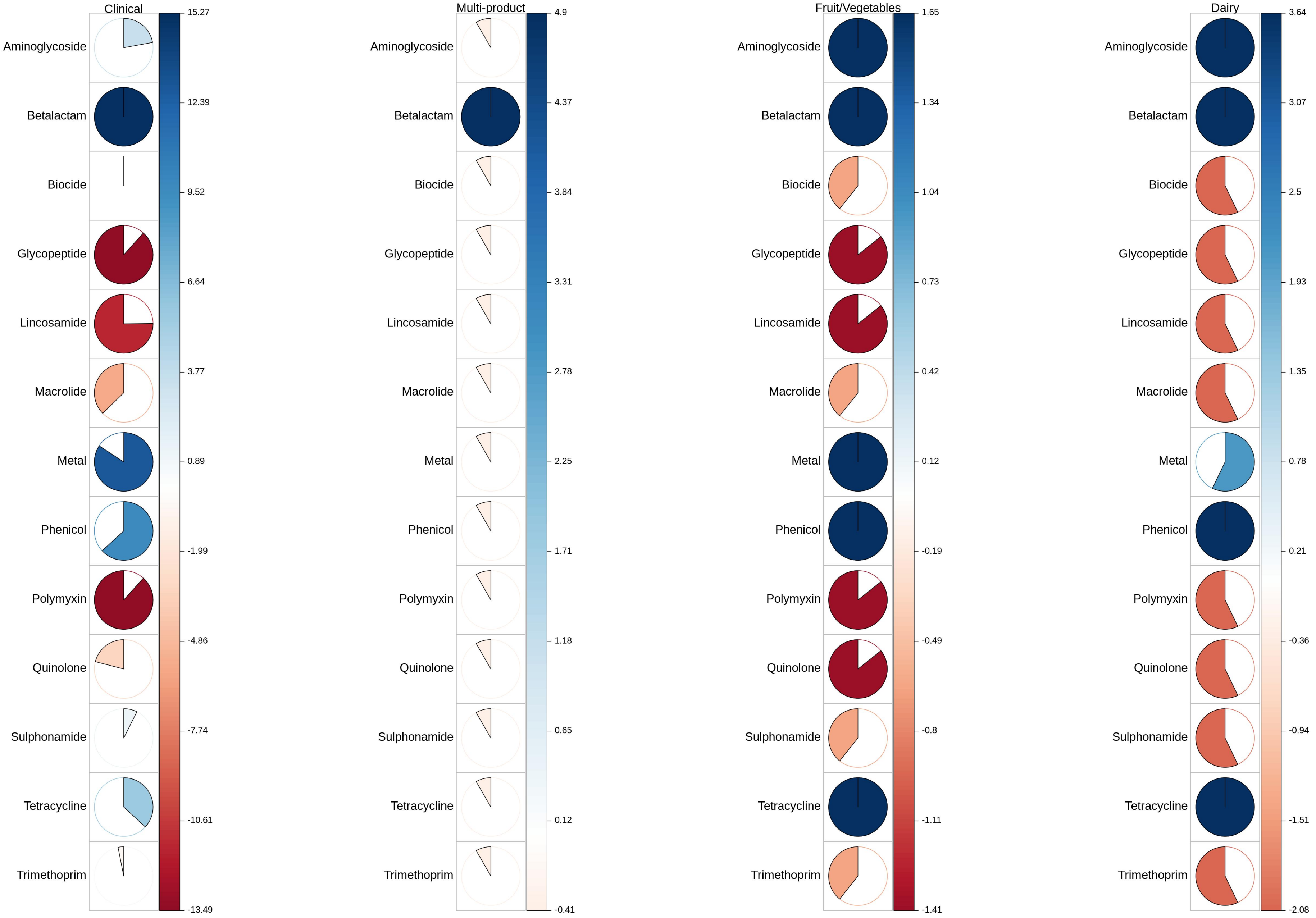

Clinical
Aminoglycoside
Betalactam
Biocide
Glycopeptide
Lincosamide
Macrolide
Metal
Phenicol
Polymyxin
Quinolone
Sulphonamide
Tetracycline
Trimethoprim
15.27
12.39
9.52
6.64
3.77
0.89
-1.99
-4.86
-7.74
-10.61
-13.49
Multi-product
Aminoglycoside
Betalactam
Biocide
Glycopeptide
Lincosamide
Macrolide
Metal
Phenicol
Polymyxin
Quinolone
Sulphonamide
Tetracycline
Trimethoprim
4.9
4.37
3.84
3.31
2.78
2.25
1.71
1.18
0.65
0.12
-0.41
Fruit/Vegetables
Aminoglycoside
Betalactam
Biocide
Glycopeptide
Lincosamide
Macrolide
Metal
Phenicol
Polymyxin
Quinolone
Sulphonamide
Tetracycline
Trimethoprim
1.65
1.34
1.04
0.73
0.42
0.12
-0.19
-0.49
-0.8
-1.11
-1.41
Dairy
Aminoglycoside
Betalactam
Biocide
Glycopeptide
Lincosamide
Macrolide
Metal
Phenicol
Polymyxin
Quinolone
Sulphonamide
Tetracycline
Trimethoprim
3.64
3.07
2.5
1.93
1.35
0.78
0.21
-0.36
-0.94
-1.51
-2.08

Providencia

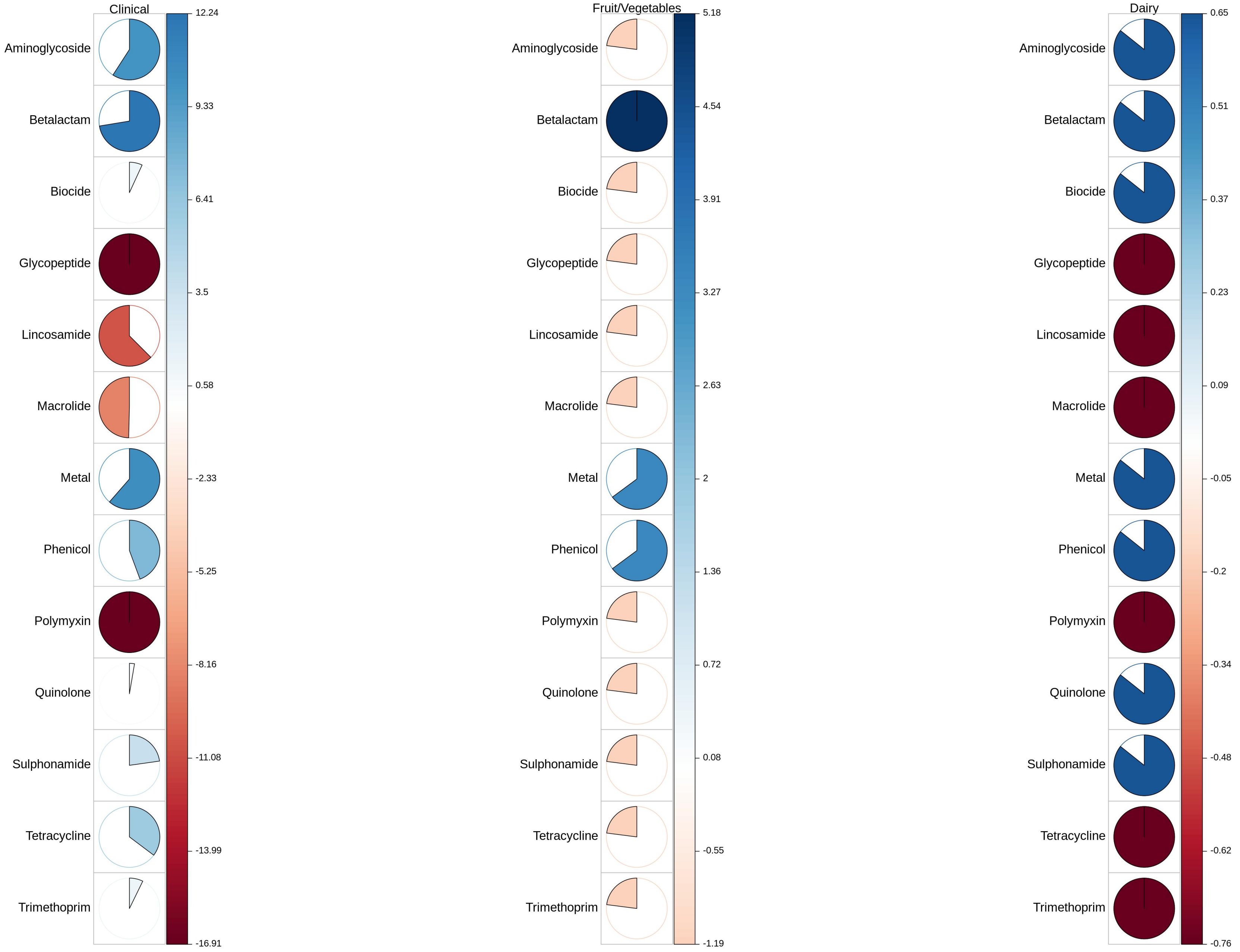

Pseudomonas

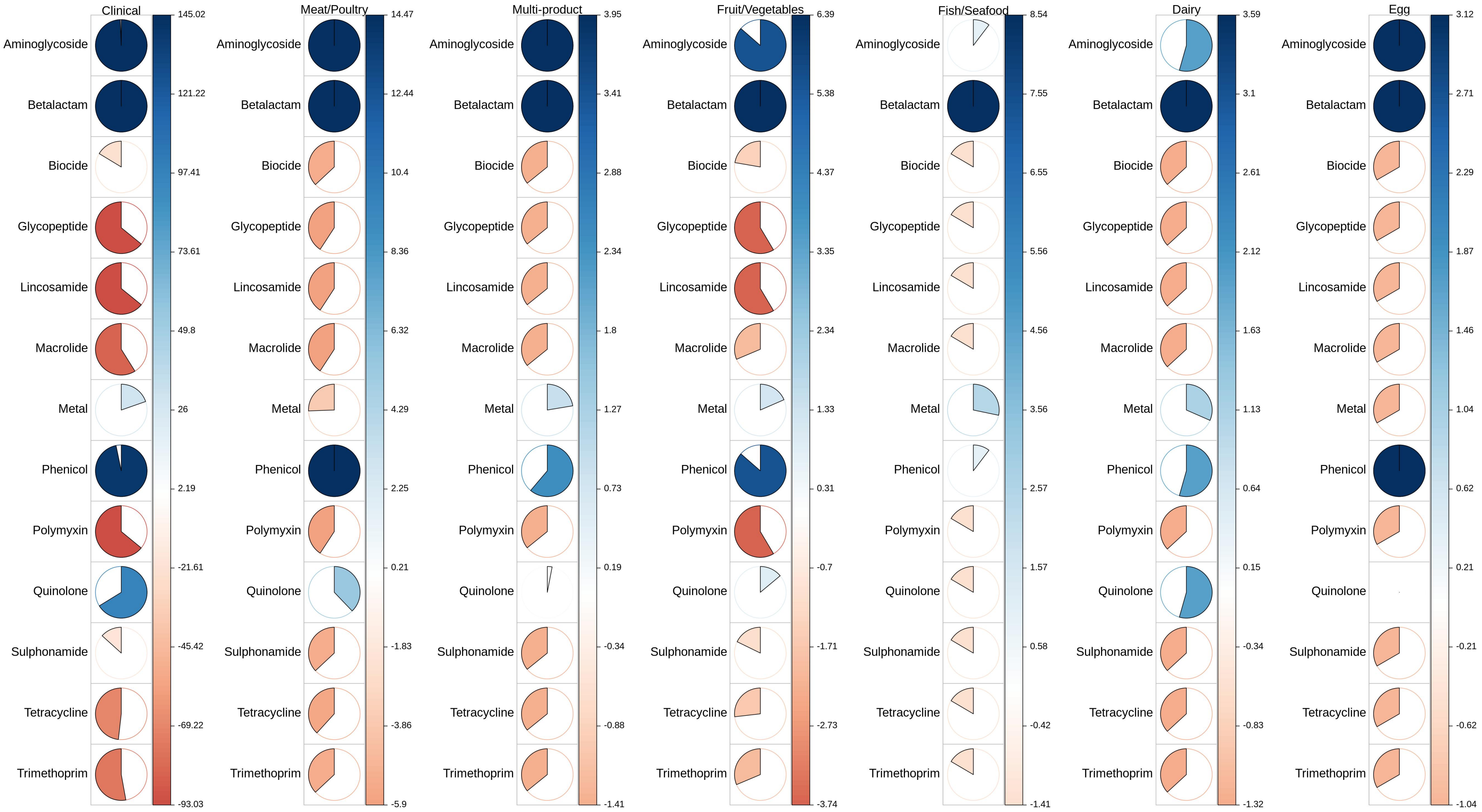

Salmonella

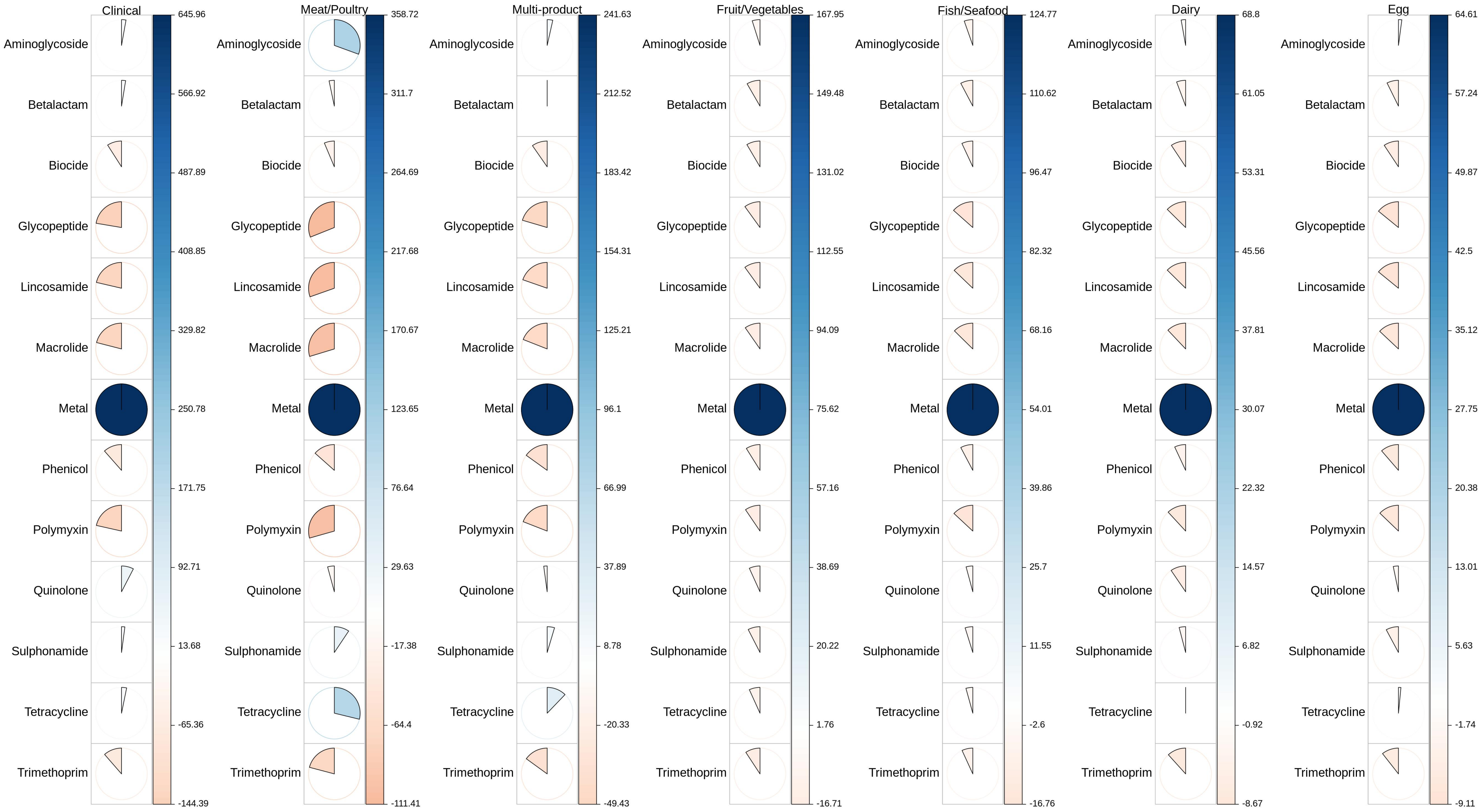

Clinical
Meat/Poultry
Multi-product
Fruit/Vegetables
Fish/Seafood
Dairy
Egg
Aminoglycoside
Betalactam
Biocide
Glycopeptide
Lincosamide
Macrolide
Metal
Phenicol
Polymyxin
Quinolone
Sulphonamide
Tetracycline
Trimethoprim
645.96
566.92
487.89
408.85
329.82
250.78
171.75
92.71
13.68
-65.36
-144.39
358.72
311.7
264.69
217.68
170.67
123.65
76.64
29.63
-17.38
-64.4
-111.41
241.63
212.52
183.42
154.31
125.21
96.1
66.99
37.89
8.78
-20.33
-49.43
167.95
149.48
131.02
112.55
94.09
75.62
57.16
38.69
20.22
1.76
-16.71
124.77
110.62
96.47
82.32
68.16
54.01
39.86
25.7
11.55
-2.6
-16.76
68.8
61.05
53.31
45.56
37.81
30.07
22.32
14.57
6.82
-0.92
-8.67
64.61
57.24
49.87
42.5
35.12
27.75
20.38
13.01
5.63
-1.74
-9.11

Serratia

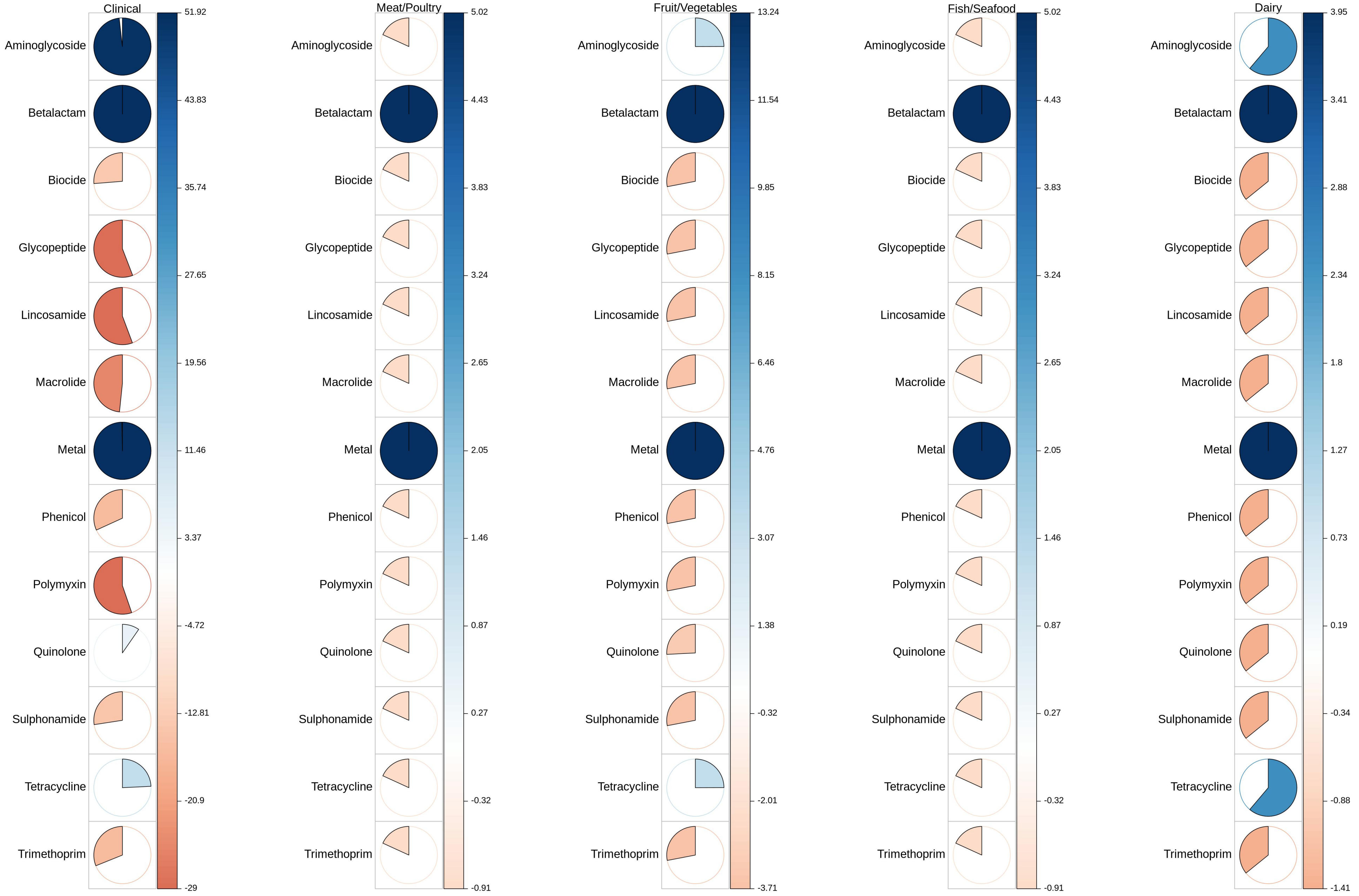

Clinical
Meat/Poultry
Fruit/Vegetables
Fish/Seafood
Dairy
Aminoglycoside
Betalactam
Biocide
Glycopeptide
Lincosamide
Macrolide
Metal
Phenicol
Polymyxin
Quinolone
Sulphonamide
Tetracycline
Trimethoprim
51.92
43.83
35.74
27.65
19.56
11.46
3.37
-4.72
-12.81
-20.9
-29
5.02
4.43
3.83
3.24
2.65
2.05
1.46
0.87
0.27
-0.32
-0.91
13.24
11.54
9.85
8.15
6.46
4.76
3.07
1.38
-0.32
-2.01
-3.71
5.02
4.43
3.83
3.24
2.65
2.05
1.46
0.87
0.27
-0.32
-0.91
3.95
3.41
2.88
2.34
1.8
1.27
0.73
0.19
-0.34
-0.88
-1.41

Shewanella algae

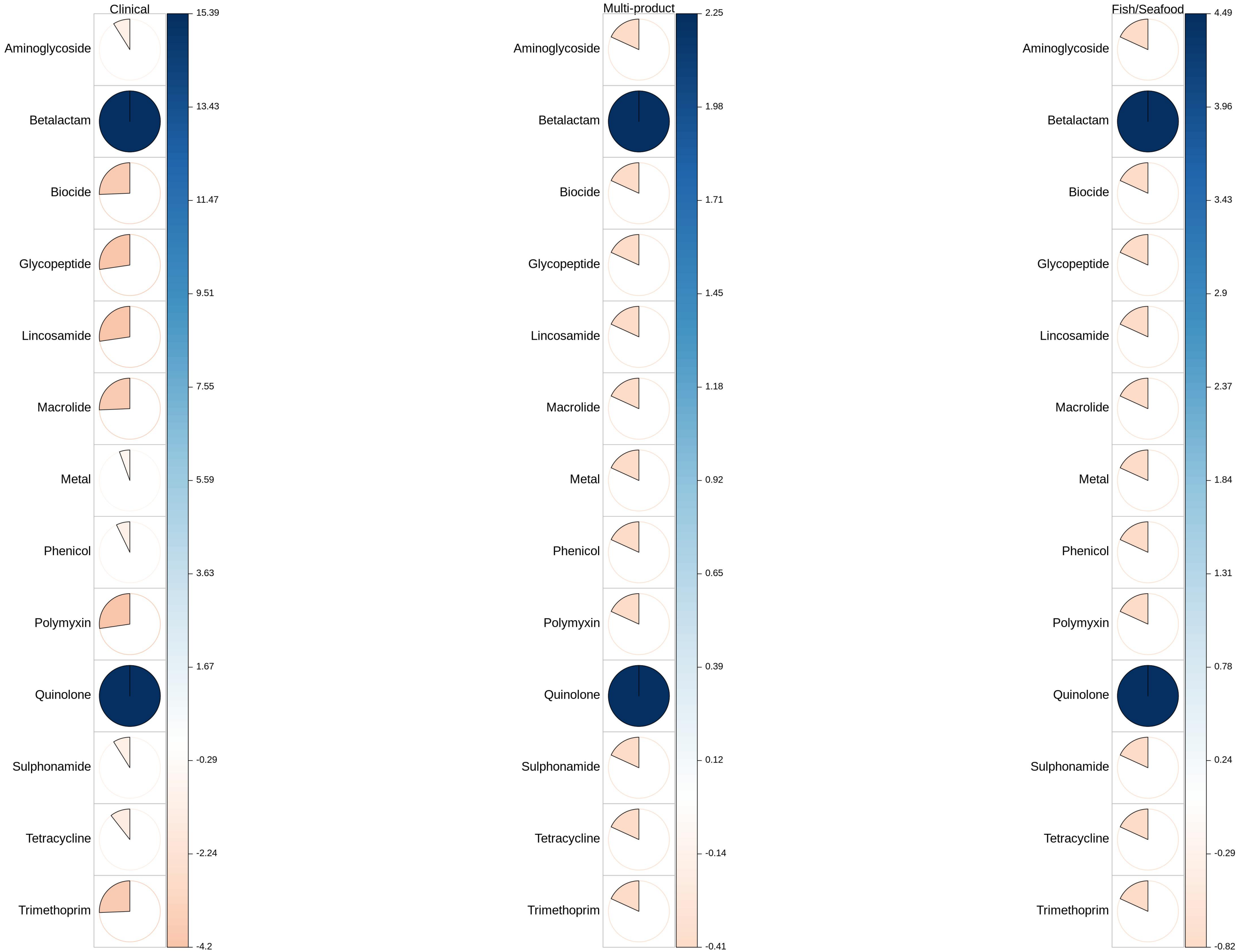

Shigella

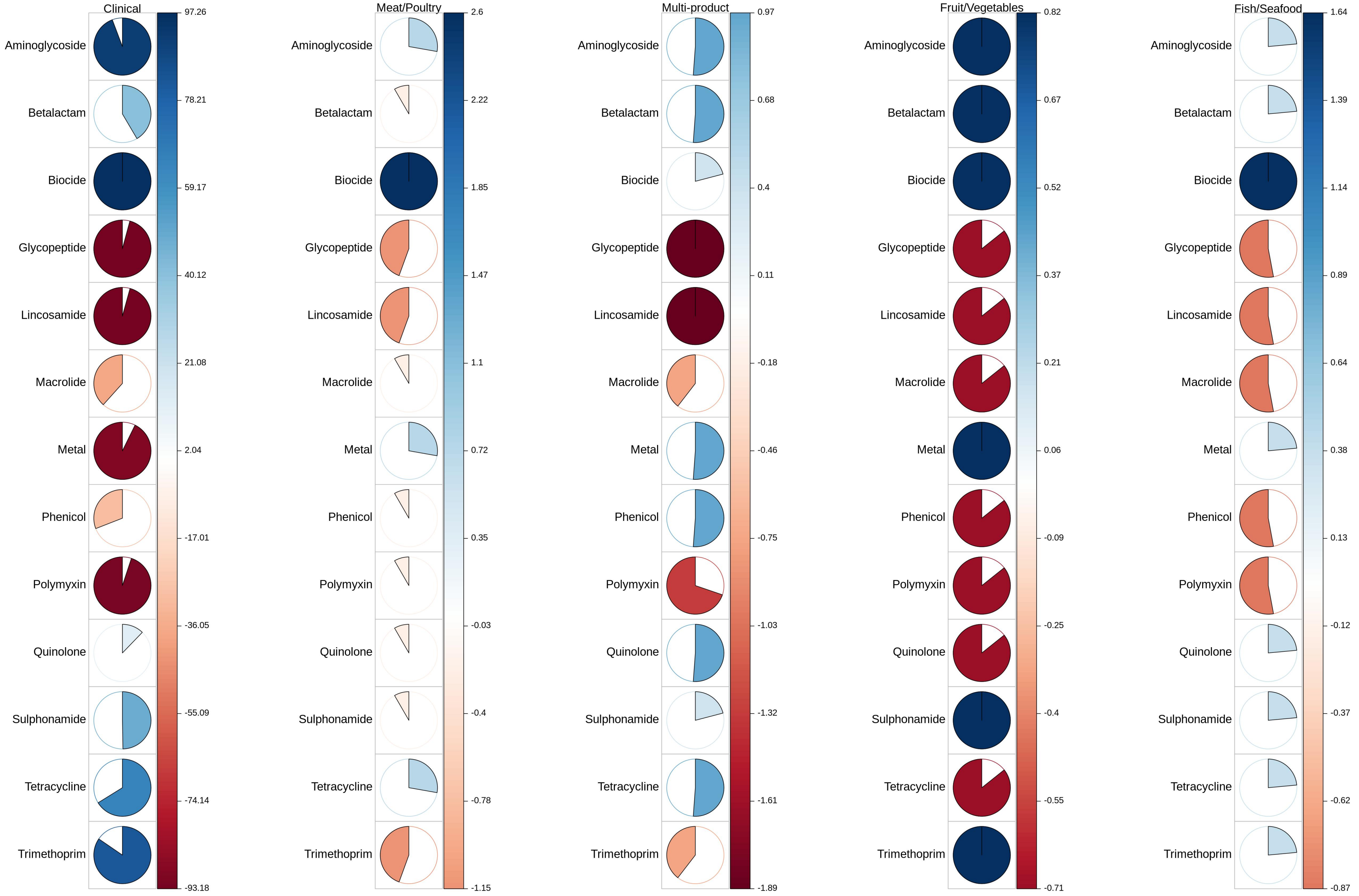
Clinical
Meat/Poultry
Multi-product
Fruit/Vegetables
Fish/Seafood
Aminoglycoside
Betalactam
Biocide
Glycopeptide
Lincosamide
Macrolide
Metal
Phenicol
Polymyxin
Quinolone
Sulphonamide
Tetracycline
Trimethoprim
97.26
78.21
59.17
40.12
21.08
2.04
-17.01
-36.05
-55.09
-74.14
-93.18
2.6
2.22
1.85
1.47
1.1
0.72
0.35
-0.03
-0.4
-0.78
-1.15
0.97
0.68
0.4
0.11
-0.18
-0.46
-0.75
-1.03
-1.32
-1.61
-1.89
0.82
0.67
0.52
0.37
0.21
0.06
-0.09
-0.25
-0.4
-0.55
-0.71
1.64
1.39
1.14
0.89
0.64
0.38
0.13
-0.12
-0.37
-0.62
-0.87

Staphylococcus

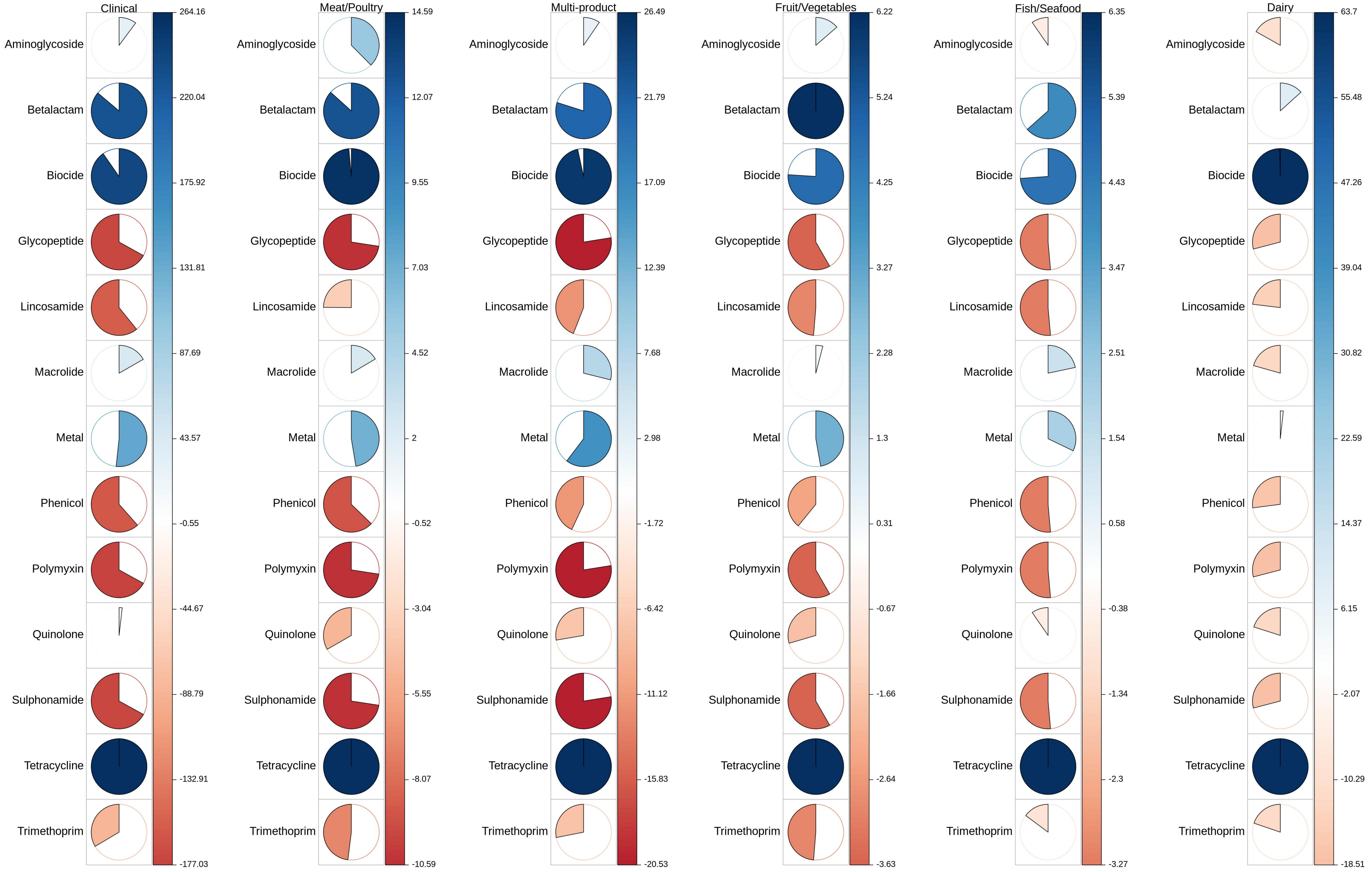

Stenotrophomonas maltophilia

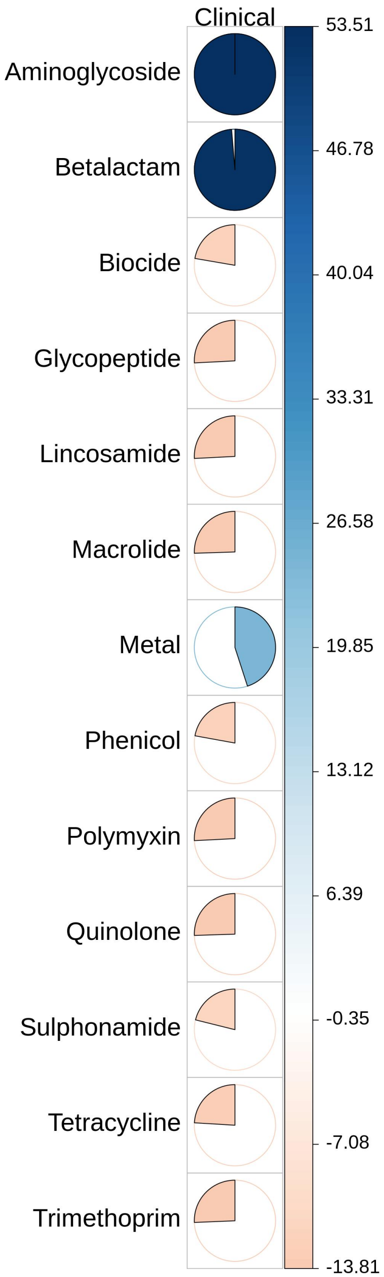

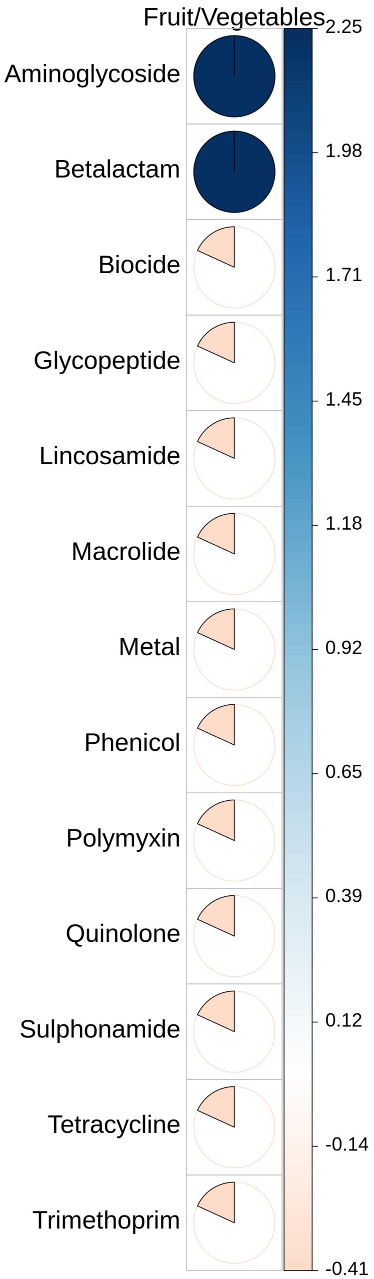

Vibrio

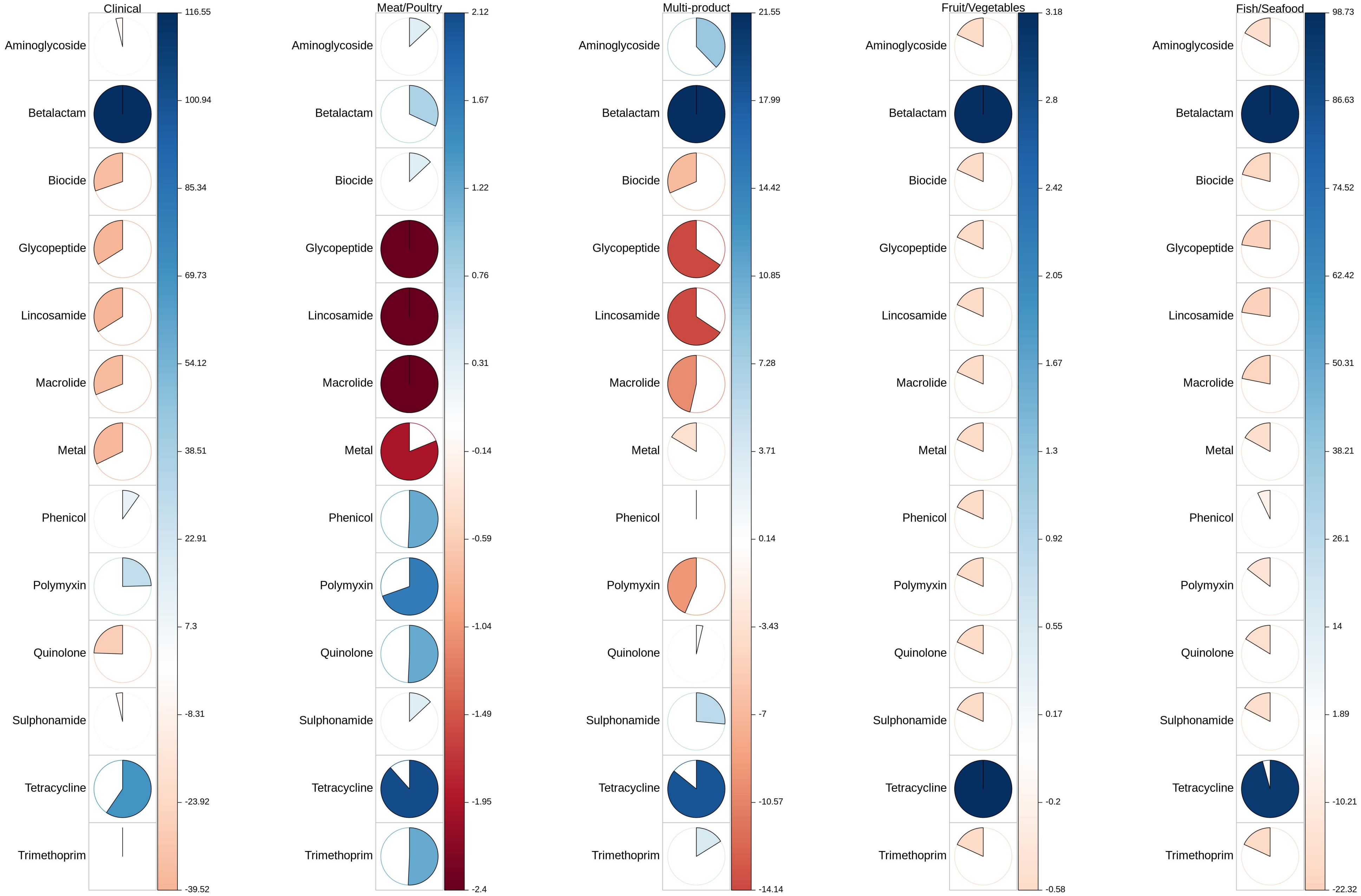
Clinical
Meat/Poultry
Multi-product
Fruit/Vegetables
Fish/Seafood
Aminoglycoside
Betalactam
Biocide
Glycopeptide
Lincosamide
Macrolide
Metal
Phenicol
Polymyxin
Quinolone
Sulphonamide
Tetracycline
Trimethoprim
116.55
100.94
85.34
69.73
54.12
38.51
22.91
7.3
-8.31
-23.92
-39.52
2.12
1.67
1.22
0.76
0.31
-0.14
-0.59
-1.04
-1.49
-1.95
-2.4
21.55
17.99
14.42
10.85
7.28
3.71
0.14
-3.43
-7
-10.57
-14.14
3.18
2.8
2.42
2.05
1.67
1.3
0.92
0.55
0.17
-0.2
-0.58
98.73
86.63
74.52
62.42
50.31
38.21
26.1
14
1.89
-10.21
-22.32
