# Supplementary material for: Analysis of Antimicrobial Resistance in Bacterial Pathogens Recovered from Food and Human Sources: Insights from 639,087 Bacterial Whole-Genome Sequences in the NCBI Pathogen Detection Database
Source: Microorganisms. 2024 Mar 30;12(4):709. doi: 10.3390/microorganisms12040709 (PMC11051753; doi:10.3390/microorganisms12040709)
Supplement: Supplementary file 1 [file microorganisms-12-00709-s001.zip › Supplementary File S4.pdf]

Supplementary File S4

The following pages contain figures for Beta-lactam resistance gene families observed in bacterial species of select genera (title at top left of each page). For each species listed on the y-axis, percentage of isolates from each source (differentiated by colour) encoding each respective Beta-lactam gene-family (top panel headings) is displayed along the x-axis. Sizing of points corresponds to the total number of isolates of that species for which isolation source could be identified (No. of isolates). Metadata files for whole-genome sequence data were downloaded from the NCBI Pathogen Detection database. Datasets were analysed and source categories were assigned based on reported sequence submission information. Data was excluded from analysis where source information was missing or incomplete (see methods). For each gene family displayed, all alleles in the AMRFinderPlus database are included.

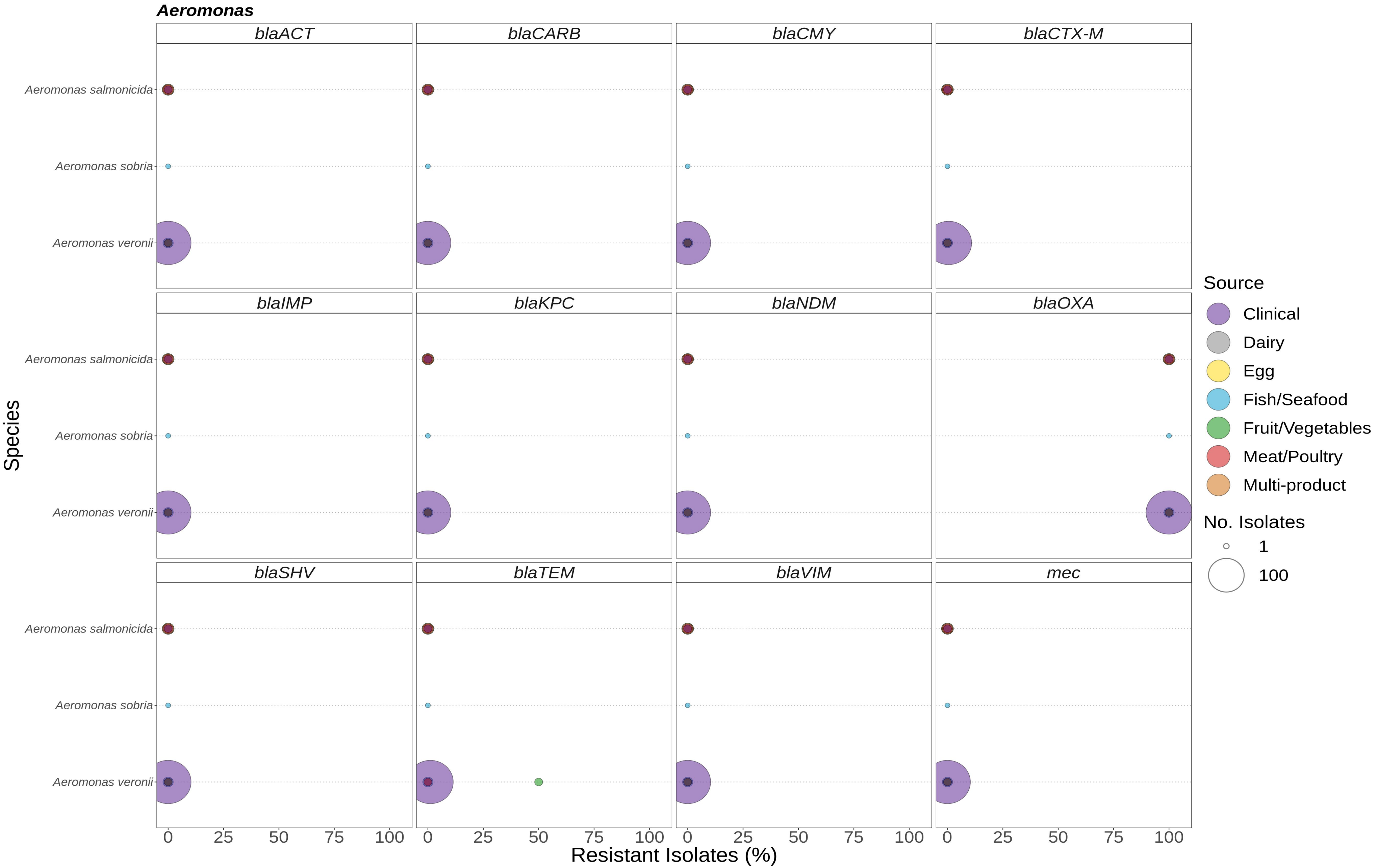

Aeromonas
blaACT
blaCARB
blaCMY
blaCTX-M
blaIMP
blaKPC
blaNDM
blaOXA
blaSHV
blaTEM
blaVIM
mec
Aeromonas salmonicida
Aeromonas sobria
Aeromonas veronii
Species
0
25
50
75
100
Resistant Isolates (%)
Source
Clinical
Dairy
Egg
Fish/Seafood
Fruit/Vegetables
Meat/Poultry
Multi-product
No. Isolates
1
100

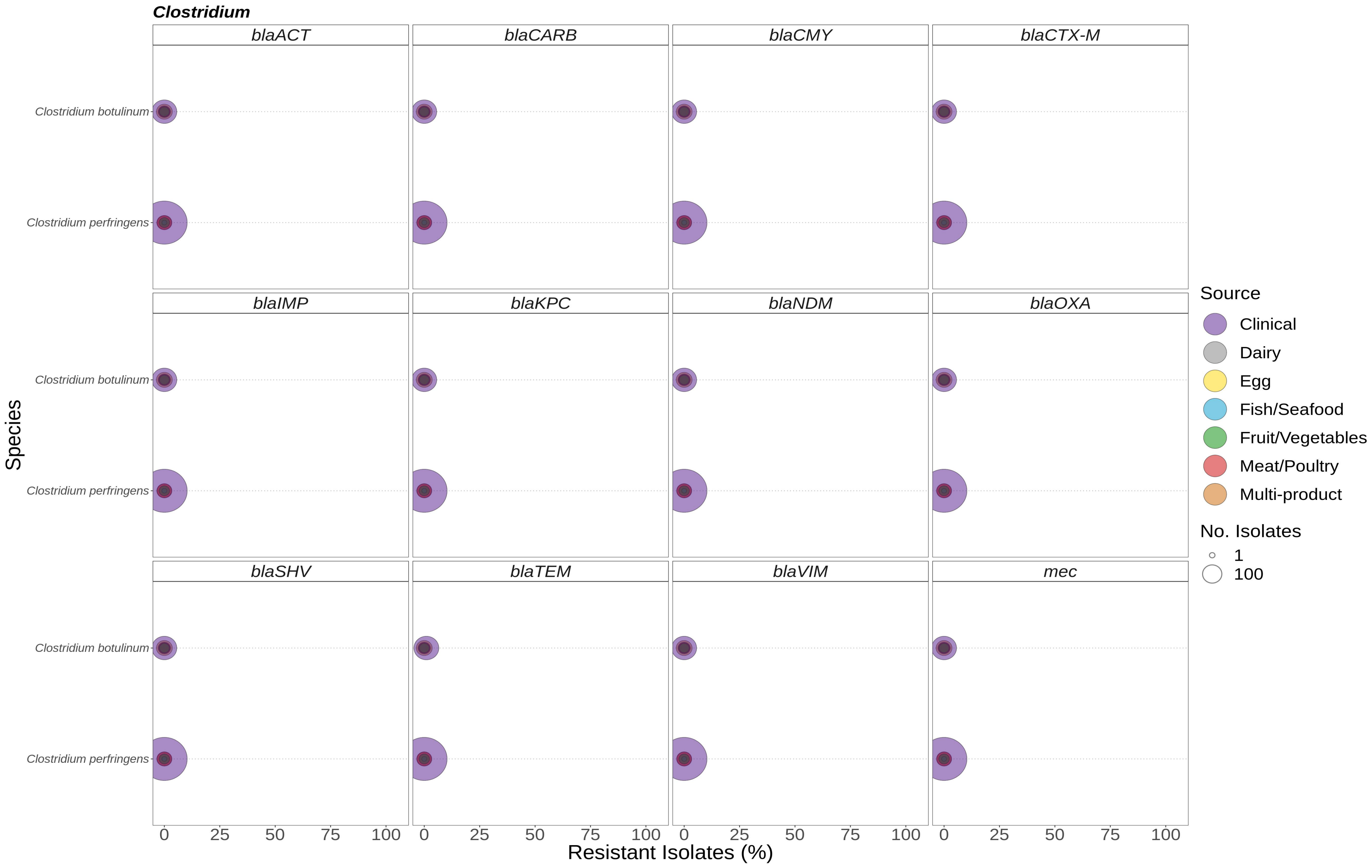

Clostridium
blaACT
blaCARB
blaCMY
blaCTX-M
blaIMP
blaKPC
blaNDM
blaOXA
blaSHV
blaTEM
blaVIM
mec
Clostridium botulinum
Clostridium perfringens
Species
0
25
50
75
100
Resistant Isolates (%)
Source
Clinical
Dairy
Egg
Fish/Seafood
Fruit/Vegetables
Meat/Poultry
Multi-product
No. Isolates
1
100

Enterobacter

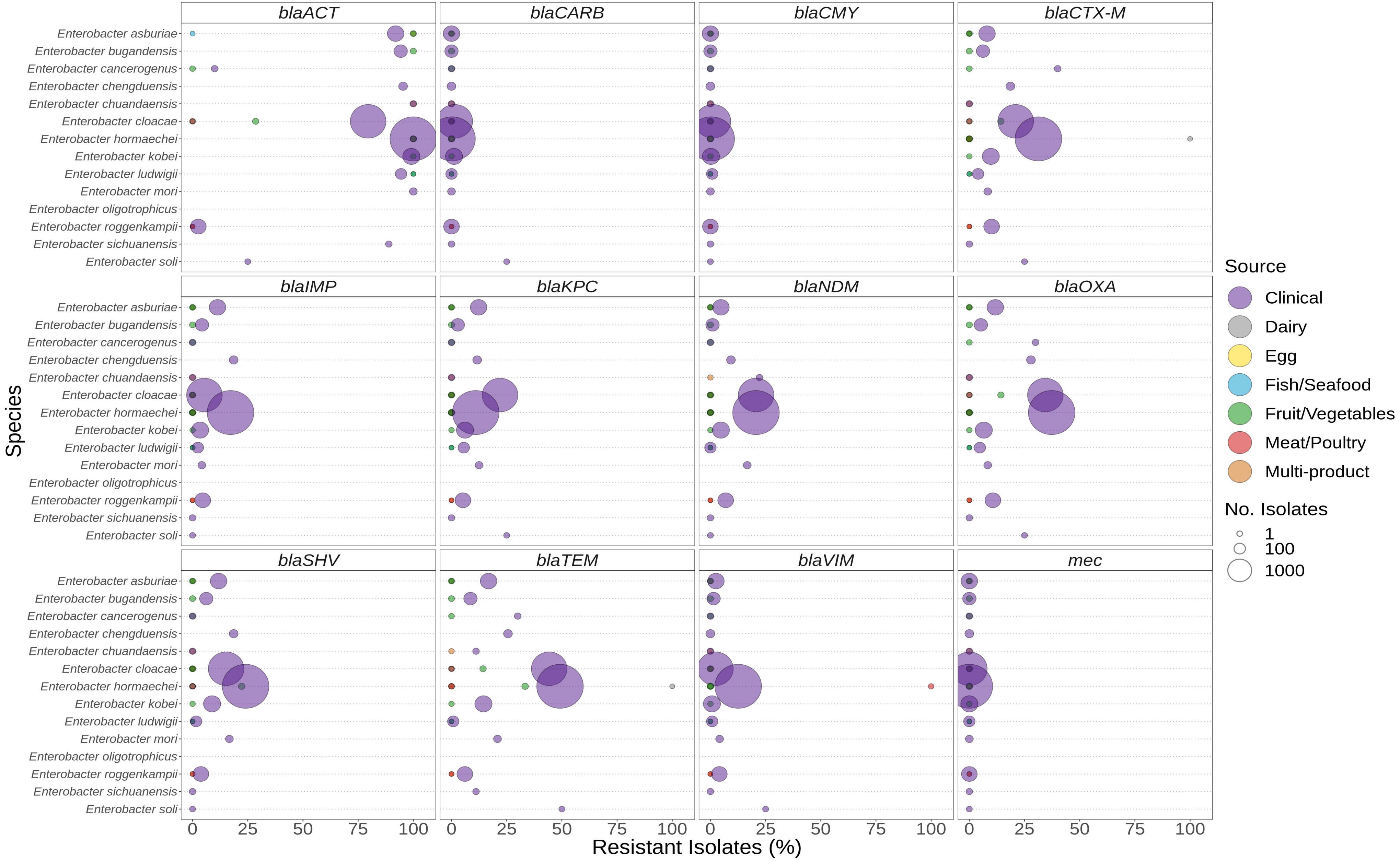

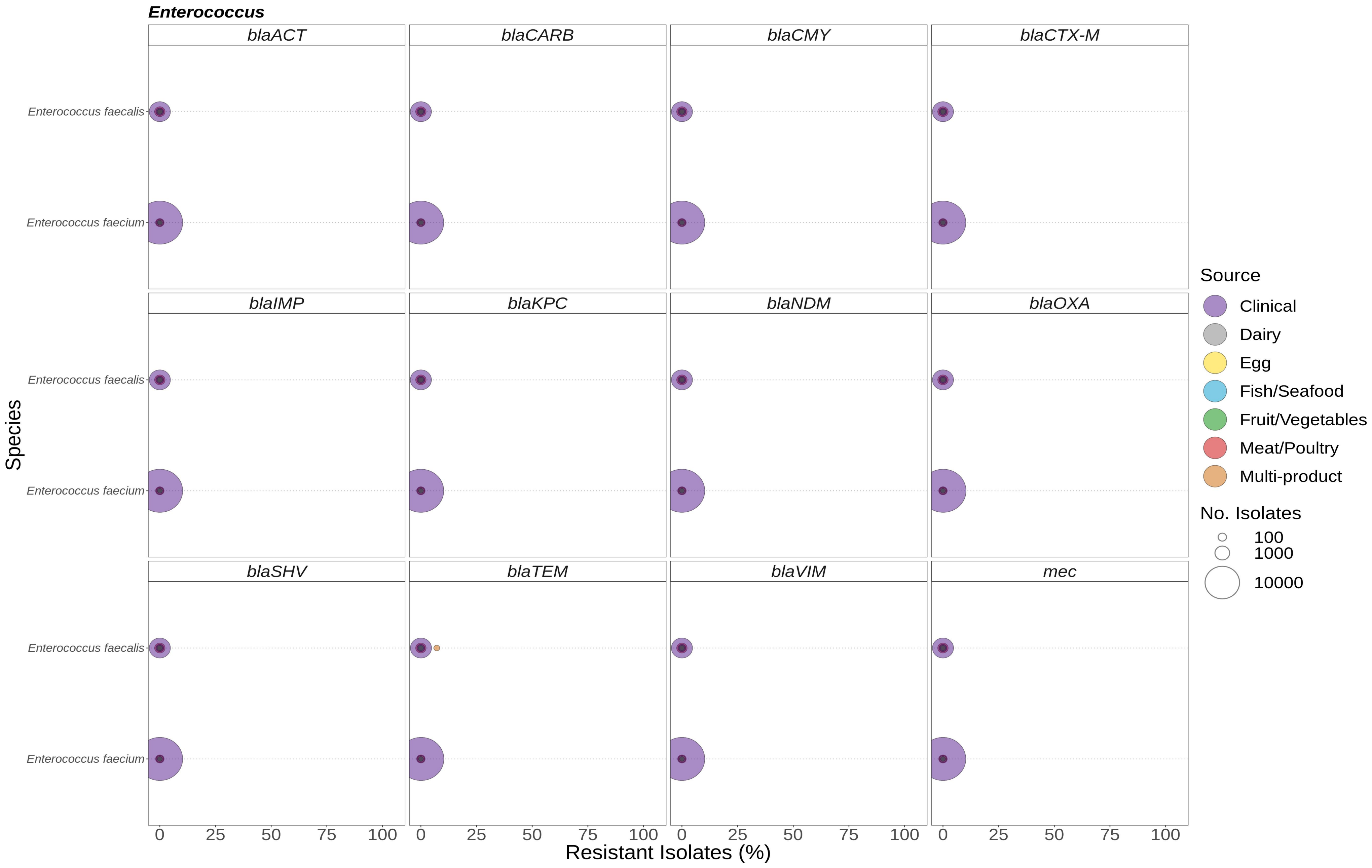
Enterococcus
blaACT
blaCARB
blaCMY
blaCTX-M
blaIMP
blaKPC
blaNDM
blaOXA
blaSHV
blaTEM
blaVIM
mec
Enterococcus faecalis
Enterococcus faecium
Species
0
25
50
75
100
Resistant Isolates (%)
Source
Clinical
Dairy
Egg
Fish/Seafood
Fruit/Vegetables
Meat/Poultry
Multi-product
No. Isolates
100
1000
10000

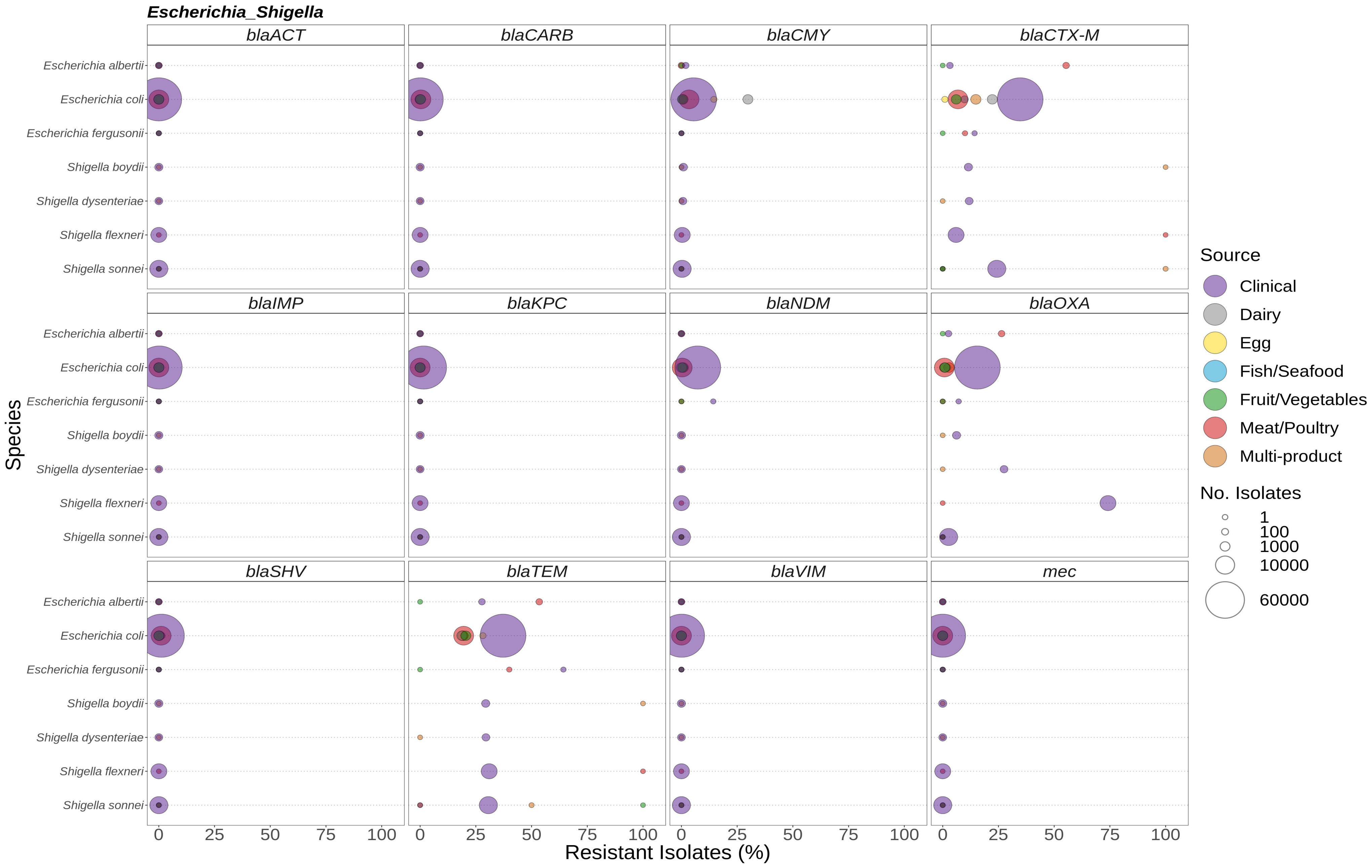
Escherichia_Shigella
blaACT
blaCARB
blaCMY
blaCTX-M
blaIMP
blaKPC
blaNDM
blaOXA
blaSHV
blaTEM
blaVIM
mec
Escherichia albertii
Escherichia coli
Escherichia fergusonii
Shigella boydii
Shigella dysenteriae
Shigella flexneri
Shigella sonnei
Species
0
25
50
75
100
Resistant Isolates (%)
Source
Clinical
Dairy
Egg
Fish/Seafood
Fruit/Vegetables
Meat/Poultry
Multi-product
No. Isolates
1
100
1000
10000
60000

Klebsiella

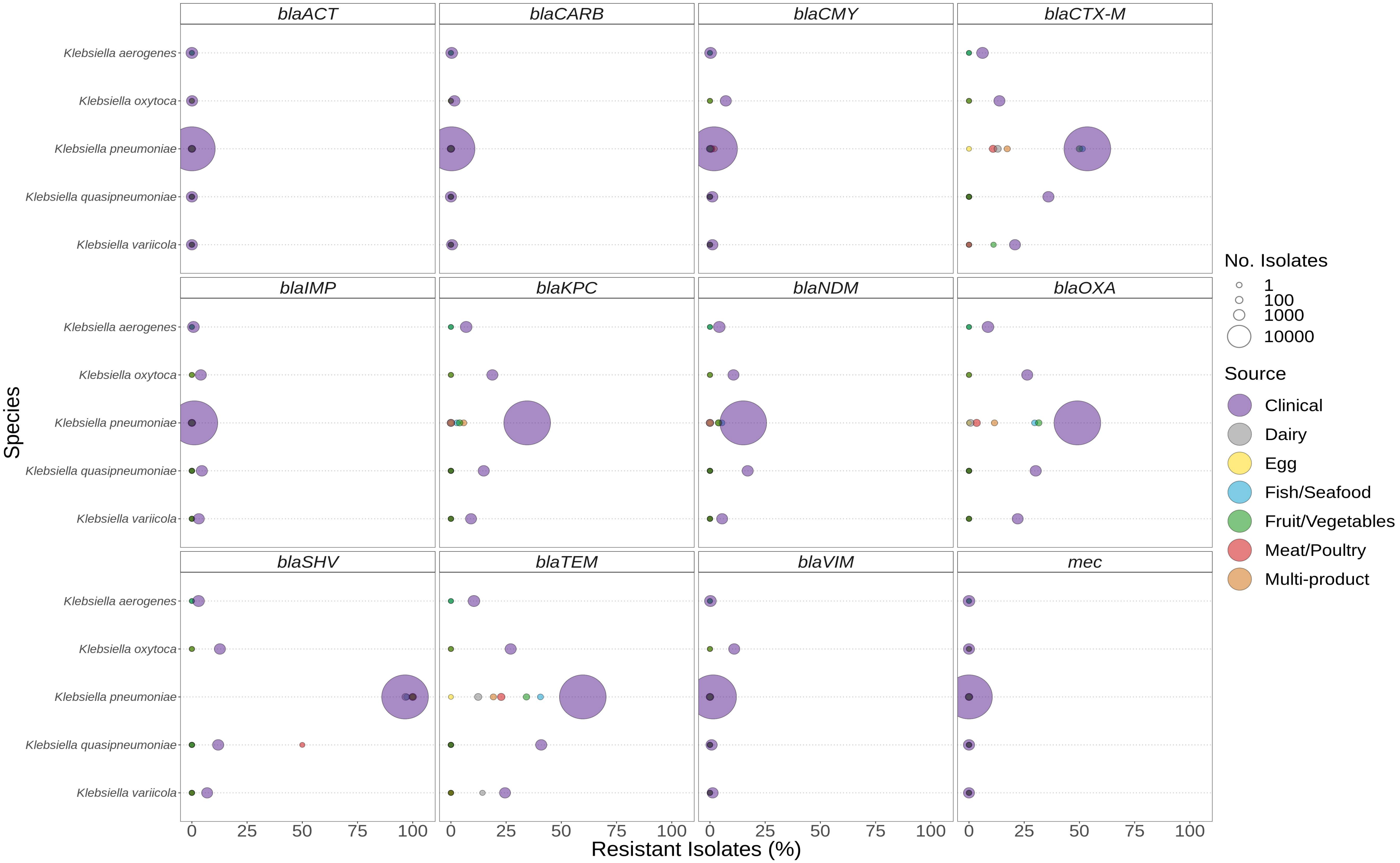
blaACT
blaCARB
blaCMY
blaCTX-M
blaIMP
blaKPC
blaNDM
blaOXA
blaSHV
blaTEM
blaVIM
mec
Klebsiella aerogenes
Klebsiella oxytoca
Klebsiella pneumoniae
Klebsiella quasipneumoniae
Klebsiella variicola
Species
0
25
50
75
100
Resistant Isolates (%)
No. Isolates
1
100
1000
10000
Source
Clinical
Dairy
Egg
Fish/Seafood
Fruit/Vegetables
Meat/Poultry
Multi-product

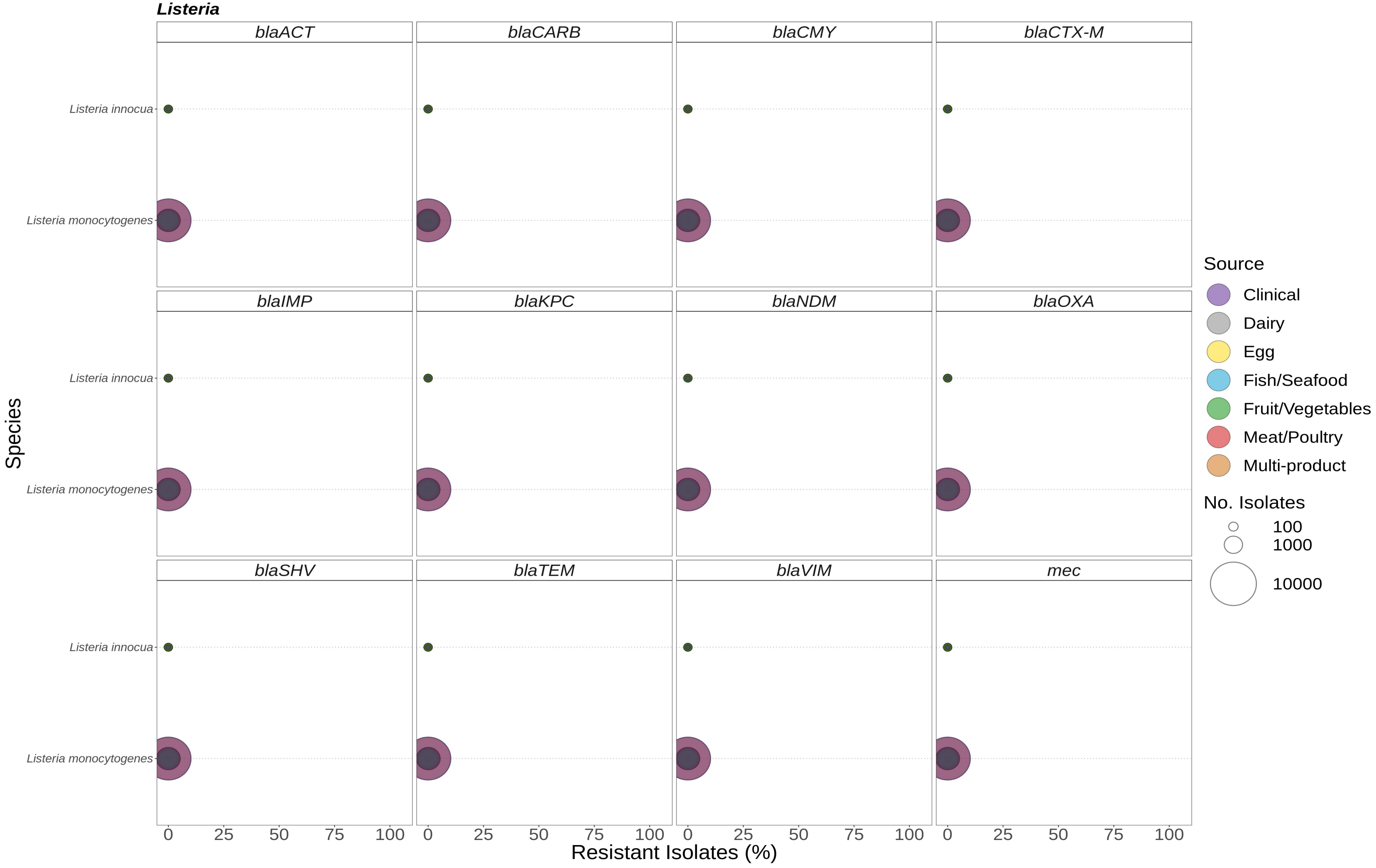
Listeria
blaACT
blaCARB
blaCMY
blaCTX-M
blaIMP
blaKPC
blaNDM
blaOXA
blaSHV
blaTEM
blaVIM
mec
Listeria innocua
Listeria monocytogenes
Species
0 25 50 75 100
Resistant Isolates (%)
Source
Clinical
Dairy
Egg
Fish/Seafood
Fruit/Vegetables
Meat/Poultry
Multi-product
No. Isolates
100
1000
10000

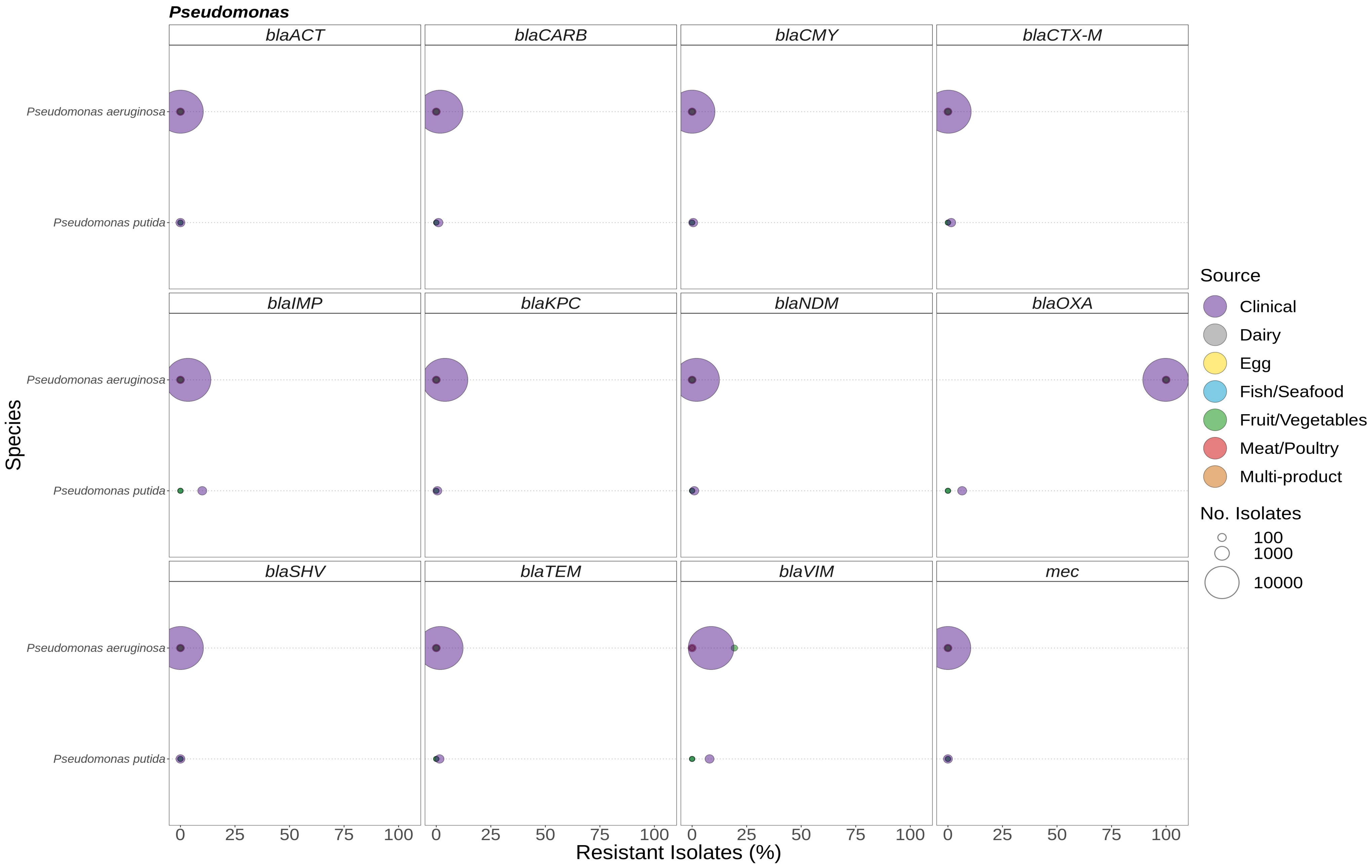

Pseudomonas
blaACT
blaCARB
blaCMY
blaCTX-M
blaIMP
blaKPC
blaNDM
blaOXA
blaSHV
blaTEM
blaVIM
mec
Pseudomonas aeruginosa
Pseudomonas putida
Species
Resistant Isolates (%)
0
25
50
75
100
Source
Clinical
Dairy
Egg
Fish/Seafood
Fruit/Vegetables
Meat/Poultry
Multi-product
No. Isolates
100
1000
10000

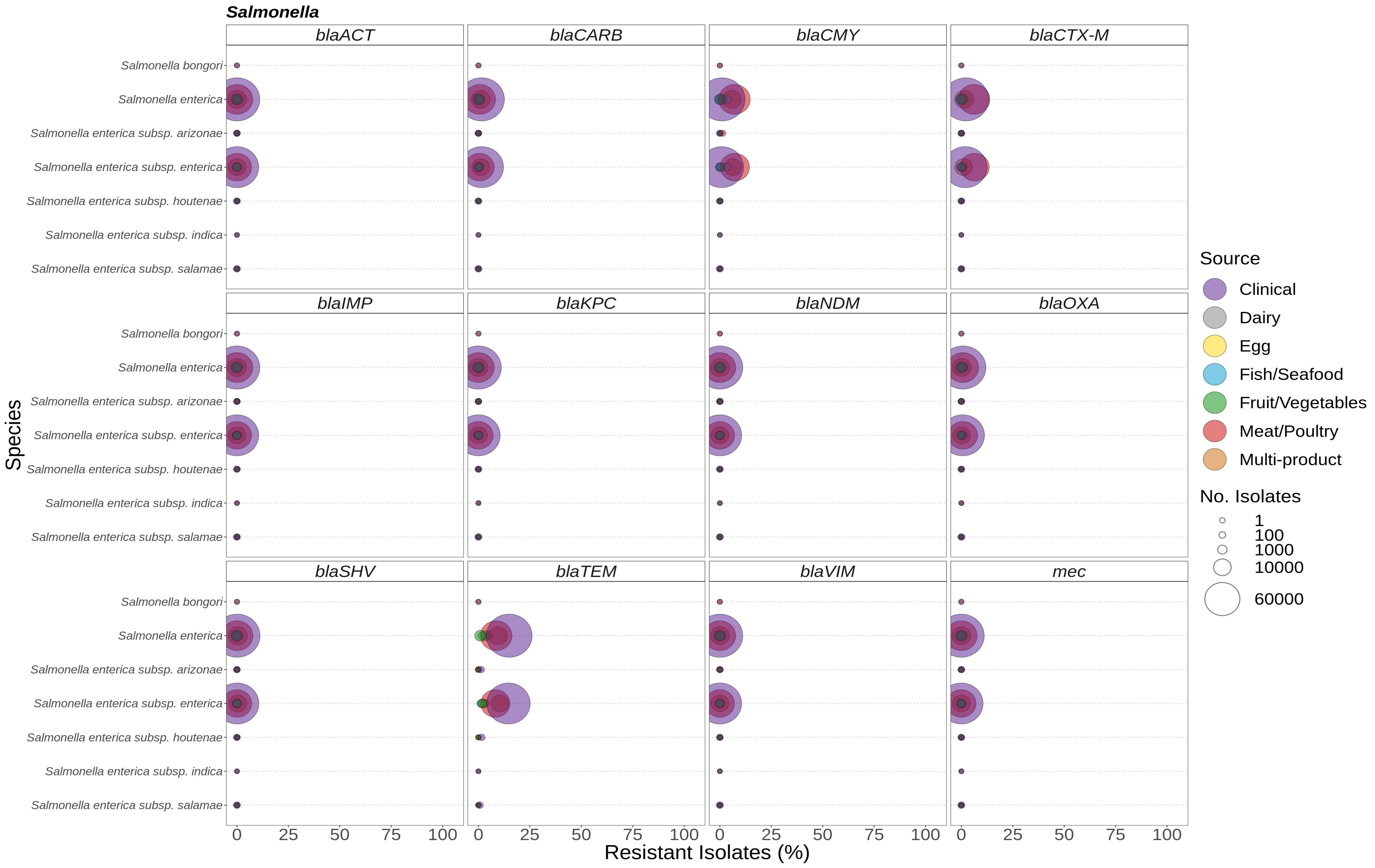
Salmonella
blaACT
blaCARB
blaCMY
blaCTX-M
blaIMP
blaKPC
blaNDM
blaOXA
blaSHV
blaTEM
blaVIM
mec
Salmonella bongori
Salmonella enterica
Salmonella enterica subsp. arizonae
Salmonella enterica subsp. enterica
Salmonella enterica subsp. houtenae
Salmonella enterica subsp. indica
Salmonella enterica subsp. salamae
Species
0
25
50
75
100
Resistant Isolates (%)
Source
Clinical
Dairy
Egg
Fish/Seafood
Fruit/Vegetables
Meat/Poultry
Multi-product
No. Isolates
1
100
1000
10000
60000

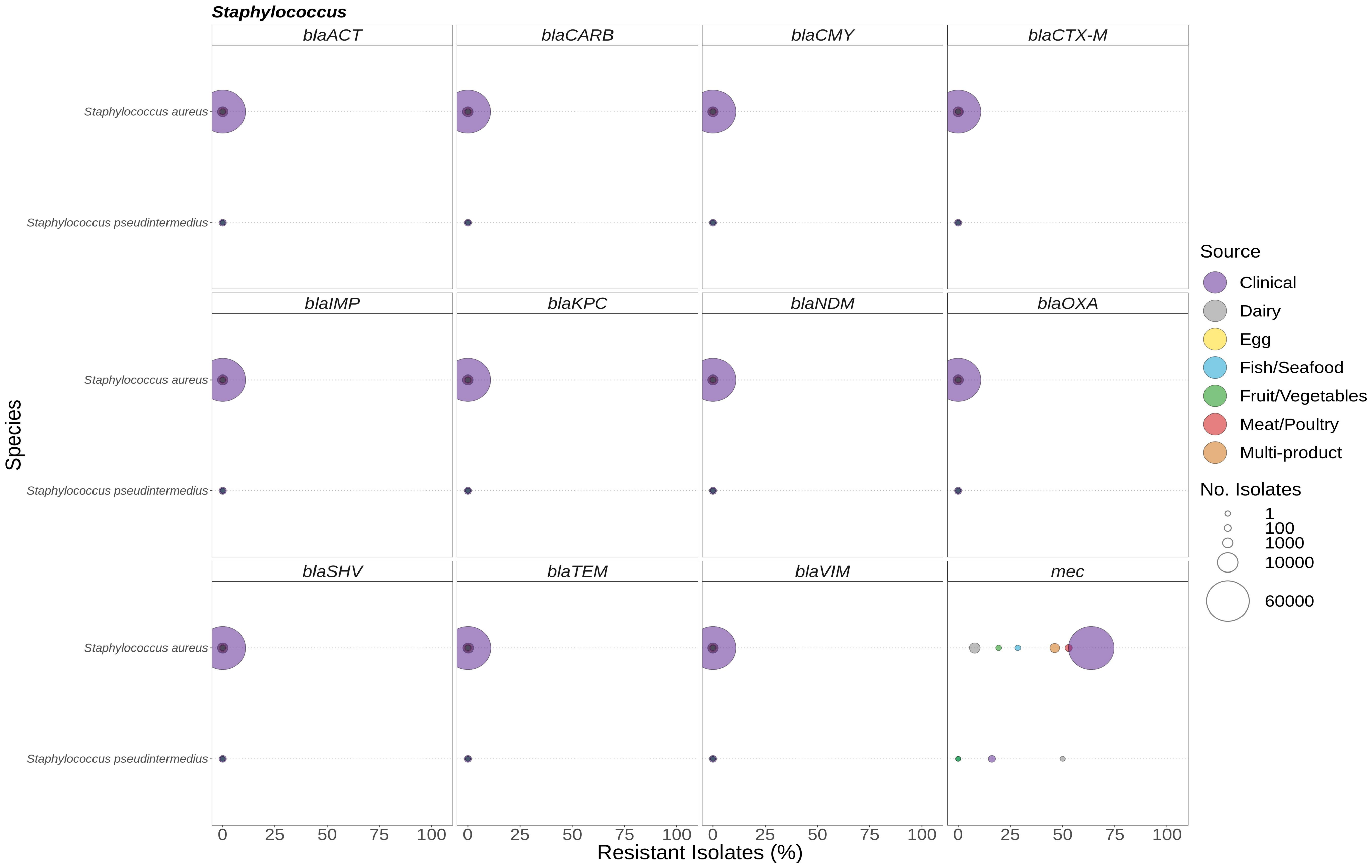
Staphylococcus
blaACT
blaCARB
blaCMY
blaCTX-M
blaIMP
blaKPC
blaNDM
blaOXA
blaSHV
blaTEM
blaVIM
mec
Staphylococcus aureus
Staphylococcus pseudintermedius
Species
0
25
50
75
100
Resistant Isolates (%)
Source
Clinical
Dairy
Egg
Fish/Seafood
Fruit/Vegetables
Meat/Poultry
Multi-product
No. Isolates
1
100
1000
10000
60000

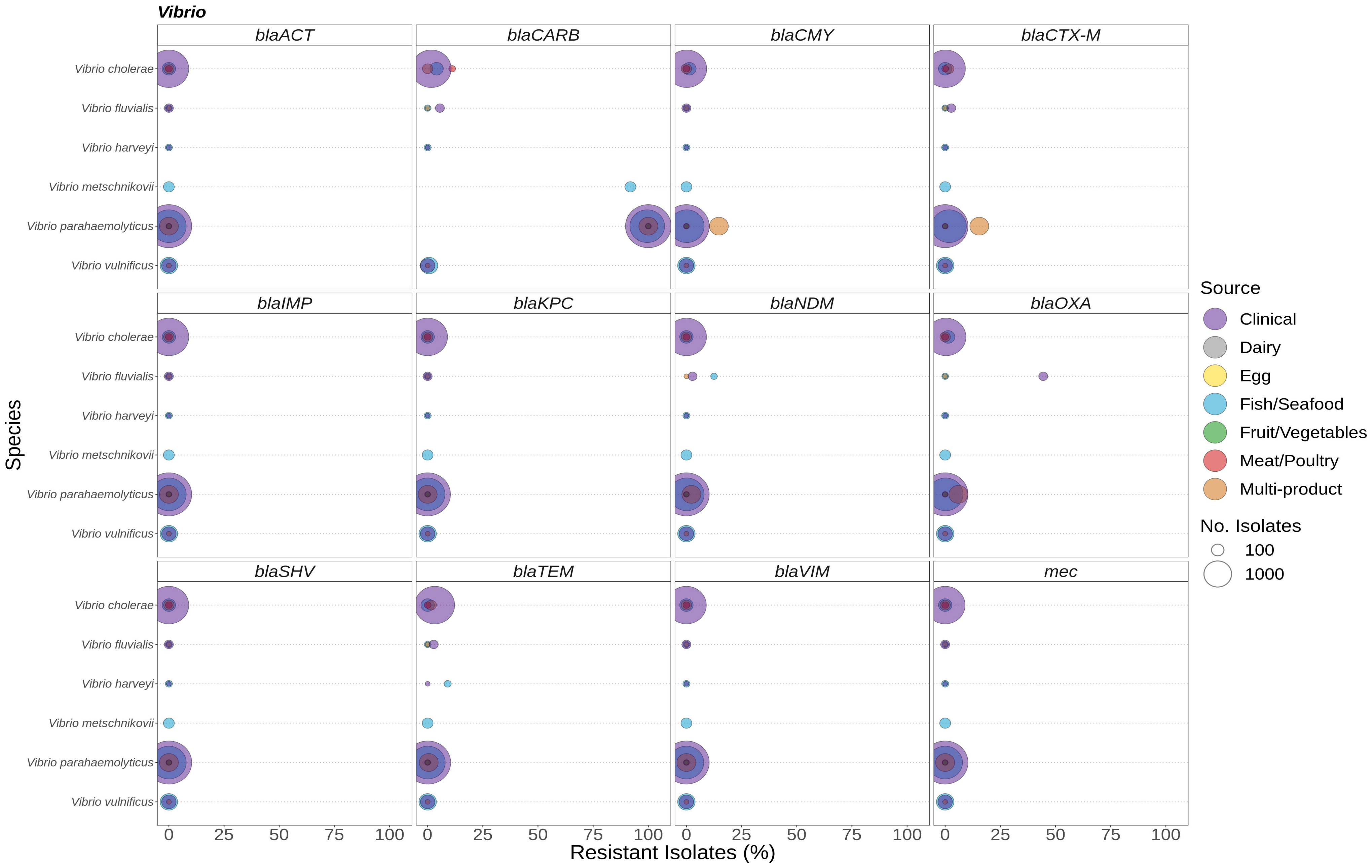

Vibrio
blaACT
blaCARB
blaCMY
blaCTX-M
blaIMP
blaKPC
blaNDM
blaOXA
blaSHV
blaTEM
blaVIM
mec
Vibrio cholerae
Vibrio fluvialis
Vibrio harveyi
Vibrio metschnikovii
Vibrio parahaemolyticus
Vibrio vulnificus
Species
0
25
50
75
100
Resistant Isolates (%)
Source
Clinical
Dairy
Egg
Fish/Seafood
Fruit/Vegetables
Meat/Poultry
Multi-product
No. Isolates
100
1000
