# Supplementary material for: Analysis of Antimicrobial Resistance in Bacterial Pathogens Recovered from Food and Human Sources: Insights from 639,087 Bacterial Whole-Genome Sequences in the NCBI Pathogen Detection Database
Source: Microorganisms. 2024 Mar 30;12(4):709. doi: 10.3390/microorganisms12040709 (PMC11051753; doi:10.3390/microorganisms12040709)

Supplementary File S5

The following pages contain figures for vancomycin resistance gene casettes observed in bacterial species of select genera (title at top left of each page). For each genus or species listed on the y-axis, percentage of isolates from each source (differentiated by colour) encoding each respective vancomycin casette (top panel headings) is displayed along the x-axis. Sizing of points corresponds to the total number of isolates of that species for which isolation source could be identified (No. of isolates). Metadata files for whole-genome sequence data were downloaded from the NCBI Pathogen Detection database. Datasets were analysed and source categories were assigned based on reported sequence submission information. Data was excluded from analysis where source information was missing or incomplete (see methods). For each gene casette displayed, all alleles in the AMRFinderPlus database are included.

All Organisms

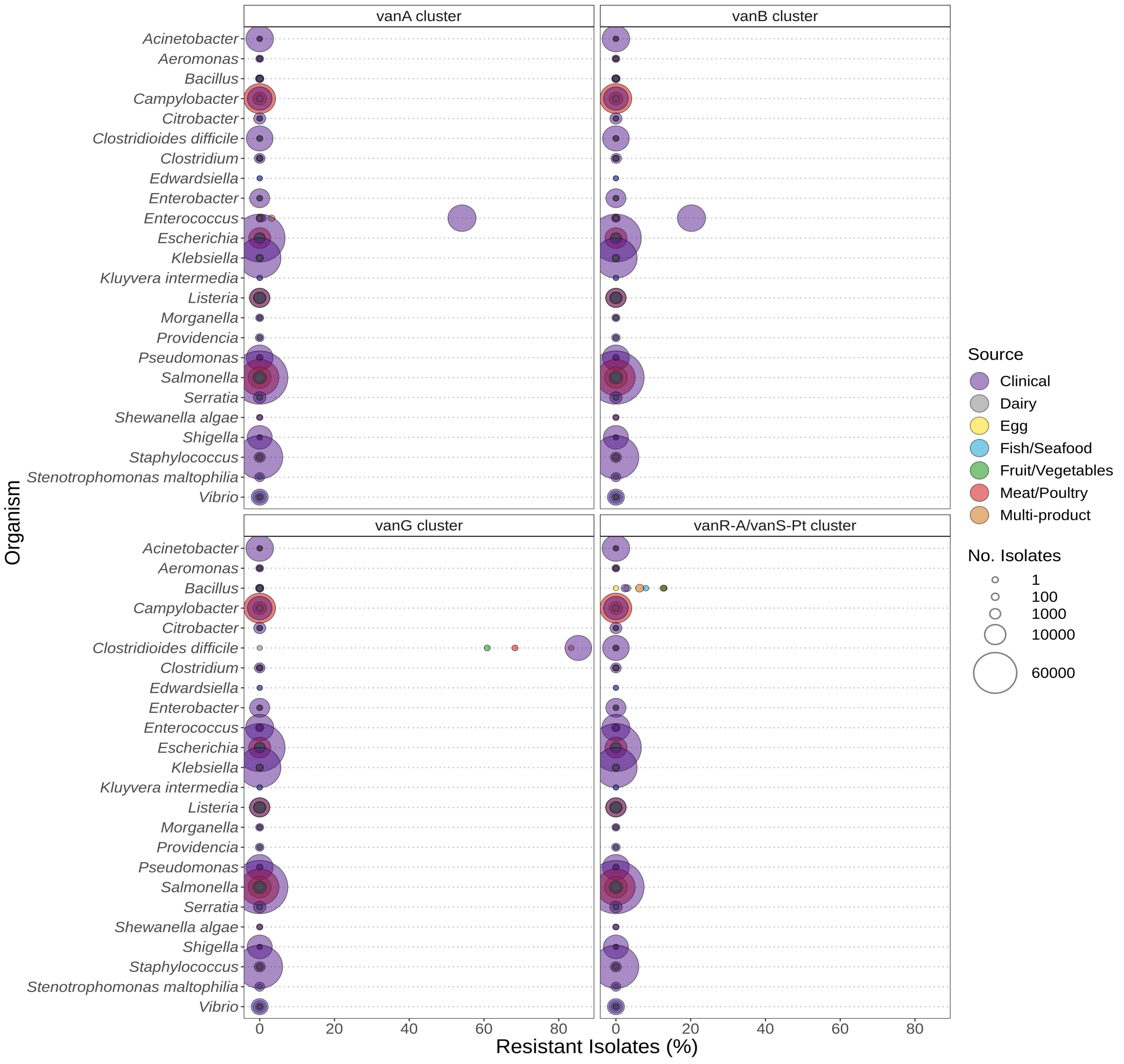

Bacillus

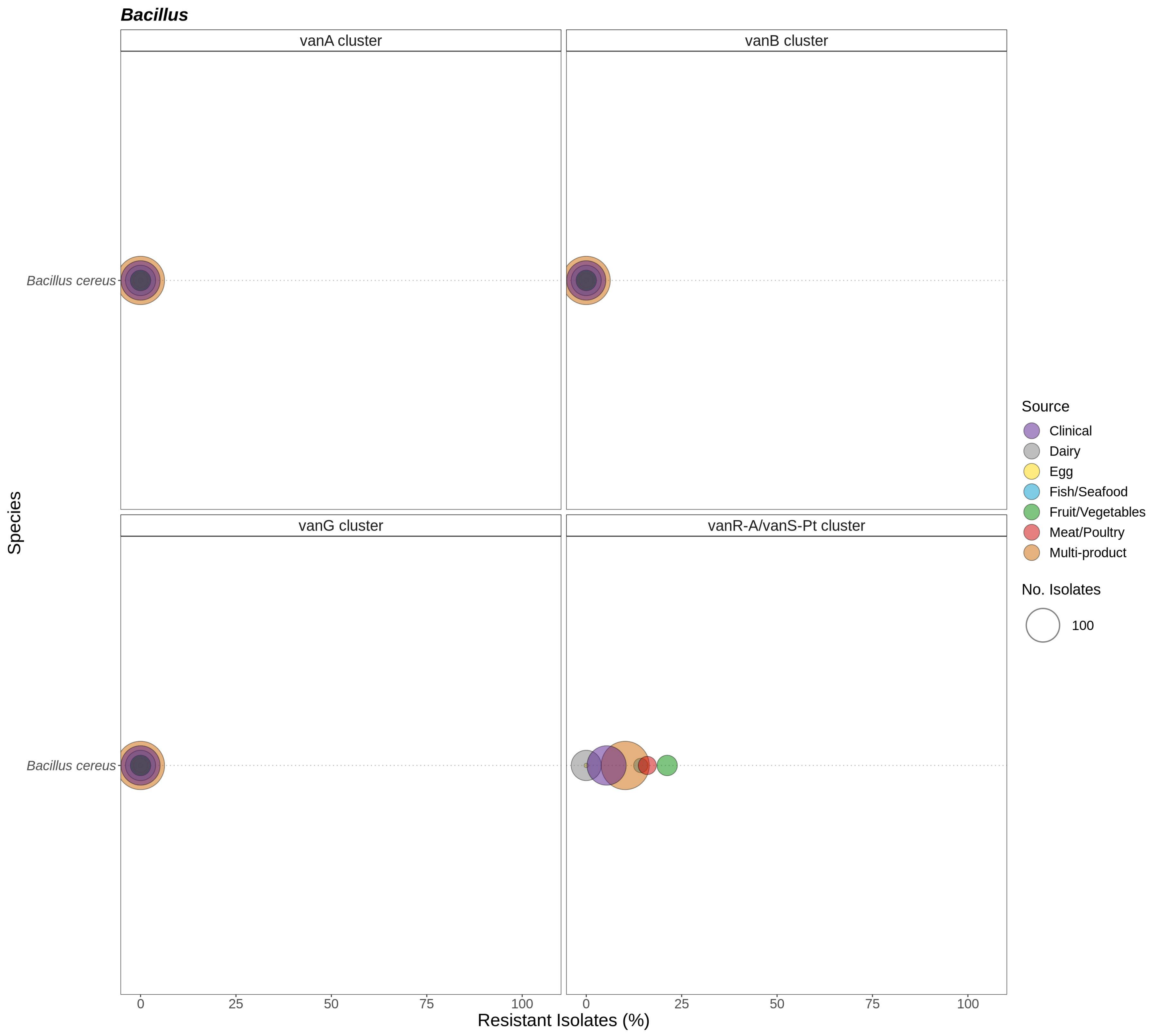

Bacillus
vanA cluster
vanB cluster
vanG cluster
vanR-A/vanS-Pt cluster
Bacillus cereus
Bacillus cereus
Species
0
25
50
75
100
Resistant Isolates (%)
Source
Clinical
Dairy
Egg
Fish/Seafood
Fruit/Vegetables
Meat/Poultry
Multi-product
No. Isolates
100

Enterococcus

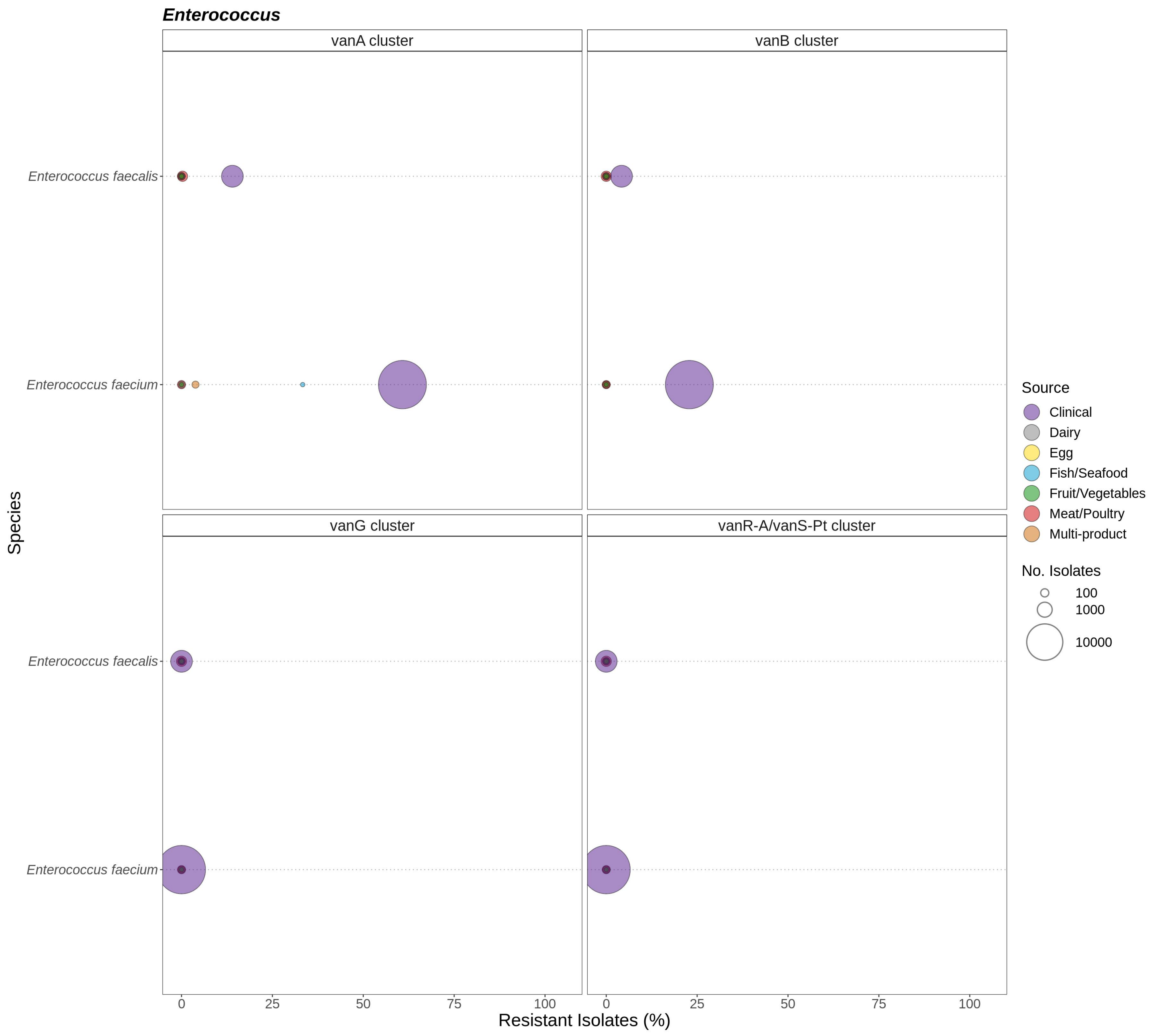

Supplement: Supplementary file 1 [file microorganisms-12-00709-s001.zip › Supplementary File S5.pdf]
