# Supplementary material for: Analysis of Antimicrobial Resistance in Bacterial Pathogens Recovered from Food and Human Sources: Insights from 639,087 Bacterial Whole-Genome Sequences in the NCBI Pathogen Detection Database
Source: Microorganisms. 2024 Mar 30;12(4):709. doi: 10.3390/microorganisms12040709 (PMC11051753; doi:10.3390/microorganisms12040709)
Supplement: Supplementary file 1 [file microorganisms-12-00709-s001.zip › Supplementary File S6.pdf]

Supplementary File S6

The following pages contain supplementary figures for statistical analysis (pairwise Fisher's exact test for proportions) comparing proportion of isolates encoding resistance to antimicrobial class (green bars) versus proportion encoding resistance to both antimicrobial class and biocides (dark red bars) for each genus (top left page title). Data is grouped by isolation source (x-axis) and antimicrobial class (top-panel headings). Significance between Antimicrobial and Antimicrobial & Biocide is displayed above grouped bars: ns = not significant; $p < 0.05$ = *; $p < 0.01$ = **; $p < 0.001$ = ***; $p < 0.0001$ = ****. The total number of isolates (n) from each source (x-axis) is listed above grouped bars in parentheses.

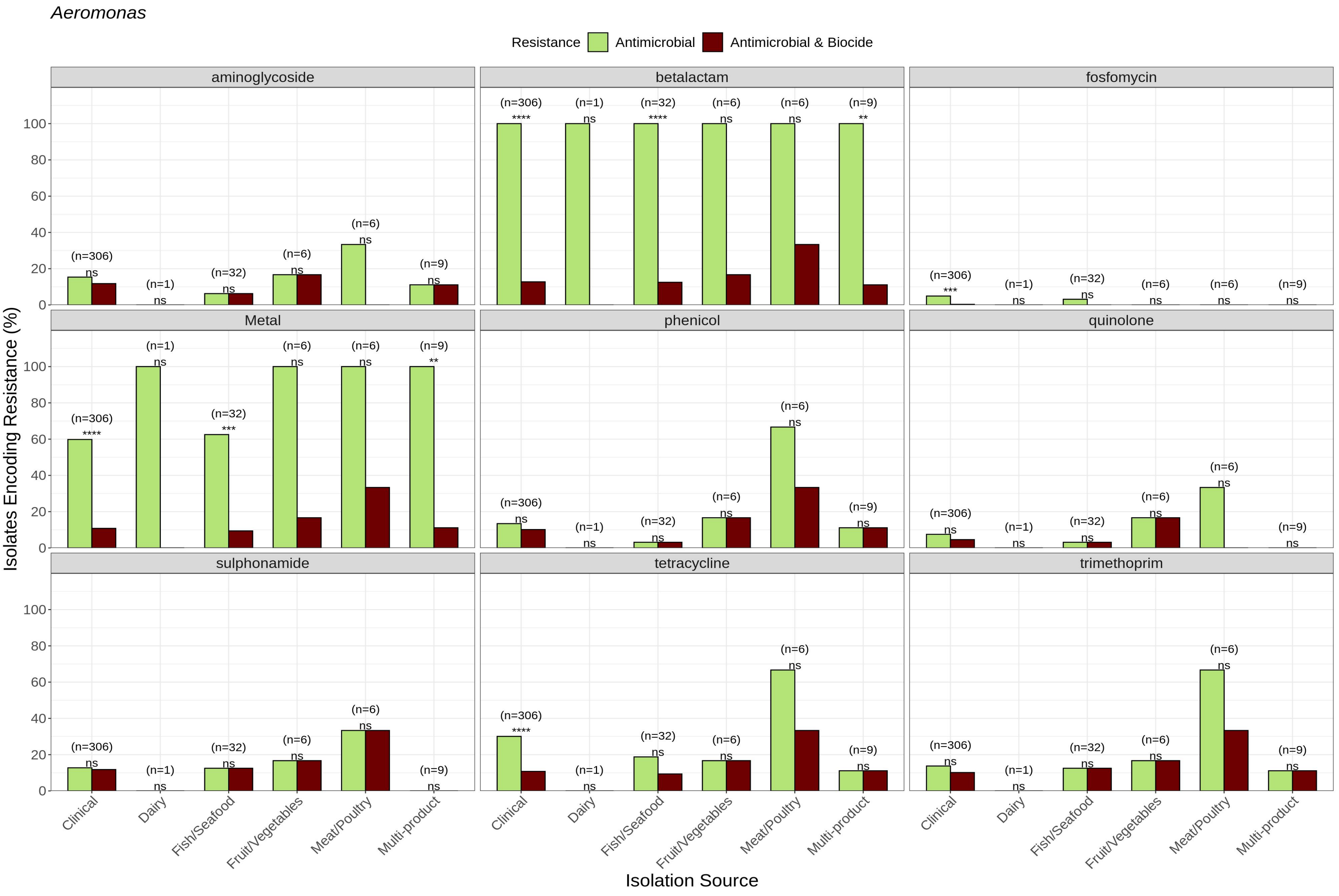
Aeromonas
Resistance
Antimicrobial
Antimicrobial & Biocide
aminoglycoside
betalactam
fosfomycin
Metal
phenicol
quinolone
sulphonamide
tetracycline
trimethoprim
Isolates Encoding Resistance (%)
Isolation Source
Clinical
Dairy
Fish/Seafood
Fruit/Vegetables
Meat/Poultry
Multi-product

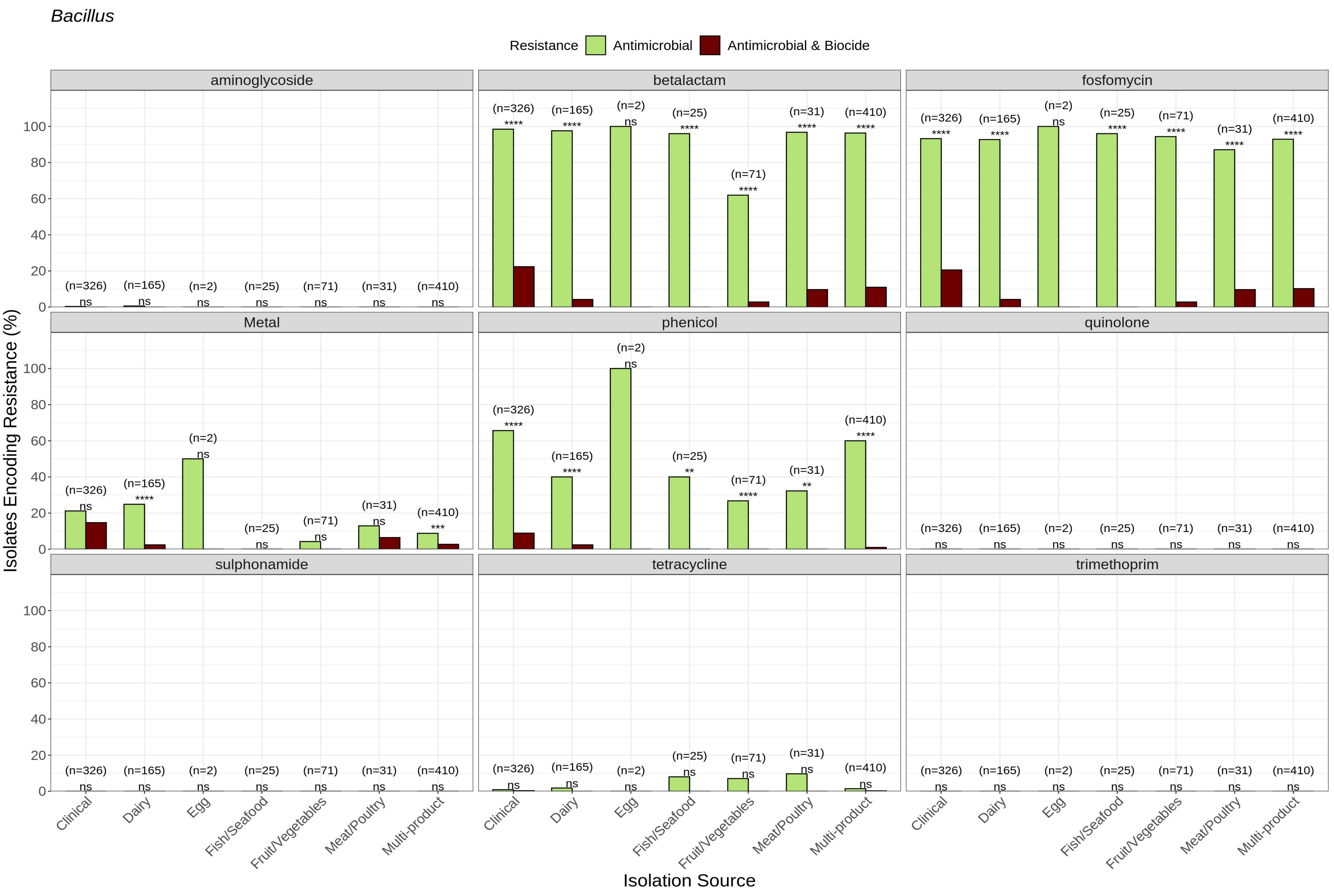
Bacillus
Resistance
Antimicrobial
Antimicrobial & Biocide
aminoglycoside
betalactam
fosfomycin
Metal
phenicol
quinolone
sulphonamide
tetracycline
trimethoprim
Isolates Encoding Resistance (%)
0
20
40
60
80
100
Clinical
Dairy
Egg
Fish/Seafood
Fruit/Vegetables
Meat/Poultry
Multi-product
Isolation Source
(n=326)
(n=165)
(n=2)
(n=25)
(n=71)
(n=31)
(n=410)
ns
****
***
**

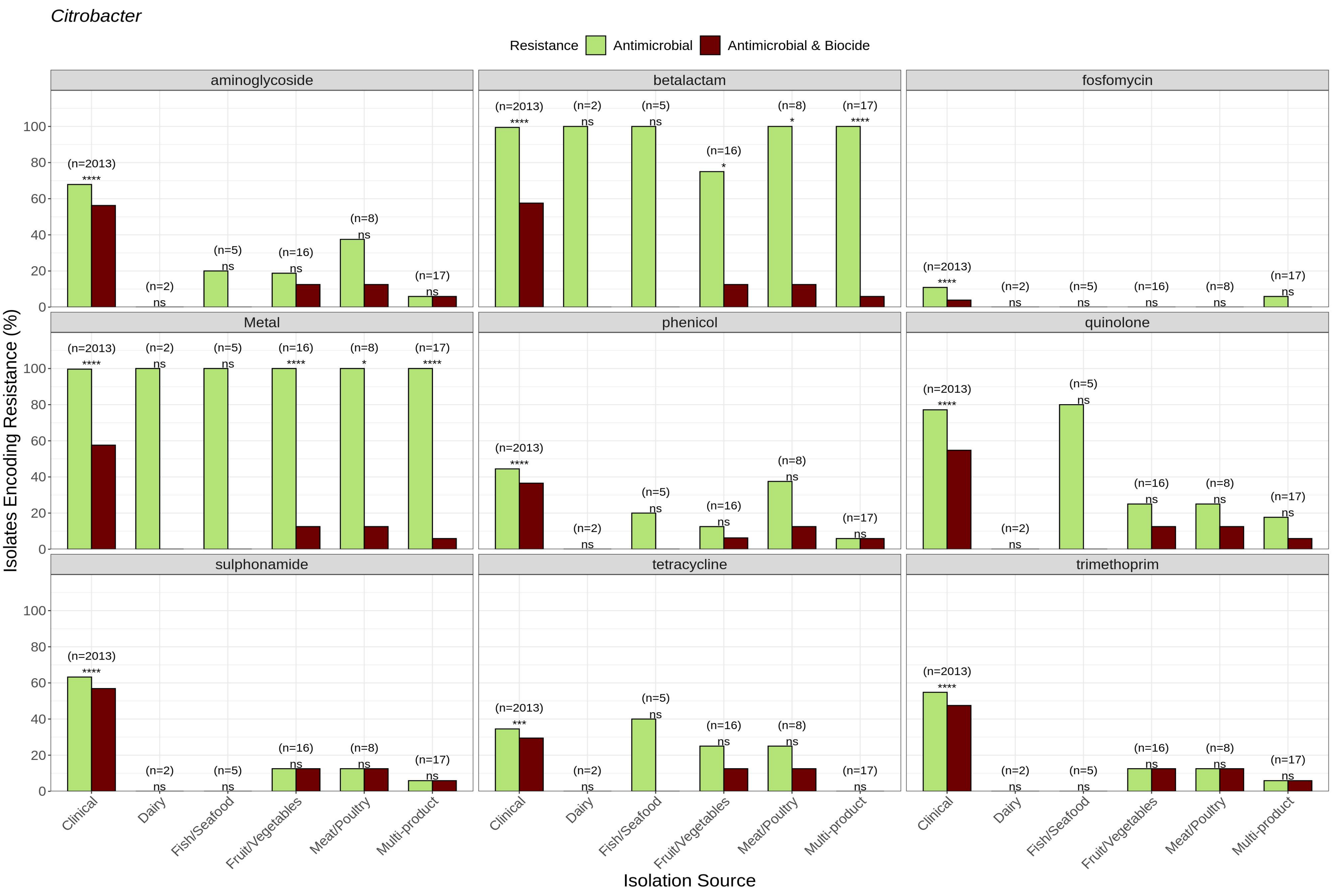
Citrobacter
Resistance
Antimicrobial
Antimicrobial & Biocide
aminoglycoside
betalactam
fosfomycin
Metal
phenicol
quinolone
sulphonamide
tetracycline
trimethoprim
Isolates Encoding Resistance (%)
Isolation Source
Clinical
Dairy
Fish/Seafood
Fruit/Vegetables
Meat/Poultry
Multi-product

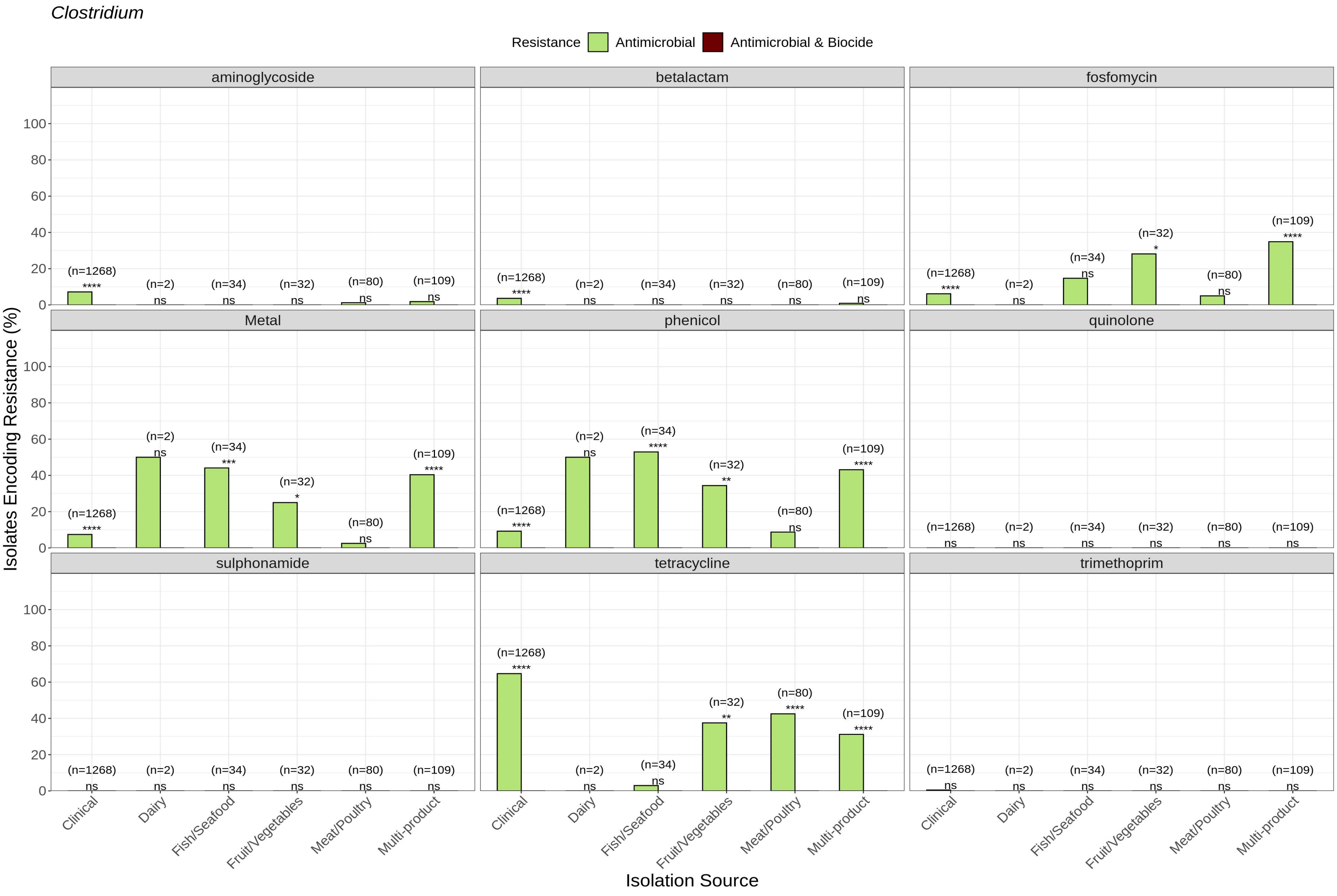
Clostridium
Resistance
Antimicrobial
Antimicrobial & Biocide
aminoglycoside
betalactam
fosfomycin
Metal
phenicol
quinolone
sulphonamide
tetracycline
trimethoprim
Isolates Encoding Resistance (%)
Isolation Source
Clinical
Dairy
Fish/Seafood
Fruit/Vegetables
Meat/Poultry
Multi-product
(n=1268)
(n=2)
(n=34)
(n=32)
(n=80)
(n=109)

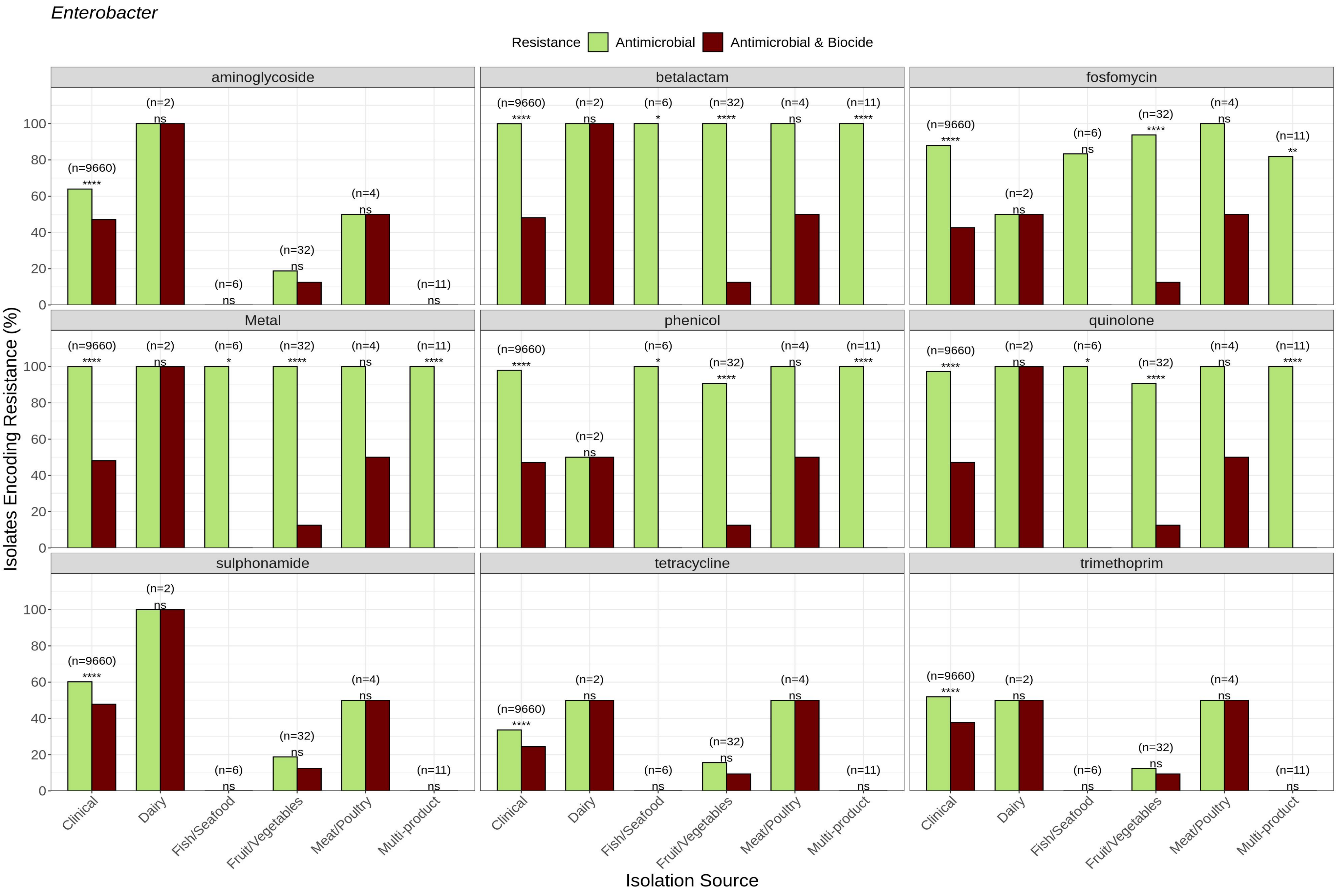
Enterobacter
Resistance
Antimicrobial
Antimicrobial & Biocide
aminoglycoside
betalactam
fosfomycin
Metal
phenicol
quinolone
sulphonamide
tetracycline
trimethoprim
Isolates Encoding Resistance (%)
Isolation Source
Clinical
Dairy
Fish/Seafood
Fruit/Vegetables
Meat/Poultry
Multi-product
(n=9660)
(n=2)
(n=6)
(n=32)
(n=4)
(n=11)

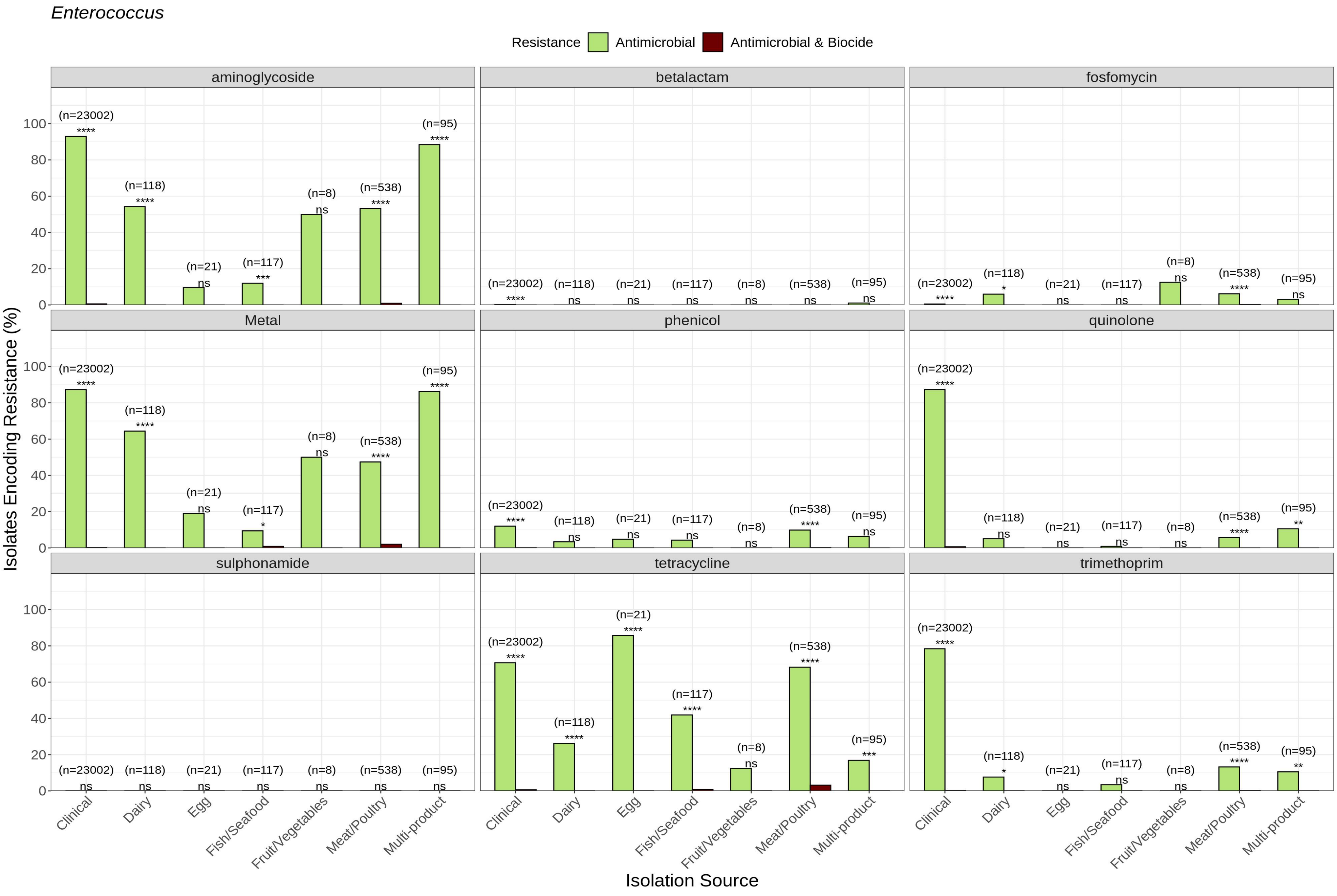

Enterococcus
Resistance
Antimicrobial
Antimicrobial & Biocide
aminoglycoside
betalactam
fosfomycin
Metal
phenicol
quinolone
sulphonamide
tetracycline
trimethoprim
Isolates Encoding Resistance (%)
Isolation Source
Clinical
Dairy
Egg
Fish/Seafood
Fruit/Vegetables
Meat/Poultry
Multi-product
(n=23002)
(n=118)
(n=21)
(n=117)
(n=8)
(n=538)
(n=95)

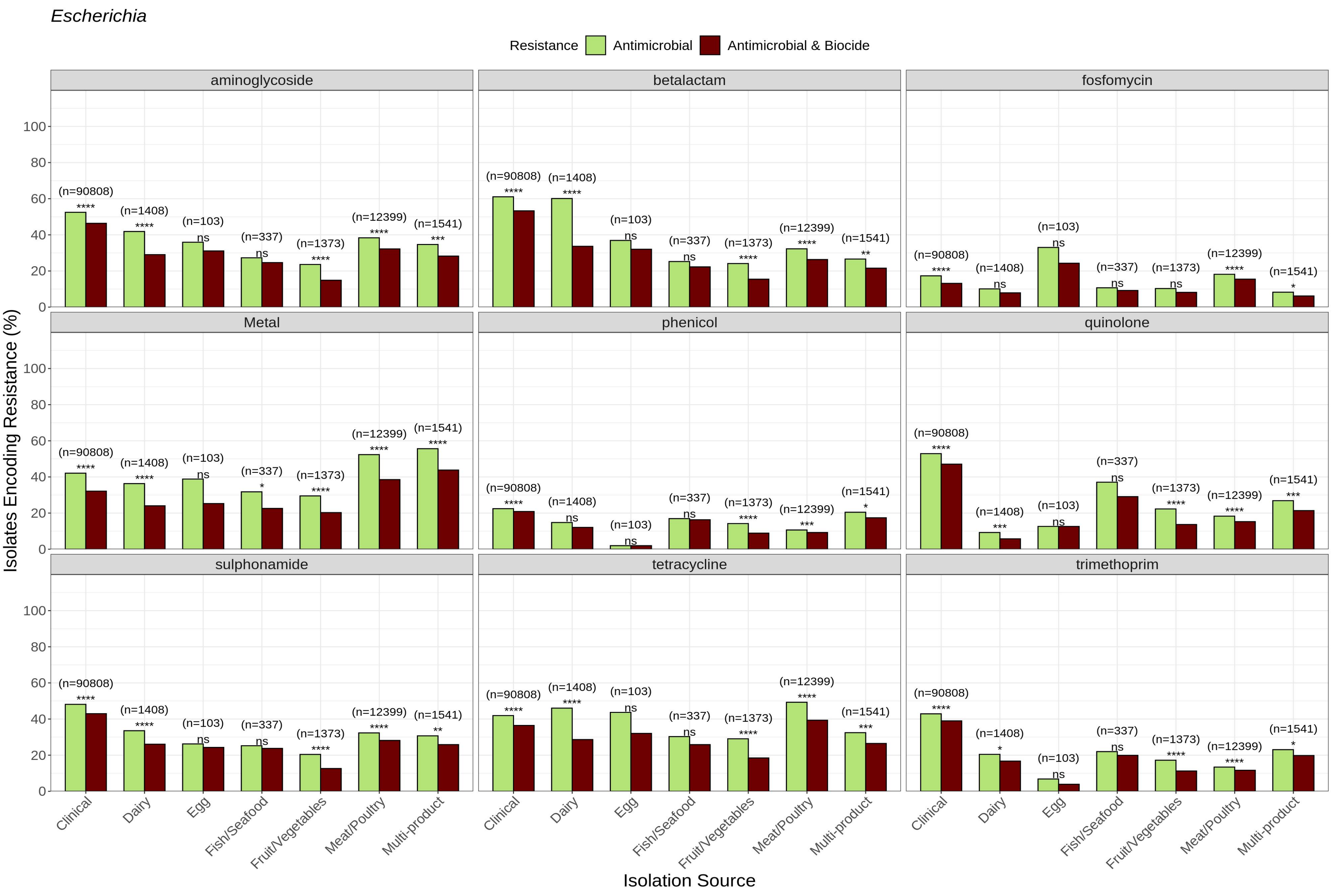
Escherichia
Resistance
Antimicrobial
Antimicrobial & Biocide
aminoglycoside
betalactam
fosfomycin
Metal
phenicol
quinolone
sulphonamide
tetracycline
trimethoprim
Isolates Encoding Resistance (%)
0
20
40
60
80
100
(n=90808)
(n=1408)
(n=103)
(n=337)
(n=1373)
(n=12399)
(n=1541)
Clinical
Dairy
Egg
Fish/Seafood
Fruit/Vegetables
Meat/Poultry
Multi-product
Isolation Source

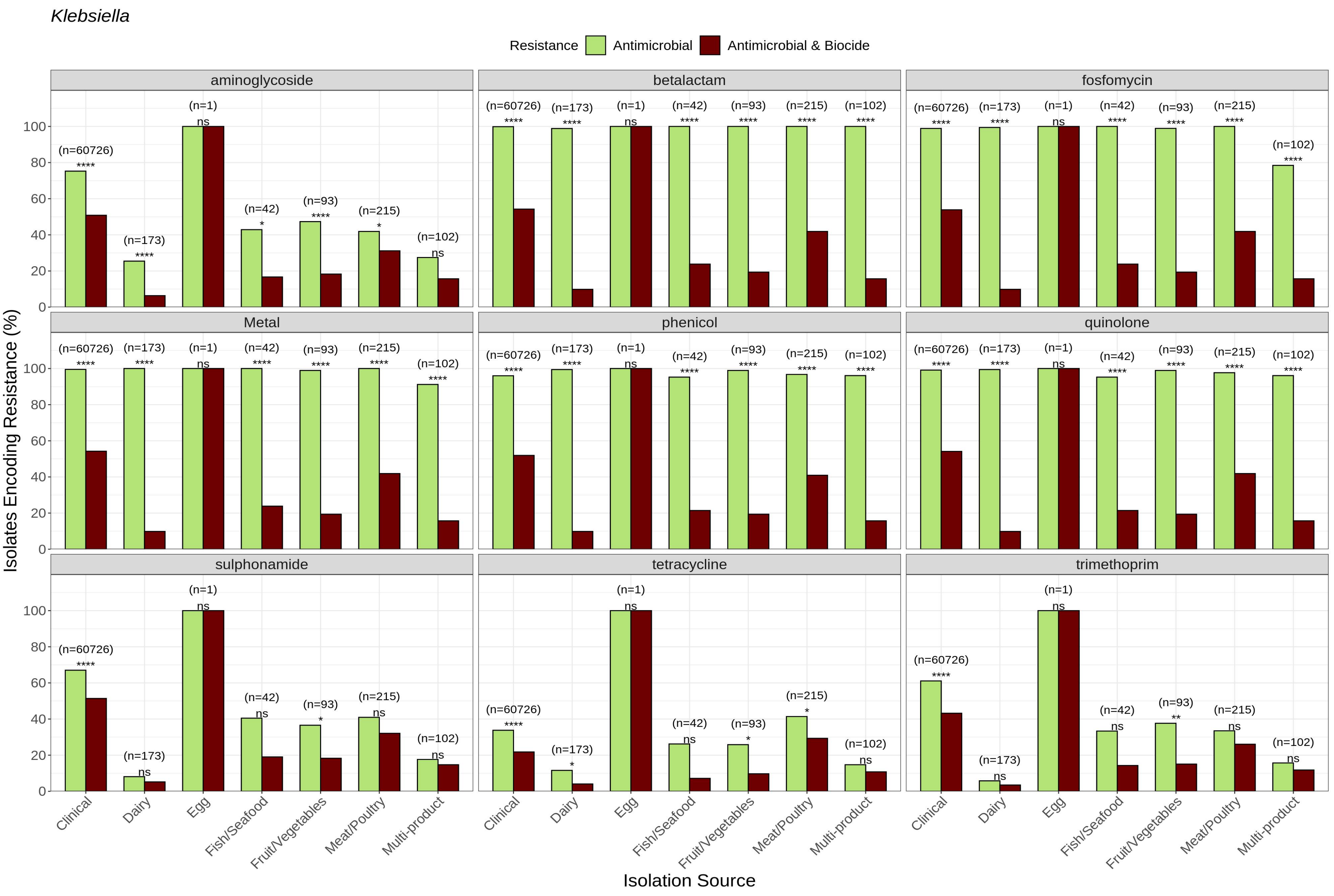
Klebsiella
Resistance
Antimicrobial
Antimicrobial & Biocide
aminoglycoside
betalactam
fosfomycin
Metal
phenicol
quinolone
sulphonamide
tetracycline
trimethoprim
Isolates Encoding Resistance (%)
Isolation Source
Clinical
Dairy
Egg
Fish/Seafood
Fruit/Vegetables
Meat/Poultry
Multi-product

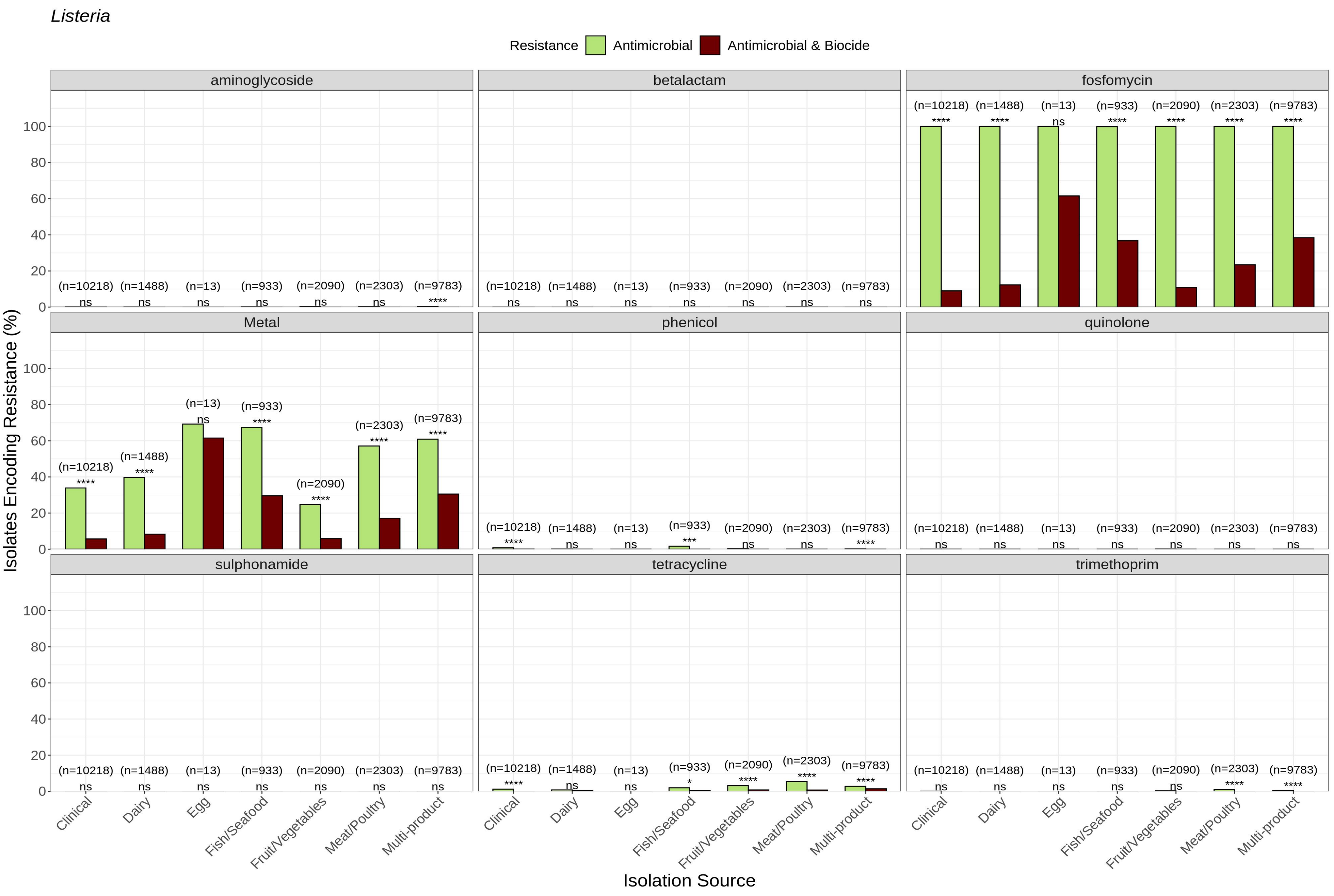
Listeria
Resistance
Antimicrobial
Antimicrobial & Biocide
aminoglycoside
betalactam
fosfomycin
Metal
phenicol
quinolone
sulphonamide
tetracycline
trimethoprim
(n=10218)
(n=1488)
(n=13)
(n=933)
(n=2090)
(n=2303)
(n=9783)
Isolates Encoding Resistance (%)
Clinical
Dairy
Egg
Fish/Seafood
Fruit/Vegetables
Meat/Poultry
Multi-product
Isolation Source

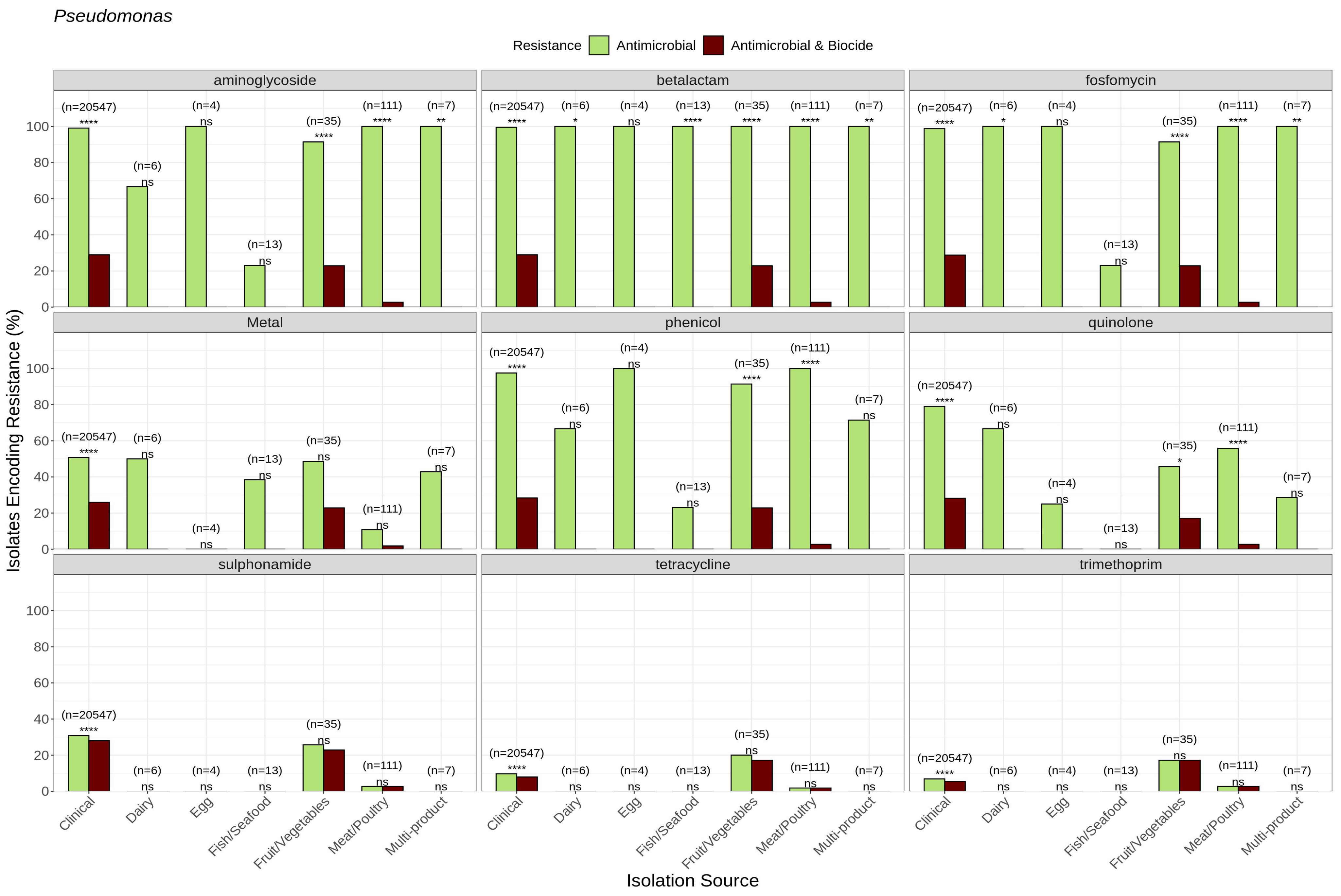
Pseudomonas
Resistance
Antimicrobial
Antimicrobial & Biocide
aminoglycoside
betalactam
fosfomycin
Metal
phenicol
quinolone
sulphonamide
tetracycline
trimethoprim
Isolates Encoding Resistance (%)
Isolation Source
Clinical
Dairy
Egg
Fish/Seafood
Fruit/Vegetables
Meat/Poultry
Multi-product
(n=20547)
(n=6)
(n=4)
(n=13)
(n=35)
(n=111)
(n=7)

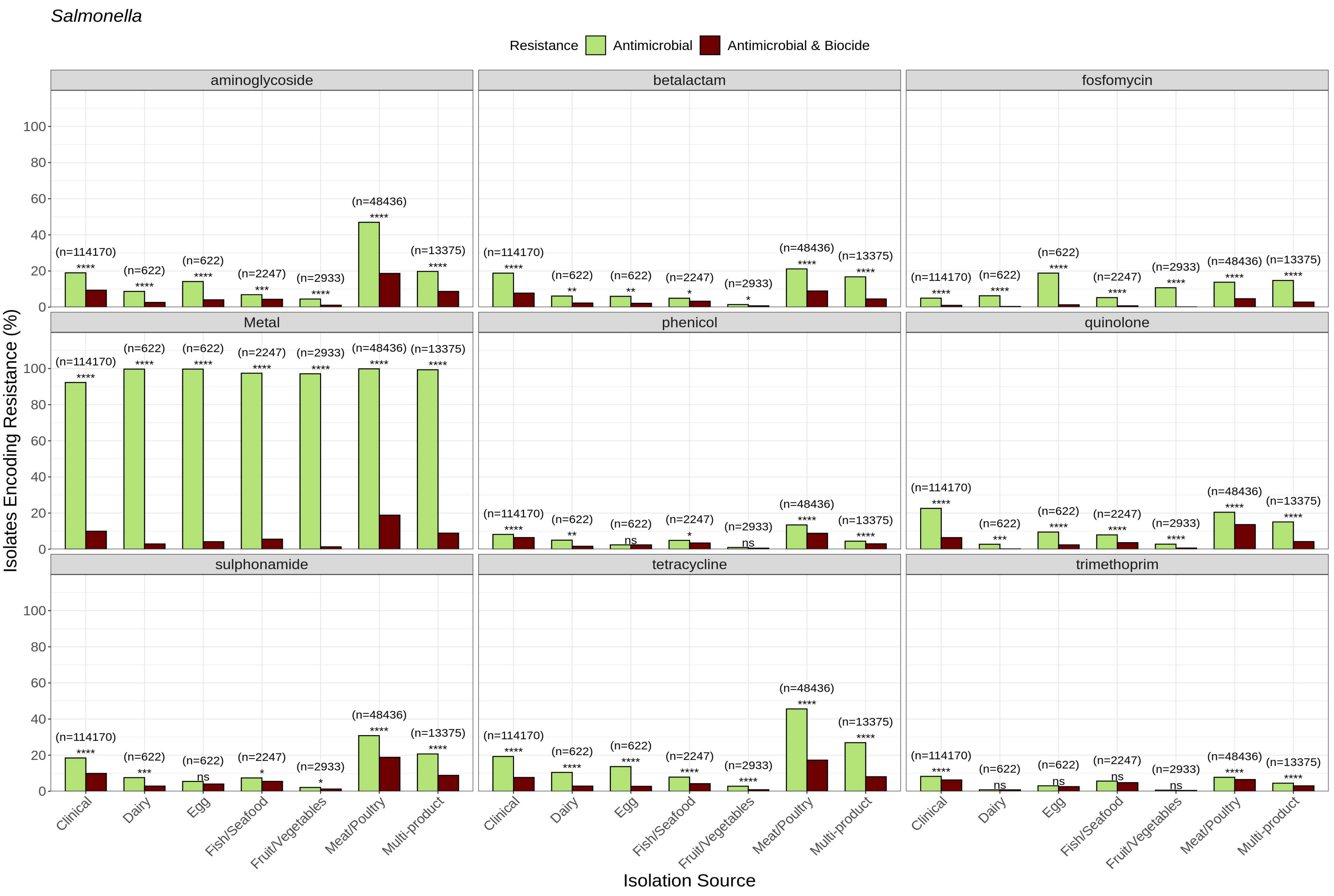
Salmonella
Resistance
Antimicrobial
Antimicrobial & Biocide
aminoglycoside
betalactam
fosfomycin
Metal
phenicol
quinolone
sulphonamide
tetracycline
trimethoprim
Isolates Encoding Resistance (%)
0
20
40
60
80
100
Clinical
Dairy
Egg
Fish/Seafood
Fruit/Vegetables
Meat/Poultry
Multi-product
Isolation Source

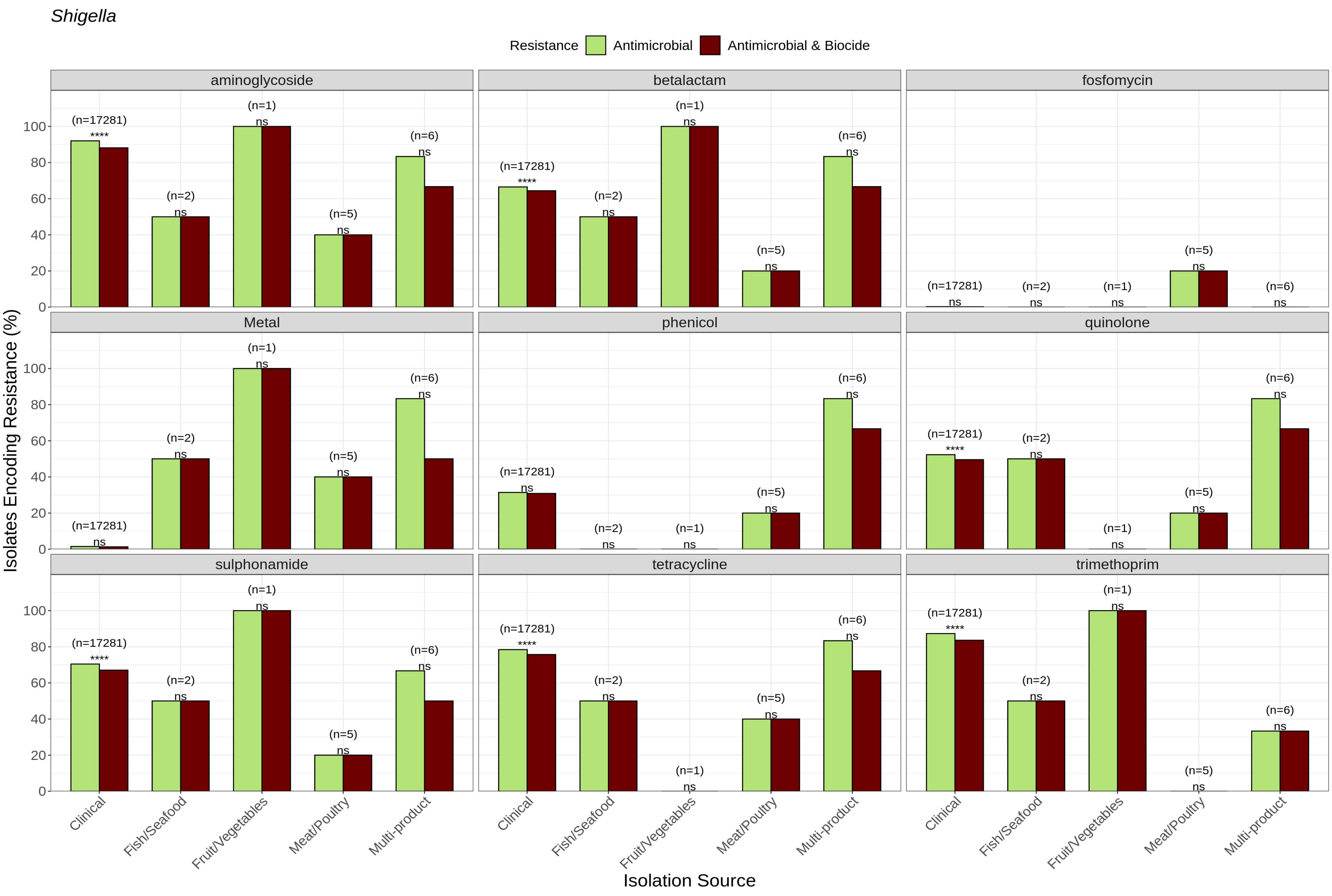
Shigella
Resistance
Antimicrobial
Antimicrobial & Biocide
aminoglycoside
betalactam
fosfomycin
Metal
phenicol
quinolone
sulphonamide
tetracycline
trimethoprim
Isolates Encoding Resistance (%)
Isolation Source
Clinical
Fish/Seafood
Fruit/Vegetables
Meat/Poultry
Multi-product
(n=17281)
(n=2)
(n=1)
(n=5)
(n=6)
ns
****

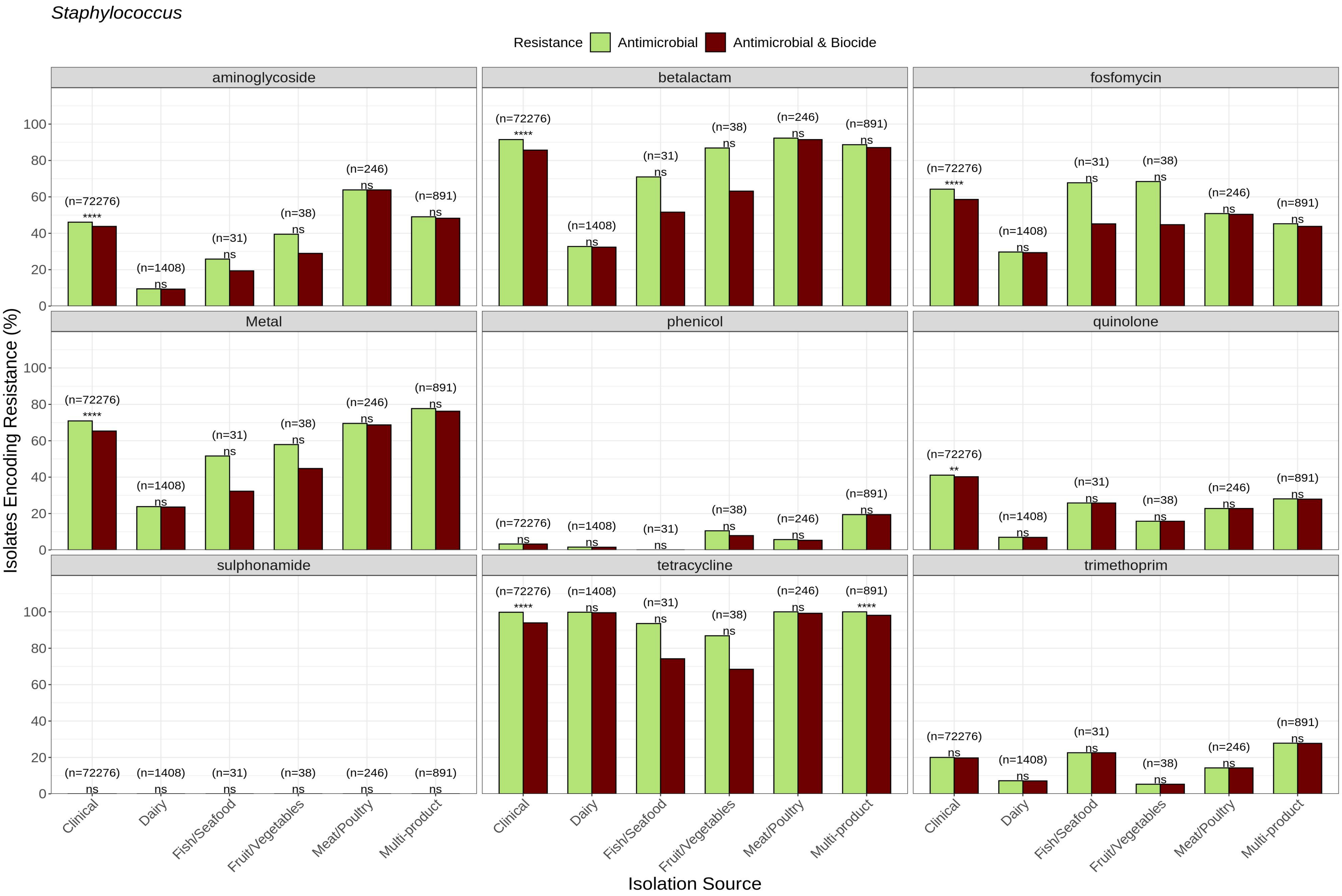

Staphylococcus
Resistance
Antimicrobial
Antimicrobial & Biocide
aminoglycoside
betalactam
fosfomycin
Metal
phenicol
quinolone
sulphonamide
tetracycline
trimethoprim
Isolates Encoding Resistance (%)
Isolation Source
Clinical
Dairy
Fish/Seafood
Fruit/Vegetables
Meat/Poultry
Multi-product
(n=72276)
(n=1408)
(n=31)
(n=38)
(n=246)
(n=891)

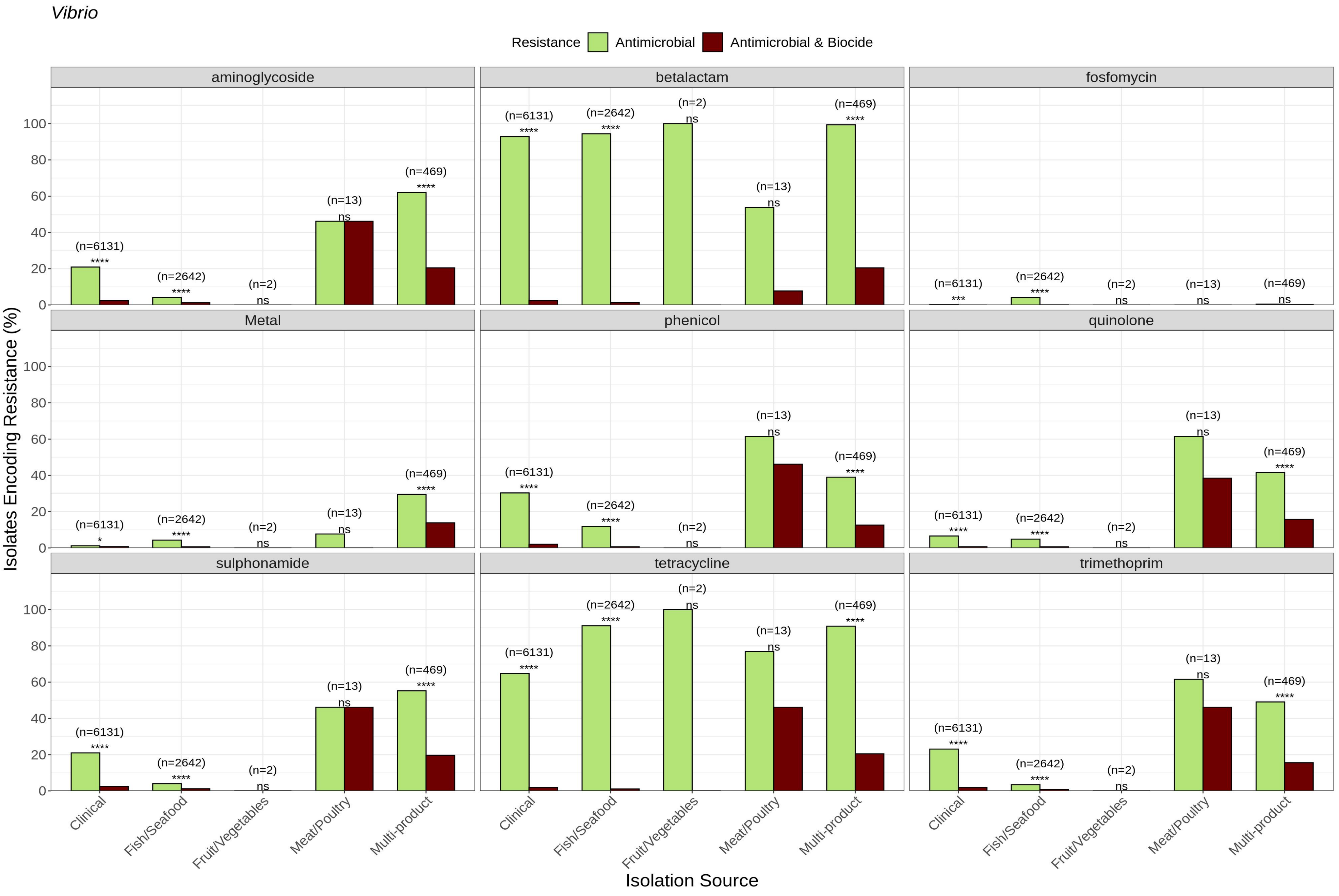
Vibrio
Resistance
Antimicrobial
Antimicrobial & Biocide
aminoglycoside
betalactam
fosfomycin
Metal
phenicol
quinolone
sulphonamide
tetracycline
trimethoprim
Isolates Encoding Resistance (%)
Isolation Source
Clinical
Fish/Seafood
Fruit/Vegetables
Meat/Poultry
Multi-product
(n=6131)
(n=2642)
(n=2)
(n=13)
(n=469)
